# Evaluation of the relationship between slow-waves of intracranial pressure, mean arterial pressure and brain tissue oxygen in TBI: a CENTER-TBI exploratory analysis

**DOI:** 10.1007/s10877-020-00527-6

**Published:** 2020-05-16

**Authors:** Frederick A. Zeiler, Manuel Cabeleira, Peter J. Hutchinson, Nino Stocchetti, Marek Czosnyka, Peter Smielewski, Ari Ercole, the CENTER-TBI High-Resolution ICU (HR ICU) Sub-Study Participants and Investigators, Audny Anke, Ronny Beer, Bo-Michael Bellander, Erta Beqiri, Andras Buki, Manuel Cabeleira, Marco Carbonara, Arturo Chieregato, Giuseppe Citerio, Hans Clusmann, Endre Czeiter, Marek Czosnyka, Bart Depreitere, Ari Ercole, Shirin Frisvold, Raimund Helbok, Stefan Jankowski, Danile Kondziella, Lars-Owe Koskinen, Ana Kowark, David K. Menon, Geert Meyfroidt, Kirsten Moeller, David Nelson, Anna Piippo-Karjalainen, Andreea Radoi, Arminas Ragauskas, Rahul Raj, Jonathan Rhodes, Saulius Rocka, Rolf Rossaint, Juan Sahuquillo, Oliver Sakowitz, Peter Smielewski, Nino Stocchetti, Nina Sundström, Riikka Takala, Tomas Tamosuitis, Olli Tenovuo, Peter Vajkoczy, Alessia Vargiolu, Rimantas Vilcinis, Stefan Wolf, Alexander Younsi, Frederick A. Zeiler

**Affiliations:** 1 Division of Anaesthesia, Addenbrooke’s Hospital, University of Cambridge, Cambridge, UK; 2 Department of Surgery, Rady Faculty of Health Sciences, University of Manitoba, Winnipeg, MB R3A 1R9 Canada; 3 Department of Human Anatomy and Cell Science, Rady Faculty of Health Sciences, University of Manitoba, Winnipeg, Canada; 4 Biomedical Engineering, Faculty of Engineering, University of Manitoba, Winnipeg, Canada; 5 Centre on Aging, University of Manitoba, Winnipeg, Canada; 6 Brain Physics Laboratory, Division of Neurosurgery, Dept of Clinical Neurosciences, Addenbrooke’s Hospital, University of Cambridge, Cambridge, CB2 0QQ UK; 7 Division of Neurosurgery, Department of Clinical Neurosciences, Addenbrooke’s Hospital, University of Cambridge, Cambridge, CB2 0QQ UK; 8 Neuro ICU Fondazione IRCCS Cà Granda Ospedale Maggiore Policlinico, Milan, Italy; 9 Department of Physiopathology and Transplantation, Milan University, Milan, Italy; 10 Institute of Electronic Systems, Warsaw University of Technology, Warsaw, Poland

**Keywords:** Autoregulation, Brain tissue oxygen, Cerebrovascular reactivity, Traumatic brain injury, TBI

## Abstract

Brain tissue oxygen (PbtO_2_) monitoring in traumatic brain injury (TBI) has demonstrated strong associations with global outcome. Additionally, PbtO_2_ signals have been used to derive indices thought to be associated with cerebrovascular reactivity in TBI. However, their true relationship to slow-wave vasogenic fluctuations associated with cerebral autoregulation remains unclear. The goal of this study was to investigate the relationship between slow-wave fluctuations of intracranial pressure (ICP), mean arterial pressure (MAP) and PbtO_2_ over time. Using the Collaborative European NeuroTrauma Effectiveness Research in Traumatic Brain Injury (CENTER-TBI) high resolution ICU sub-study cohort, we evaluated those patients with recorded high-frequency digital intra-parenchymal ICP and PbtO_2_ monitoring data of a minimum of 6 h in duration. Digital physiologic signals were processed for ICP, MAP, and PbtO_2_ slow-waves using a moving average filter to decimate the high-frequency signal. The first 5 days of recording were analyzed. The relationship between ICP, MAP and PbtO_2_ slow-waves over time were assessed using autoregressive integrative moving average (ARIMA) and vector autoregressive integrative moving average (VARIMA) modelling, as well as Granger causality testing. A total of 47 patients were included. The ARIMA structure of ICP and MAP were similar in time, where PbtO_2_ displayed different optimal structure. VARIMA modelling and IRF plots confirmed the strong directional relationship between MAP and ICP, demonstrating an ICP response to MAP impulse. PbtO_2_ slow-waves, however, failed to demonstrate a definite response to ICP and MAP slow-wave impulses. These results raise questions as to the utility of PbtO_2_ in the derivation of cerebrovascular reactivity measures in TBI. There is a reproducible relationship between slow-wave fluctuations of ICP and MAP, as demonstrated across various time-series analytic techniques. PbtO_2_ does not appear to reliably respond in time to slow-wave fluctuations in MAP, as demonstrated on various VARIMA models across all patients. These findings suggest that PbtO_2_ should not be utilized in the derivation of cerebrovascular reactivity metrics in TBI, as it does not appear to be responsive to changes in MAP in the slow-waves. These findings corroborate previous results regarding PbtO_2_ based cerebrovascular reactivity indices.

**Electronic supplementary material:**

The online version of this article (10.1007/s10877-020-00527-6) contains supplementary material, which is available to authorized users.

## Introduction

Brain tissue oxygen (PbtO_2_) monitoring in adult traumatic brain injury (TBI) is emerging as an important adjunct physiologic parameter for intensive care unit (ICU) directed therapies [1–3]. Invasively placed into the brain parenchyma, typically frontal lobe, such devices measure local extracellular partial pressure of oxygen [4, 5]. This signal provides insight into extracellular oxygen diffusion, and has an emerging literature body in adult TBI supporting its various applications. To date, numerous papers have supported the association between low PbtO_2_ measures and worse global outcome in adult TBI [1–3, 6]. Thresholds for PbtO_2_ have been suggested, with the current threshold of 20 mmHg being investigated in ongoing randomized control trials [2]. Furthermore, Phase II multi-center studies support feasibility of targeting both intracranial pressure (ICP) and PbtO_2_ thresholds of 20 mmHg, using a protocoled approach, with results from this trial supporting improved outcomes for those patients receiving both ICP and PbtO_2_ directed therapy, versus ICP directed therapy alone [2].

Another suggested application of PbtO_2_ monitoring is for cerebrovascular reactivity assessments in TBI [7–9]. Some small, mainly single center retrospective work, have derived the oxygen reactivity index (ORx) through the correlation between slow-waves of PbtO_2_ and either mean arterial pressure (MAP) or cerebral perfusion pressure (CPP) [7, 8]. This has been conducted in a similar fashion to the ICP-derived pressure reactivity index (PRx). This ORx metric can be derived based on varying window lengths of data (20, 30 or 60 min), longer than PRx (routinely 5 min long), and has literature to support its association with 6-month outcome [7, 8, 10].

However, ORx has been demonstrated in various studies of co-variance, to have no relation to more standard metrics of cerebrovascular reactivity [11, 12]. It correlates poorly with PRx [11–13], and has no association with any other multi-modal based metric of cerebrovascular reactivity during multi-variate assessments of co-variance [11]. In particular, ORx has no association with ICP and near infrared spectroscopy (NIRS) based cerebrovascular reactivity metrics [11], which, crucially, are the only such metrics to have some experimental evidence to support their ability to measure aspects of the autoregulatory curve [14–17]. Thus, the role of ORx in cerebrovascular reactivity assessment is questionable, given these previous results and the fact that PbtO_2_ represents a complex balance between oxygen supply, demand and extracellular diffusion [5, 18], not a surrogate measure of variations in cerebral blood volume (CBV) or cerebral blood flow (CBF) which are required for the derivation of continuous cerebrovascular reactivity metrics [19, 20]. Indeed in-silico simulations show that similar PbtO_2_ may be found for various different combinations of CBF, metabolic rate and diffusion.

Despite this controversy surrounding ORx, this index is still reported as a metric of cerebrovascular reactivity [7–10]. In order to facilitate understanding regarding the role of PbtO_2_ monitoring in cerebrovascular reactivity assessments, insight into the time-series relationships between ICP, MAP, CPP and PbtO_2_ slow-waves is crucial. Observations have been already presented in scientific press, indicating that transients of PbtO_2_ usually follow changes in CPP [21]. The goal of this study is to provide an exploratory analysis into the multi-variate time-series relationships between ICP, MAP and PbtO_2_ using time-series methodologies in the Collaborative European NeuroTrauma Effectiveness Research in Traumatic Brain Injury (CENTER-TBI) High-Resolution ICU (HR-ICU) sub-study cohort [22].

## Methods

### Patient population

All patients from the multi-center CENTER-TBI high resolution ICU monitoring cohort with parenchymal ICP and PbtO_2_ monitoring, were included in this analysis. Patients with EVD based ICP data were excluded given the interrupted nature of their recordings. These patients were prospectively recruited between January 2015 and December 2017 from 21 centers in the European Union (EU). All patients were admitted to ICU for their TBI during the course of the study, with high frequency digital signals recorded from their ICU monitors during the course of their ICU stay. All patients suffered predominantly from moderate to severe TBI (moderate = Glasgow Coma Score (GCS) 9 to 12, and severe = GCS of 8 or less). A minority of patients (n = 9) were categorised at the time of admission as suffering from less severe TBI, but experienced subsequent early deterioration leading to ICU admission for care and monitoring. All patients in this cohort had invasive ICP monitoring conducted in accordance with the BTF guidelines [6].

### Ethics

Data used in these analyses were collected as part of the CENTER-TBI study which had individual national or local regulatory approval; the UK Ethics approval is provided as an exemplar: (IRAS No: 150943; REC 14/SC/1370). The CENTER-TBI study (EC grant 602150) has been conducted in accordance with all relevant laws of the EU if directly applicable or of direct effect and all relevant laws of the country where the Recruiting sites were located, including but not limited to, the relevant privacy and data protection laws and regulations (the “Privacy Law”), the relevant laws and regulations on the use of human materials, and all relevant guidance relating to clinical studies from time to time in force including, but not limited to, the ICH Harmonised Tripartite Guideline for Good Clinical Practice (CPMP/ICH/135/95) (“ICH GCP”) and the World Medical Association Declaration of Helsinki entitled “Ethical Principles for Medical Research Involving Human Subjects”. Informed Consent by the patients and/or the legal representative/next of kin was obtained, accordingly to the local legislations, for all patients recruited in the Core Dataset of CENTER-TBI and documented in the e-CRF.

### Data collection

As part of recruitment to the multi-center high resolution ICU cohort of CENTER-TBI, all patients had demographics and injury data prospectively recorded. Similarly, all patients had high frequency digital signals from ICU monitoring recorded throughout their ICU stay, with the goal of initiating recording within 24 h of ICU admission. All digital ICU signals were further processed (see Signal Acquisition/Signal Processing). For the purpose of providing a description of the population for this study, basic admission demographics and centrally reported computed tomography (CT) variables for the first available CT of each patient were extracted [23]. They included: age, admission best GCS motor score and pupillary reactivity (bilaterally reactive, unilateral reactive, bilateral unreactive), Marshall CT Classification [24], Rotterdam CT score [25], presence or absence of traumatic subarachnoid haemorrhage (tSAH), extradural hematoma (EDH), pre-hospital hypotension and pre-hospital hypoxia. CENTER-TBI data version 2.1 was accessed for the purpose of this study, via Opal database software [26].

### Signal acquisition

Arterial blood pressure (ABP) was obtained through arterial lines connected to pressure transducers. ICP was acquired from an intra-parenchymal strain gauge probe (Codman ICP MicroSensor; Codman & Shurtleff Inc., Raynham, MA), parenchymal fibre optic pressure sensor (Camino ICP Monitor, Integra Life Sciences, Plainsboro, NJ, United States; https://www.integralife.com/). PbtO_2_ monitoring occurred via invasive parenchymal monitoring (Licox probe; Integra, Licox Brain Oxygen Monitoring System, Plainboro, NJ), typically placed in the frontal lobe. All signals were recorded using digital data transfer or digitized via an A/D converter (DT9803; Data Translation, Marlboro, MA), where appropriate; sampled at frequency of 100 Hz (Hz) or higher, using the ICM + software (Cambridge Enterprise Ltd, Cambridge, UK, https://icmplus.neurosurg.cam.ac.uk) or Moberg CNS Monitor (Moberg Research Inc, Ambler, PA, USA, https://www.moberg.com) or a combination of both. Signal artefacts were removed using both manual and automated methods prior to further processing or analysis.

Of note, the level of arterial line zeroing was not available for all patients in the CENTER-TBI HR ICU sub-study cohort. In general, most participating centre’s zeroed the arterial line at the level of the tragus. Regardless, for the purpose of this study and the described statistical analyses performed, the level of arterial line zeroing would not influence any of the results, only the magnitude of raw ABP values. The described analysis focuses on time-series relationships between ICP, ABP and PbtO_2_, for which a scaling error as a result of difference in zeroing applied to ABP, would not influence the statistical relationships over time for analysis conducted on a patient-by-patient basis.

### Signal processing

Post-acquisition processing of the above signals was conducted using ICM + (Cambridge Enterprise Ltd, Cambridge, UK, https://icmplus.neurosurg.cam.ac.uk). Ten second moving averages (updated every 10 s to avoid data overlap) were calculated for all recorded signals: ICP, ABP (which produced MAP), and PbtO_2_. This moving average filter was applied to decimate the raw signals to a frequency range association with the slow-wave vasogenic response of cerebrovascular reactivity.

The down-sampled and averaged data were output in 10 s update frequency (i.e. 10 s time resolution) for the entire recording period. We then limited the data for analysis to the first 5 days of recording, in order to focus on the acute phase commonly associated with cerebral physiologic derangements. All data curation and processing occurred in R (R Core Team (2019). R: A language and environment for statistical computing. R Foundation for Statistical Computing, Vienna, Austria. URL https://www.R-project.org/).

### Time series analysis and statistics

All statistical analysis was conducted using R and XLSTAT (Addinsoft, New York, NY; https://www.xlstat.com/en/) add-on package to Microsoft Excel (Microsoft Office 15, Version 16.0.7369.1323). For time series modelling first order differenced data was performed, given the non-stationary nature of the native 10-s resolution data.

### ICP, MAP and PbtO_2_ slow-wave time-series structure

Using 10-s resolution data, the following analysis was conducted for each patient using the first 5 days of recording. The optimal autoregressive integrative moving average (ARIMA) time-series structure was determined for ICP, MAP and PbtO_2_ for each individual patient using the following methodology, similar to other publications from our group [27–30]. First, autocorrelation function (ACF) and partial autocorrelation function (PACF) plots were produced, and both Augmented Dickey-Fuller (ADF) and Kwiatkowski–Phillips–Schmidt–Shin (KPSS) testing were conducted, confirming non-stationarity of ICP, MAP and PbtO_2_. First order differencing was then undertaken to remove all trend components, confirming stationarity by repeating the above-mentioned plots and testing. Next, ARIMA models were built for ICP, MAP and PbtO_2_, keeping the differencing order of 1 (i.e. d = 1) and varying both the autoregressive and moving average orders (i.e. p and q, respectively) from 0 to 4, through all respective permutations. The AIC and LL were then tabulated for each of these models, for every patient. Using the AIC and LL, the optimal ARIMA structures for ICP, MAP and PbtO_2_ were compared in each patient, with the lowest AIC and highest LL values indicating superior models. More details surrounding ARIMA modelling of time-series data can be found in the reference literature [31–33]. The general Box-Jenkin’s autoregressive moving average (ARMA) structure for ICP can be expressed as follows:

$$\mathrm{I}\mathrm{C}\mathrm{P}_{\mathrm{t}} = \mathrm{c} + \upvarepsilon_{\mathrm{t}} + {\sum }_{i=1}^{p}\varphi_{\mathrm{t}-\mathrm{i}} \mathrm{I}\mathrm{C}\mathrm{P}_{\mathrm{t}-\mathrm{i}} +{\sum }_{j=1}^{q}\theta_{\mathrm{t}-\mathrm{j}} \upvarepsilon_{\mathrm{t}-\mathrm{j}}$$

where c = constant, t = time “t”, i = integer, j = integer, p = autoregressive order, ICP = intra-cranial pressure, q = moving average order, φ = autoregressive coefficient at time “t − i”, θ = moving average coefficient at time “t − j”, ε = error term.

### Analysis of slow-wave relationships

First order differenced ICP, MAP and PbtO_2_ slow-waves were analyzed in the 10-s resolution data sheets, per patient. The co-variance of slow-waves of ICP versus MAP, PbtO_2_ versus MAP, and PbtO_2_ versus ICP, were evaluated using multi-variate vector ARIMA (VARIMA) models. Such models explore the behavior of two time series recorded simultaneously over time and are derived via extending the standard Box-Jenkin’s ARIMA models to multi-variate systems. Further description on this technique can be found in the references [31, 32]. The vector autoregressive moving average model (VARMA) of first order difference ICP and MAP can be represented by the following formula:

$$\mathrm{Y}_{\mathrm{t}} = \mathrm{C} + \mathrm{E}_{\mathrm{t}} + {\sum }_{i=1}^{p}A_{\mathrm{t}-\mathrm{i}} \mathrm{Y}_{\mathrm{t}-\mathrm{i}} +{\sum }_{j=1}^{q}B_{\mathrm{t}-\mathrm{j}} \mathrm{E}_{\mathrm{t}-\mathrm{j}}$$

where C = constant vector, t = time “t”, i = integer, j = integer, p = VARMA autoregressive order, Y_t_ = ICP or MAP at time t, q = VARMA moving average order, A = autoregressive coefficient matrix at “t − i”, B = moving average coefficient matrix at time “t − j”, E = error term vector.

We employed basic VARMA models with autoregressive order of 4 and moving average order of 4, based on the findings from individual patient ARIMA models of first order differenced ICP, MAP and PbtO_2_ data, for each patient, confirming that such VARMA orders would encompass the variation seen in optimal ARIMA structure for ICP, MAP and PbtO_2_ across the population. The coefficients derived from these VARMA models were then employed to derive impulse response function (IRF) plots between: ICP and MAP, PbtO_2_ and MAP, and PbtO_2_ and ICP. The IRF plots provide a descriptive graphical representation of the impact of one physiologic parameter on another, by using the generated VARIMA model and modelling a one standard deviation orthogonal impulse of one variable on the other, and vice versa. The plots depict how much from baseline the standard error of one variable fluctuates in response to the orthogonal impulse of the other variable, and how many lags in time it takes to recover back to baseline. Similarly, tri-variate VARIMA models were created to evaluate the concurrent relationship between ICP, MAP and PbtO_2_, with IRF plots generated for each patient.

Finally, the influence of slow-waves of ICP, MAP and PbtO_2_ on one another over time were assessed using Granger causality testing of stationary first order differenced data [29, 30, 34]. This was tested in every patient. For Granger causality, both *F*-test statistic value and *p*-values were recorded, with alpha set at 0.05. We did not correct for multiple comparisons.

## Results

### Patient characteristics

A total of 47 patients were included in this study with high-frequency ICP, MAP and PbtO_2_ physiology. The median age was 45 years (IQR 31 to 62 years), admission total GCS was 6 (IQR 3 to 10), and the admission GCS motor score was 3 (IQR 1 to 5). The median length of overall physiologic recording was 136.1 h (IQR 88.3 to 174.5 h). For those with recorded data, seven patients had bilaterally unreactive pupils, three unilateral unreactive pupil and 32 had normal pupils. Eight patients suffered pre-hospital hypoxic episodes, while 4 suffering a hypotensive episode. The median admission Marshall CT grade was 3 (IQR 2 to 6), while the median admission Rotterdam CT score was 3 (IQR 3 to 5). Finally, 35 patients had traumatic subarachnoid hemorrhage on admission CT, while 15 had an epidural hematoma present.

### ARIMA structure of ICP, MAP and PbtO_2_

Appendix A of the Supplementary Materials provides the ARIMA models tables for each patient, for ICP, MAP and PbtO_2_. The ARIMA structure was assessed on first order differenced data, from the first 5 days of physiologic recording. Each patient displayed varying optimal ARIMA orders for ICP, MAP and PbtO_2_ in keeping with individual patient heterogeneity in physiologic behavior. However, the trend seen across all 47 patients was that ICP and MAP slow-wave appeared to have similar optimal ARIMA modal structure for a given patient, while PbtO_2_ displayed a different optimal model structure with much higher autoregressive and moving average orders. This suggests that the time-series behavior of PbtO_2_ slow-waves is much different than that of ICP and MAP.

### ICP, MAP and PbtO_2_ slow-wave time series analysis

To facilitate exploration of the relationship between slow-wave fluctuations in ICP, MAP and PbtO_2_, we assessed Granger causality between the slow-waves each physiological parameter, employed VARIMA modelling of the bivariate relationships, and then the tri-variate relationship.

### Granger causality testing of slow-waves

To assess is there was a causal direction in the relationship between slow-waves of ICP and MAP, PbtO_2_ and MAP, and ICP and PbtO_2_, Granger causality testing was performed on the slow-wave physiologic data. In general, for the majority of patients testing slow-waves of ICP and MAP, MAP displayed a causal relation on ICP, as demonstrated by the larger F-statistic value favoring the MAP on ICP directionality. Similarly, for the majority of patients, MAP displayed a causal directional relationship on PbtO_2_ slow waves. Finally, for the majority of patients, ICP displayed causal impact on PbtO_2_ slow-waves. Of note, for most patients, the magnitude of the *F*-statistic values was much higher for MAP on ICP versus MAP on PbtO_2_ causal relationship. Appendix B provides the Granger causality testing for each patient, with the *F*-statistic and *p*-value reported.

### ICP versus MAP, PbtO_2_ versus MAP, and PbtO_2_ versus ICP VARIMA models

**Fig. 1:**
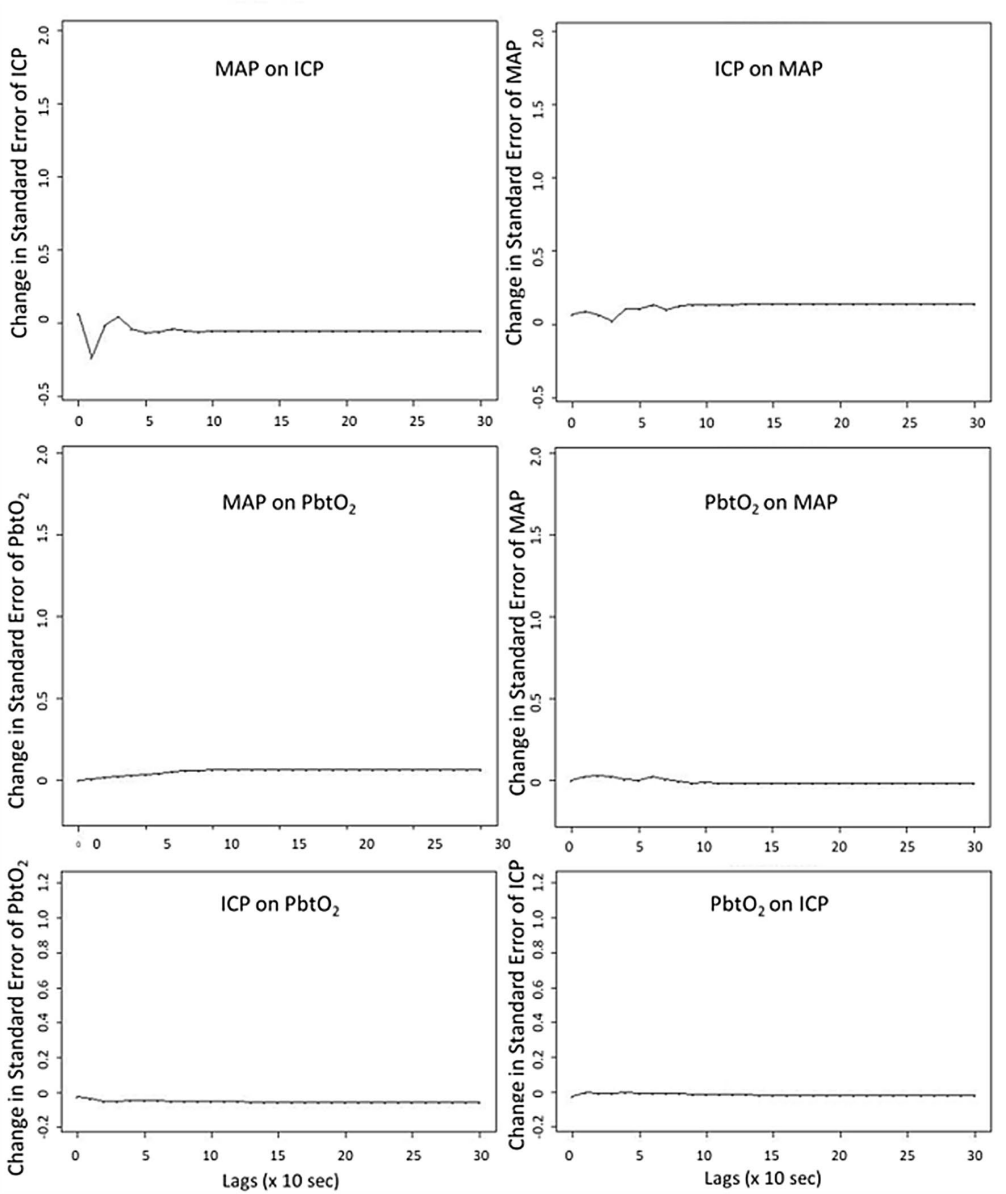
VARIMA IRF Plots for ICP/MAP, PbtO_2_/MAP, and PbtO_2_/ICP—Patient Example. *ICP* intracranial pressure, *IRF* impulse response function, *lags* refers to number of time points where 1 lag is 10-s, *MAP* mean arterial pressure, *PbtO*_*2*_ brain tissue oxygen, *VARIMA* vector autoregressive integrative moving average. All VARIMA models were constructed using an autoregressive order of 4, integrative order of 1, and moving average order of 4. The IRF plots display the response of one physiologic variable to one standard deviation impulse of the other. Of note, there is minimal response of PbtO_2_ to an impulse in MAP or ICP, with a quite extended duration low level elevation in PbtO_2_ to MAP impulse that fails to return to baseline even after 5 min worth of lags

VARIMA models with autoregressive orders of 4, integrative/differencing order of 1, and moving average order of 4, were generated for each patient, for: ICP and MAP, PbtO_2_ and MAP, and PbtO_2_ and ICP. With these models, IRF plots were generated for each patient, for each physiologic relationship. Figure 1 displays a patient example of the IRF plots for: ICP and MAP, PbtO_2_ and MAP, and PbtO_2_ and ICP. The IRF plots allow for visual assessment of the relationship between two physiologic variables, evaluating the impact of one standard deviation impulse of one physiologic variable on the other, and vice versa. For all patients, the IRF plots for MAP and ICP demonstrated a definite response of ICP to MAP. However, most patients demonstrated an attenuated response of PbtO_2_ to MAP or ICP impulses on IRF plots. Particularly the response of PbtO_2_ to MAP, where there is minimal positive change in the IRF plot, and perhaps a very extended and slow return of PbtO_2_ to baseline. This suggests that the PbtO_2_ response to MAP or ICP slow-wave fluctuations is limited, with the PbtO_2_ response to MAP occurring well past 5 min in duration based off of the IRF plots.

### ICP, MAP and PbtO_2_ VARIMA models

**Fig. 2:**
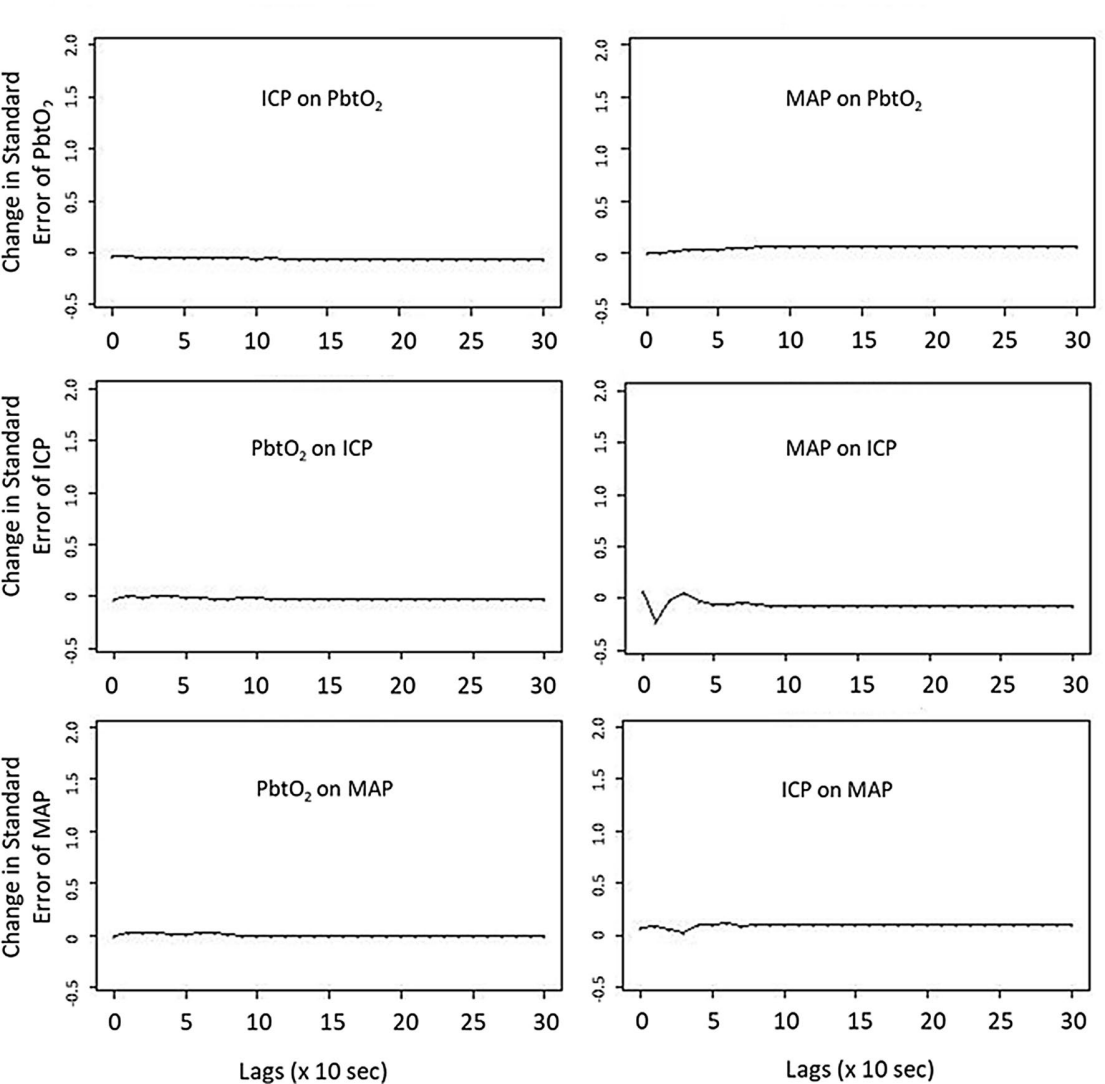
Tri-Variate VARIMA Model IRF Plot—ICP, MAP and PbtO_2_—Patient Example. *ICP* intracranial pressure, *IRF* impulse response function, *lags* refers to number of time points where 1 lag is 10-s, *MAP* mean arterial pressure, *PbtO*_*2*_ brain tissue oxygen, *VARIMA* vector autoregressive integrative moving average. The tri-variate VARIMA model was constructed using an autoregressive order of 4, integrative order of 1, and moving average order of 4. The IRF plots display the response of one physiologic variable to one standard deviation impulse of the other. Of note, there is minimal response of PbtO_2_ to an impulse in MAP or ICP

Running tri-variate VARIMA models using slow-waves of ICP, MAP and PbtO_2_, we generated IRF plots for each patient. Figure 2 provides a patient example of an IRF plot. For the majority of patients evaluated, these tri-variate models and IRF plots confirmed the relationships seen in the bi-variate VARIMA modelling. MAP demonstrated a definite response on ICP. However, ICP and MAP failed to elicit any significant fluctuations in PbtO_2_, again suggesting that PbtO_2_ fluctuations are not occurring or responding to slow-wave perturbations in MAP or ICP, commonly associated with cerebral autoregulation. Furthermore, the slight positive response of PbtO_2_ to MAP seen on IRF, extends far beyond the 5-min lag mark, reinforcing that any slight response that is seen in PbtO_2_ to MAP may be well outside of the response seen with cerebral autoregulation.

## Discussion

Using the multi-center prospectively collected CENTER-TBI HR ICU cohort, we have been able to investigate the time-series relationships between slow-waves of ICP, MAP and PbtO_2_. Overall, we have shown that PbtO_2_ does not appear to respond as ICP and MAP do to slow-wave fluctuations. This raises the question of the utility of PbtO_2_ in the derivation of cerebrovascular reactivity indices. Some important features regarding the relationship between slow-waves of these physiologic parameters deserve highlighting.

First, upon evaluating the optimal ARIMA time-series structure of ICP, MAP and PbtO_2_ slow-waves, it was clear from all patients, that the behavior of PbtO_2_ is much different than that of ICP and MAP. Though these results are preliminary, they suggest that PbtO_2_ does not appear to behave as ICP and MAP do to slow-wave perturbations, on a time scale associated with cerebrovascular reactivity/cerebral autoregulation. This is important, as it raises the question of whether PbtO_2_ signals should be used in the derivation of cerebrovascular reactivity indices such as ORx. This is probably related to a slow response time of Licox electrode readings- this way B waves of oxygenation are eliminated. In contrast, they can be seen in NIRS-derived brain mixed blood oxygenation [35].

Second, Granger causality analysis confirmed the strong directional relationship of MAP on ICP, as seen in previous work on the topic [29, 30, 34]. A similar directional relationship was seen on Granger testing for MAP on PbtO_2_, though the magnitude of the *F*-statistic was much smaller than that see for the MAP on ICP relationship. This implies that though MAP does appear to influence PbtO_2_ during periods of targeted MAP treatment, as seen in clinical intervention studies [2, 3], as the MAP on ICP relationship appears to be stronger. This is important, as it implies the MAP/ICP derived indices may provide more reliable information regarding the cerebrovascular vasogenic response, versus MAP/PbtO_2_. This of course carries implications for the calculation of PbtO_2_ derived indices, like ORx. These results corroborate the previous findings in the literature that have failed to document strong associations between ORx and ICP-derived cerebrovascular reactivity indices [11, 12]. It must be acknowledge, however, that in the setting of active and well controlled MAP/CPP in TBI care, the variations in MAP may not be sufficient enough to see a reliable and direct influence on PbtO_2_. Other small cohort studies have documented the impact of CPP (and thus MAP) changes on recorded PbtO_2_ [36]. If much larger fluctuations in MAP were seen, perhaps there would have been a more robust impact on the PbtO_2_ values seen in our cohort.

Third, applying bi-variate and tri-variate VARIMA models, we were able to derive IRF plots to aid in the evaluation of the response of ICP and PbtO_2_ to changes in MAP. As highlighted in Figs. 1 and 2, the population displayed a characteristic response of ICP slow-waves to one standard deviation impulse in MAP slow-waves, as seen in our previous work on ICP and MAP time-series [29, 30]. However, the response of PbtO_2_ slow-waves to both MAP and ICP slow-wave impulses, was virtually non-existent. This lack of response in PbtO_2_ slow-waves to impulses in ICP and MAP slow-waves, further raises the question of the utility of PbtO_2_ in the derivation of cerebrovascular reactivity indices.

In general, based on this preliminary exploratory analysis, there should be caution when using PbtO_2_ in the derivation of cerebrovascular reactivity indices. From previous literature it is becoming clearer that ORx displays little-to-no association with other multi-modal monitoring derived cerebrovascular reactivity indices [11, 12]. This includes ICP and NIRS based metrics which have some experimental literature to support their ability to measure aspects of the Lassen curve [14–17]. This current work corroborates those findings, using time-series analysis of slow-waves in ICP, MAP and PbtO_2_. These findings should not be of great surprise, given that PbtO_2_ is a measure of extracellular oxygen diffusion [5, 18], and may not respond in the same frequency range compared to ICP and MAP.

However, it must be acknowledged, that these findings in no way detract from the literature supporting the association between PbtO_2_ and ORx, with long-term outcomes in TBI [2, 3, 7, 8]. PbtO_2_ derived indices, however taking into account much slower fluctuations in raw physiology and waves than those raw signals utilized for PRx calculation, may still provide important prognostic information in TBI, and thus should not be discounted. We merely suggest that using them for the interpretation of cerebrovascular reactivity, or derivation of individualized CPP targets, should be done so with caution at this time. The results from the phase II randomized control trial comparing therapy directed at PbtO_2_ and ICP, versus ICP alone, confirm the role of PbtO_2_ in TBI care [2]. These results have subsequently sparked the ongoing phase III study, the Brain Oxygen Optimization in Traumatic Brain Injury III (BOOST-3) trial, and have received support from the recent international Seattle consensus meeting regarding TBI care guidelines [37, 38].

### Limitations

Despite the important preliminary findings in this study, there exist some limitations which deserve highlighting. First, this is an entirely exploratory analysis into the statistical time-series properties of ICP, MAP and PbtO_2_ slow-waves in a small cohort of 47 patients. Thus, the results from this analysis should remain exploratory in nature, until further validation occurs. With that said, the findings here are supported from previous analysis of ORx and its association with other cerebrovascular reactivity indices in TBI [11, 12].

Second, this cohort is one that underwent active treatment for ICP and CPP during the course of their ICU care. Thus, any administered therapies could have potentially impacted the relationships between the recorded signals and their slow-waves. Furthermore, we did not have high temporal sampling of PaO_2_ in this cohort. Thus, the relationship between arterial oxygen content and PbtO_2_ could not be commented on in this study. It is well known that PbtO_2_ levels are influenced by a variety of factors involved in oxygen uptake, transport and end-organ delivery, from the alveolar-capillary interface in the lungs, all the way to the blood–brain-barrier. Thus, anything which interferes with oxygen delivery to the alveolar-capillary interface, diffusion in the lungs, hemoglobin concentration/binding, delivery to the blood–brain-barrier (ie. cardiac output/cerebral blood flow), diffusion into the cerebral extracellular space, and end-organ use within the respiratory chain of oxidative metabolism, may have a direct impact on the recorded PbtO_2_ levels. In particular, the relationship between PaO_2_ and PbtO_2_ is well documented, were PaO_2_ levels below ~ 100 mmHg have been shown to directly impact recorded PbtO_2_ [36]. This and other systemic aspects which impact PbtO_2_ were not accounted for within this small exploratory study. However, again, despite this, the findings of our work are corroborated by previous literature on the topic.

Finally, we used 10-s resolution data for ICP, MAP and PbtO_2_. As mentioned, PbtO_2_ is a measure of extracellular oxygen diffusion, and subject to much slower frequency of physiologic response times compared to raw ICP and MAP. As such, some studies in TBI have used long windows of data in the derivation of ORx [20]. We did not evaluate the impact of window length in this study, as such large data windows evaluate ultra-low frequency below the optimal frequency ranges associated with cerebrovascular reactivity [39, 40]. At this point in time, it is uncertain if such metrics derived from long window lengths, or lower temporal resolution data, carry any information regarding cerebrovascular reactivity [29]. Consequently, we focused on 10-s-by-10-s slow-wave data.

As the goal of this study was to report the statistical relationship over time of raw recorded cerebral physiology, we did not focus on the derivation of cerebrovascular reactivity indices, and comparing between different groups based on the values of these indices. The cohort too small for subgroup analysis of this nature. In addition, the definition of who has “impaired” versus “intact” cerebrovascular reactivity in TBI is still unclear at this time. There are literature defined thresholds for certain indices (like PRx), but these are based on association with 6-month dichotomized clinical outcomes [41, 42]. These thresholds do not necessarily indicate who does or does not have impaired cerebrovascular reactivity. With that said, we have recently evaluated the insult burden of ICP, PRx and PbtO2 in our resent publication [43]. Again, here we don’t have a good definition of what defines impaired reactivity, and only are able to provide % time and dose above/below thresholds associated with global patient outcome. As such, the results of this current study are to be validated in a much larger cohort of TBI patients with PbtO_2_ monitoring, from the emerging CAHR-TBI dataset in Canada [44], with a plan to evaluate various subgroups.

The results within this study are experimental/exploratory in nature. There is a need for future validation of these results in larger cohorts with PbtO_2_ data. Similarly, if one wanted to focus entirely on the vasogenic frequency range associated with cerebrovascular reactivity, application of various band pass filtering techniques prior to signal decimation would allow for this. Further, altering the window length of physiology evaluation also needs to occur, as these relationships may be different depending on the time or day post-injury. This work is part of the planned analysis for our group and other collaborative efforts in the area of high frequency physiologic signal analysis in TBI patients. The results here provide a platform to move forward with this type of complex time-series analysis.

## Conclusions

There is a reproducible relationship between slow-wave fluctuations of ICP and MAP, as demonstrated across various time-series analytic techniques. PbtO_2_ does not appear to reliably respond in time to slow-wave fluctuations in MAP, as demonstrated on various VARIMA models across all patients. These findings suggest that PbtO_2_ should not be utilized in the derivation of cerebrovascular reactivity metrics in TBI, as it does not appear to be responsive to changes in MAP slow-waves, over a time scale commonly associated with cerebrovascular reactivity.

## Electronic supplementary material

Below is the link to the electronic supplementary material.

Supplementary file1 (DOCX 113 kb)

Supplementary file2 (DOCX 23 kb)
