# Supplementary material for: Evaluation of the relationship between slow-waves of intracranial pressure, mean arterial pressure and brain tissue oxygen in TBI: a CENTER-TBI exploratory analysis
Source: J Clin Monit Comput. 2020 May 16;35(4):711–22. doi: 10.1007/s10877-020-00527-6 (PMC8286934; doi:10.1007/s10877-020-00527-6)
Supplement: Supplementary file 1 — Supplementary file1 (DOCX 113 kb) [file 10877_2020_527_MOESM1_ESM.docx]

**Appendix A – ARIMA Tables – ICP, MAP and PbtO_2_ – Per Patient (n=47)**

**AIC – Akaike Information Criterion**

**ARIMA – autoregressive integrative moving average**

**ICP – intracranial pressure**

**LL – log likelihood**

**MAP – mean arterial pressure**

**PbtO_2_ – brain tissue oxygen**

1. Patient 1

| ARIMA Model | ICP ARIMA AIC | ICP ARIMA LL | MAP ARIMA AIC | MAP ARIMA LL | PbtO2 ARIMA AIC | PbtO2 ARIMA LL |
| --- | --- | --- | --- | --- | --- | --- |
| (0,1,0) | 102483.7 | -51239.8 | 204928.7 | -102462 | 48002.44 | -23999.2 |
| (0,1,1) | 102317.6 | -51155.8 | 204709.9 | -102352 | 18337.82 | -9165.91 |
| (0,1,2) | 97912.51 | -48952.3 | 204002.2 | -101997 | 9661.668 | -4826.83 |
| (0,1,3) | 97782.37 | -48886.2 | 203726.7 | -101858 | 5186.832 | -2588.42 |
| (0,1,4) | 97465.69 | -48726.8 | 203688.5 | -101838 | 3604.419 | -1796.21 |
| (1,1,0) | 102413.8 | -51203.9 | 204760.2 | -102377 | 2897.775 | -1445.89 |
| (1,1,1) | 100067.9 | -50030 | 203601.6 | -101797 | 2646.034 | -1319.02 |
| (1,1,2) | 97832.85 | -48911.4 | 203582 | -101786 | 2572.103 | -1281.05 |
| (1,1,3) | 97548.05 | -48768 | 203528.4 | -101758 | 2149.211 | -1068.61 |
| (1,1,4) | 97456.12 | -48721.1 | 203530.3 | -101758 | 1935.279 | -960.639 |
| (2,1,0) | 97874.15 | -48933.1 | 204078.1 | -102035 | 2611.078 | -1301.54 |
| (2,1,1) | 97523.48 | -48756.7 | 203586.1 | -101788 | 1341.852 | -665.926 |
| (2,1,2) | 97525.35 | -48756.7 | 203562.7 | -101775 | 1332.54 | -660.27 |
| (2,1,3) | 97463.64 | -48724.8 | 203526.7 | -101756 | 1268.195 | -627.097 |
| (2,1,4) | 97455.81 | -48719.9 | 203066.5 | -101525 | 1151.205 | -567.603 |
| (3,1,0) | 97536.31 | -48763.2 | 203860.4 | -101925 | 2444.133 | -1217.07 |
| (3,1,1) | 97525.1 | -48756.6 | 203645.9 | -101817 | 1334.211 | -661.106 |
| (3,1,2) | 97475.5 | -48730.8 | 203558.9 | -101772 | 1344.131 | -665.065 |
| (3,1,3) | 97434.61 | -48709.3 | 203223.4 | -101604 | 1220.509 | -602.254 |
| (3,1,4) | 97436.15 | -48709.1 | 202985.1 | -101484 | 1272.218 | -627.109 |
| (4,1,0) | 97534.9 | -48761.5 | 203816.3 | -101902 | 1737.382 | -862.691 |
| (4,1,1) | 97228.86 | -48607.4 | 203641.7 | -101814 | 1228.044 | -607.022 |
| (4,1,2) | 97215.31 | -48599.7 | 203082.1 | -101533 | 1206.769 | -595.384 |
| (4,1,3) | 97176.56 | -48579.3 | 202961.1 | -101472 | 1192.336 | -587.168 |
| (4,1,4) | 97165.46 | -48572.7 | 202916.5 | -101448 | 1181.95 | -580.975 |

1. Patient 2

| ARIMA Model | ICP ARIMA AIC | ICP ARIMA LL | MAP ARIMA AIC | MAP ARIMA LL | PbtO2 ARIMA AIC | PbtO2 ARIMA LL |
| --- | --- | --- | --- | --- | --- | --- |
| (0,1,0) | 135257.6 | -67626.8 | 145901.5 | -72948.8 | 24884.71 | -12440.4 |
| (0,1,1) | 129490 | -64742 | 141311.2 | -70652.6 | 24042.35 | -12018.2 |
| (0,1,2) | 125704.7 | -62848.3 | 140718 | -70355 | 23192.97 | -11592.5 |
| (0,1,3) | 125508.5 | -62749.2 | 140703.6 | -70346.8 | 22815.54 | -11402.8 |
| (0,1,4) | 125432.4 | -62710.2 | 140705.3 | -70346.7 | 22563.66 | -11275.8 |
| (1,1,0) | 134050.1 | -67022 | 143225.6 | -71609.8 | 23674.59 | -11834.3 |
| (1,1,1) | 127142.1 | -63567.1 | 140807.3 | -70399.7 | 22620.95 | -11306.5 |
| (1,1,2) | 125556.7 | -62773.3 | 140704.6 | -70347.3 | 22438.77 | -11214.4 |
| (1,1,3) | 125454.5 | -62721.2 | 140706.6 | -70347.3 | 22430.16 | -11209.1 |
| (1,1,4) | 125434.3 | -62710.2 | 140707.3 | -70346.6 | 22419.78 | -11202.9 |
| (2,1,0) | 128656.3 | -64324.1 | 141403.3 | -70697.7 | 22681.1 | -11336.6 |
| (2,1,1) | 125579.4 | -62784.7 | 140709.3 | -70349.6 | 22455.76 | -11222.9 |
| (2,1,2) | 125438.4 | -62713.2 | 140705.4 | -70346.7 | 22422.47 | -11205.2 |
| (2,1,3) | 125426.9 | -62706.4 | 140707.3 | -70346.7 | 22440.82 | -11213.4 |
| (2,1,4) | 125393.9 | -62689 | 140709.4 | -70346.7 | 22421.85 | -11202.9 |
| (3,1,0) | 127544.1 | -63767.1 | 140980.6 | -70485.3 | 22467.42 | -11228.7 |
| (3,1,1) | 125485.5 | -62736.8 | 140706.3 | -70347.2 | 22445 | -11216.5 |
| (3,1,2) | 125422.6 | -62704.3 | 140707.6 | -70346.8 | 22420.73 | -11203.4 |
| (3,1,3) | 125423.2 | -62703.6 | 140709.3 | -70346.7 | 22423.14 | -11203.6 |
| (3,1,4) | 125402.6 | -62692.3 | 140711.3 | -70346.7 | 22424.53 | -11203.3 |
| (4,1,0) | 126287.6 | -63137.8 | 140800.7 | -70394.4 | 22437.12 | -11212.6 |
| (4,1,1) | 125443.7 | -62714.8 | 140706.2 | -70346.1 | 22439.43 | -11212.7 |
| (4,1,2) | 125421.6 | -62702.8 | 140709.5 | -70346.8 | 22421.16 | -11202.6 |
| (4,1,3) | 125421.1 | -62701.5 | 140710.9 | -70346.4 | 22425.05 | -11203.5 |
| (4,1,4) | 125414.4 | -62697.2 | 140710.4 | -70345.2 | 22420.66 | -11200.3 |

1. Patient 3

| ARIMA Model | ICP ARIMA AIC | ICP ARIMA LL | MAP ARIMA AIC | MAP ARIMA LL | PbtO2 ARIMA AIC | PbtO2 ARIMA LL |
| --- | --- | --- | --- | --- | --- | --- |
| (0,1,0) | 126907.3 | -63451.6 | 218471 | -109233 | -37364.5 | 18684.23 |
| (0,1,1) | 116511.3 | -58252.6 | 209550.3 | -104772 | -38233 | 19119.51 |
| (0,1,2) | 116195.3 | -58093.6 | 209176.7 | -104584 | -38744.7 | 19376.36 |
| (0,1,3) | 116187.7 | -58088.8 | 209176.6 | -104583 | -39068 | 19539.01 |
| (0,1,4) | 116169.3 | -58078.6 | 209010.9 | -104499 | -39253.2 | 19632.58 |
| (1,1,0) | 120135.9 | -60065 | 212523.6 | -106259 | -38432.4 | 19219.2 |
| (1,1,1) | 116181.3 | -58086.7 | 209160.6 | -104576 | -40503.3 | 20255.63 |
| (1,1,2) | 116173 | -58081.5 | 209183.8 | -104587 | -40503.5 | 20256.77 |
| (1,1,3) | 116134.4 | -58061.2 | 209138.5 | -104563 | -40501.6 | 20256.79 |
| (1,1,4) | 116168.8 | -58077.4 | 209007.5 | -104497 | -40502.2 | 20258.11 |
| (2,1,0) | 117704.7 | -58848.4 | 210169.9 | -105081 | -39051.1 | 19529.56 |
| (2,1,1) | 116179.6 | -58084.8 | 209150.5 | -104570 | -40503.6 | 20256.79 |
| (2,1,2) | 116185.3 | -58086.7 | 209114.7 | -104551 | -40499.8 | 20255.91 |
| (2,1,3) | 116076.7 | -58031.3 | 208786.3 | -104386 | -40499.6 | 20256.81 |
| (2,1,4) | 116073.2 | -58028.6 | 208781.2 | -104383 | -40497.6 | 20256.81 |
| (3,1,0) | 116897.9 | -58443.9 | 209815.4 | -104903 | -39422.6 | 19716.28 |
| (3,1,1) | 116149.1 | -58068.6 | 208902.5 | -104445 | -40501.6 | 20256.79 |
| (3,1,2) | 116117.9 | -58052 | 208883.9 | -104435 | -40499.6 | 20256.81 |
| (3,1,3) | 116097 | -58040.5 | 208786.5 | -104385 | -40496.3 | 20256.13 |
| (3,1,4) | 116077.2 | -58029.6 | 208780.7 | -104381 | -40495.7 | 20256.87 |
| (4,1,0) | 116478.4 | -58233.2 | 209262.8 | -104625 | -39655.3 | 19833.66 |
| (4,1,1) | 116132.2 | -58059.1 | 208900.7 | -104443 | -40502.3 | 20258.16 |
| (4,1,2) | 116095 | -58039.5 | 208890.5 | -104437 | -40497.6 | 20256.81 |
| (4,1,3) | 116079.6 | -58030.8 | 208743 | -104363 | -40495.7 | 20256.87 |
| (4,1,4) | 116072.3 | -58026.2 | 208730.5 | -104355 | -40494.6 | 20257.29 |

1. Patient 4

| ARIMA Model | ICP ARIMA AIC | ICP ARIMA LL | MAP ARIMA AIC | MAP ARIMA LL | PbtO2 ARIMA AIC | PbtO2 ARIMA LL |
| --- | --- | --- | --- | --- | --- | --- |
| (0,1,0) | 59470 | -29733 | 64882.07 | -32439 | -24827.2 | 12415.6 |
| (0,1,1) | 58906.14 | -29450.1 | 64781.63 | -32387.8 | -25034.4 | 12520.22 |
| (0,1,2) | 57750.17 | -28871.1 | 64749.54 | -32370.8 | -25032.7 | 12520.37 |
| (0,1,3) | 55028.46 | -27509.2 | 64437.51 | -32213.8 | -25030.8 | 12520.38 |
| (0,1,4) | 55018.25 | -27503.1 | 64222.38 | -32105.2 | -25029.5 | 12520.74 |
| (1,1,0) | 59168.78 | -29581.4 | 64789.96 | -32392 | -25024.8 | 12515.39 |
| (1,1,1) | 58807.26 | -29399.6 | 64572.32 | -32282.2 | -25032.7 | 12520.34 |
| (1,1,2) | 56194.17 | -28092.1 | 64532.31 | -32261.2 | -25030.8 | 12520.38 |
| (1,1,3) | 55017.6 | -27502.8 | 64155.37 | -32071.7 | -25028.8 | 12520.39 |
| (1,1,4) | 54930.37 | -27458.2 | 64157.35 | -32071.7 | -25027.4 | 12520.7 |
| (2,1,0) | 57382.9 | -28687.4 | 64772.85 | -32382.4 | -25032.7 | 12520.33 |
| (2,1,1) | 55652.4 | -27821.2 | 64533.75 | -32261.9 | -25030.7 | 12520.37 |
| (2,1,2) | 55650.63 | -27819.3 | 64301.9 | -32145 | -25028.8 | 12520.39 |
| (2,1,3) | 55011.17 | -27498.6 | 64157.37 | -32071.7 | -25026.8 | 12520.39 |
| (2,1,4) | 54920.98 | -27452.5 | 64157.16 | -32070.6 | -25025.4 | 12520.71 |
| (3,1,0) | 56200.77 | -28095.4 | 64375.33 | -32182.7 | -25030.7 | 12520.36 |
| (3,1,1) | 56014.09 | -28001 | 64131.21 | -32059.6 | -25028.7 | 12520.37 |
| (3,1,2) | 55334.65 | -27660.3 | 64122.96 | -32054.5 | -25026.8 | 12520.4 |
| (3,1,3) | 54893.96 | -27439 | 64101.42 | -32042.7 | -25025.6 | 12520.8 |
| (3,1,4) | 54889.74 | -27435.9 | 64097.05 | -32039.5 | -25023.4 | 12520.71 |
| (4,1,0) | 56201.91 | -28095 | 64146.88 | -32067.4 | -25029.1 | 12520.56 |
| (4,1,1) | 55939.07 | -27962.5 | 64117.6 | -32051.8 | -25027.3 | 12520.63 |
| (4,1,2) | 55001.84 | -27492.9 | 64119.15 | -32051.6 | -25025.2 | 12520.62 |
| (4,1,3) | 54891.43 | -27436.7 | 64099.67 | -32040.8 | -25023.8 | 12520.91 |
| (4,1,4) | 54890.72 | -27435.4 | 63884.41 | -31932.2 | -25021.8 | 12520.89 |

1. Patient 5

| ARIMA Model | ICP ARIMA AIC | ICP ARIMA LL | MAP ARIMA AIC | MAP ARIMA LL | PbtO2 ARIMA AIC | PbtO2 ARIMA LL |
| --- | --- | --- | --- | --- | --- | --- |
| (0,1,0) | 38894.7 | -19445.3 | 36468.8 | -18232.4 | 32364.24 | -16180.1 |
| (0,1,1) | 38822.57 | -19408.3 | 35550.85 | -17772.4 | 32128.91 | -16061.5 |
| (0,1,2) | 34165.9 | -17079 | 35390.07 | -17691 | 32124.6 | -16058.3 |
| (0,1,3) | 34160.1 | -17075.1 | 35343.9 | -17666.9 | 32124.84 | -16057.4 |
| (0,1,4) | 33761.55 | -16874.8 | 35338.12 | -17663.1 | 32126.59 | -16057.3 |
| (1,1,0) | 38892.18 | -19443.1 | 35792.4 | -17893.2 | 32123.71 | -16058.9 |
| (1,1,1) | 38093.35 | -19042.7 | 35351 | -17671.5 | 32125.59 | -16058.8 |
| (1,1,2) | 34163.93 | -17077 | 35352.99 | -17671.5 | 32124.86 | -16057.4 |
| (1,1,3) | 34096.59 | -17042.3 | 35343.19 | -17665.6 | 32126.79 | -16057.4 |
| (1,1,4) | 33673.03 | -16829.5 | 35337.17 | -17661.6 | 32128.62 | -16057.3 |
| (2,1,0) | 35725.79 | -17858.9 | 35610.22 | -17801.1 | 32125.57 | -16058.8 |
| (2,1,1) | 33873.76 | -16931.9 | 35352.98 | -17671.5 | 32127.65 | -16058.8 |
| (2,1,2) | 33697.44 | -16842.7 | 35353.26 | -17670.6 | 32126.51 | -16057.3 |
| (2,1,3) | 33629.69 | -16807.8 | 35239.72 | -17612.9 | 32120.33 | -16053.2 |
| (2,1,4) | 33631.52 | -16807.8 | 35236.33 | -17610.2 | 32121.72 | -16052.9 |
| (3,1,0) | 35366.14 | -17678.1 | 35489.36 | -17739.7 | 32125.41 | -16057.7 |
| (3,1,1) | 33776.36 | -16882.2 | 35330.12 | -17659.1 | 32123.88 | -16055.9 |
| (3,1,2) | 33653.54 | -16819.8 | 35236.43 | -17611.2 | 32119.97 | -16053 |
| (3,1,3) | 33631.5 | -16807.8 | 35239.51 | -17611.8 | 32122.26 | -16053.1 |
| (3,1,4) | 33633.64 | -16807.8 | 35241.36 | -17611.7 | 32123.5 | -16052.8 |
| (4,1,0) | 34557.22 | -17272.6 | 35340.16 | -17664.1 | 32126.6 | -16057.3 |
| (4,1,1) | 33646.81 | -16816.4 | 35285.24 | -17635.6 | 32128.51 | -16057.3 |
| (4,1,2) | 33635.29 | -16809.6 | 35238.39 | -17611.2 | 32121.24 | -16052.6 |
| (4,1,3) | 33633.49 | -16807.7 | 35239.73 | -17610.9 | 32123.5 | -16052.8 |
| (4,1,4) | 33635.47 | -16807.7 | 35226.98 | -17603.5 | 32125.19 | -16052.6 |

1. Patient 6

| ARIMA Model | ICP ARIMA AIC | ICP ARIMA LL | MAP ARIMA AIC | MAP ARIMA LL | PbtO2 ARIMA AIC | PbtO2 ARIMA LL |
| --- | --- | --- | --- | --- | --- | --- |
| (0,1,0) | 40134.98 | -20065.5 | 48194.44 | -24095.2 | -20581.9 | 10292.94 |
| (0,1,1) | 38998.06 | -19496 | 46042.88 | -23018.4 | -23742 | 11874.02 |
| (0,1,2) | 38223.07 | -19107.5 | 45990.88 | -22991.4 | -25205.9 | 12606.96 |
| (0,1,3) | 38224.37 | -19107.2 | 45991.68 | -22990.8 | -26161.8 | 13085.89 |
| (0,1,4) | 38212.77 | -19100.4 | 45968.76 | -22978.4 | -26440.3 | 13226.16 |
| (1,1,0) | 39658.53 | -19826.3 | 46751.44 | -23372.7 | -26323.1 | 13164.54 |
| (1,1,1) | 38330.87 | -19161.4 | 45987.85 | -22989.9 | -27348.5 | 13678.25 |
| (1,1,2) | 38224.13 | -19107.1 | 45966.35 | -22978.2 | -27360.5 | 13685.26 |
| (1,1,3) | 38227 | -19107.5 | 45994.87 | -22991.4 | -27359.9 | 13685.94 |
| (1,1,4) | 38211.03 | -19098.5 | 45960.21 | -22973.1 | -27358 | 13685.99 |
| (2,1,0) | 38808.45 | -19400.2 | 46225.12 | -23108.6 | -27242 | 13625 |
| (2,1,1) | 38259.87 | -19124.9 | 45986.95 | -22988.5 | -27361.2 | 13685.59 |
| (2,1,2) | 38213.96 | -19101 | 45991.81 | -22989.9 | -27359.3 | 13685.67 |
| (2,1,3) | 38163.87 | -19074.9 | 45852.89 | -22919.4 | -27356.6 | 13685.29 |
| (2,1,4) | 38163.22 | -19073.6 | 45852.16 | -22918.1 | -27359 | 13687.5 |
| (3,1,0) | 38651.49 | -19320.7 | 46123.01 | -23056.5 | -27345.7 | 13677.83 |
| (3,1,1) | 38217.02 | -19102.5 | 45958.82 | -22973.4 | -27359.5 | 13685.75 |
| (3,1,2) | 38211.86 | -19098.9 | 45855.24 | -22920.6 | -27357.7 | 13685.83 |
| (3,1,3) | 38214.17 | -19099.1 | 45851.96 | -22918 | -27422.3 | 13719.17 |
| (3,1,4) | 38200.18 | -19091.1 | 45860.21 | -22921.1 | -27420.4 | 13719.18 |
| (4,1,0) | 38457.93 | -19223 | 46070.58 | -23029.3 | -27349.8 | 13680.92 |
| (4,1,1) | 38215.24 | -19100.6 | 45892.09 | -22939 | -27343.5 | 13678.74 |
| (4,1,2) | 38213.63 | -19098.8 | 45847.32 | -22915.7 | -27357.2 | 13686.58 |
| (4,1,3) | 38215.82 | -19098.9 | 45855.68 | -22918.8 | -27420.4 | 13719.18 |
| (4,1,4) | 38182.79 | -19081.4 | 45855.42 | -22917.7 | -27418.5 | 13719.25 |

1. Patient 7

| ARIMA Model | ICP ARIMA AIC | ICP ARIMA LL | MAP ARIMA AIC | MAP ARIMA LL | PbtO2 ARIMA AIC | PbtO2 ARIMA LL |
| --- | --- | --- | --- | --- | --- | --- |
| (0,1,0) | 152170.1 | -76083 | 182611.1 | -91303.6 | 966.0272 | -481.014 |
| (0,1,1) | 152137.3 | -76065.6 | 180092.5 | -90043.2 | -847.739 | 426.8695 |
| (0,1,2) | 147628.8 | -73810.4 | 177289.9 | -88641 | -1954.98 | 981.4923 |
| (0,1,3) | 147255.5 | -73622.7 | 177268.1 | -88629 | -2162.68 | 1086.338 |
| (0,1,4) | 147218.5 | -73603.3 | 176369.3 | -88178.6 | -2172.02 | 1092.009 |
| (1,1,0) | 152157.5 | -76075.8 | 181325.6 | -90659.8 | -1624.74 | 815.3701 |
| (1,1,1) | 148883.5 | -74437.8 | 177685.1 | -88838.6 | -1856.31 | 932.1541 |
| (1,1,2) | 147198.7 | -73594.4 | 177234.3 | -88612.1 | -2147.71 | 1078.855 |
| (1,1,3) | 146936.1 | -73462.1 | 177224.8 | -88606.4 | -2168.43 | 1090.214 |
| (1,1,4) | 146783.1 | -73384.5 | 175352.3 | -87669.2 | -2172.38 | 1093.189 |
| (2,1,0) | 148792.3 | -74392.2 | 177480.3 | -88736.2 | -1995.6 | 1001.8 |
| (2,1,1) | 147546 | -73768 | 177040.2 | -88515.1 | -2043.95 | 1026.974 |
| (2,1,2) | 147187 | -73587.5 | 176877.5 | -88432.8 | -2192.38 | 1102.192 |
| (2,1,3) | 146789 | -73387.5 | 175967.8 | -87976.9 | -2204.89 | 1109.444 |
| (2,1,4) | 146798.8 | -73391.4 | 175262.7 | -87623.4 | -2202.77 | 1109.387 |
| (3,1,0) | 148476.7 | -74233.3 | 177100.1 | -88545 | -2096.02 | 1053.008 |
| (3,1,1) | 147083.6 | -73535.8 | 177020 | -88504 | -2181.95 | 1096.976 |
| (3,1,2) | 146965.2 | -73475.6 | 175158.7 | -87572.4 | -2202.76 | 1108.382 |
| (3,1,3) | 147142.4 | -73563.2 | 174027.3 | -87005.7 | -2193.04 | 1104.521 |
| (3,1,4) | 147039.1 | -73510.5 | 174010.8 | -86996.4 | -2200.85 | 1109.423 |
| (4,1,0) | 147687.1 | -73837.5 | 176749.2 | -88368.6 | -2197.87 | 1104.933 |
| (4,1,1) | 147075.8 | -73530.9 | 174617.6 | -87301.8 | -2203.69 | 1108.843 |
| (4,1,2) | 147081 | -73532.5 | 174602.1 | -87293.1 | -2202.32 | 1109.161 |
| (4,1,3) | 147117.1 | -73549.5 | 174006.6 | -86994.3 | -2199.44 | 1108.72 |
| (4,1,4) | 147037.8 | -73508.9 | 173989.2 | -86984.6 | -2198.74 | 1109.37 |

1. Patient 8

| ARIMA Model | ICP ARIMA AIC | ICP ARIMA LL | MAP ARIMA AIC | MAP ARIMA LL | PbtO2 ARIMA AIC | PbtO2 ARIMA LL |
| --- | --- | --- | --- | --- | --- | --- |
| (0,1,0) | 150506 | -75251 | 415845.1 | -207921 | 86702.61 | -43349.3 |
| (0,1,1) | 146621 | -73307.5 | 412481.8 | -206238 | 75954.31 | -37974.2 |
| (0,1,2) | 141905.2 | -70948.6 | 409102.3 | -204547 | 70327.59 | -35159.8 |
| (0,1,3) | 141629.3 | -70809.7 | 409058.5 | -204524 | 68341.82 | -34165.9 |
| (0,1,4) | 141630.9 | -70809.4 | 409046.4 | -204517 | 67300.6 | -33644.3 |
| (1,1,0) | 148447.2 | -74220.6 | 413904.4 | -206949 | 70281.41 | -35137.7 |
| (1,1,1) | 142319.1 | -71155.6 | 409750 | -204871 | 67373.62 | -33682.8 |
| (1,1,2) | 141652.5 | -70821.3 | 409064.3 | -204527 | 66920.99 | -33455.5 |
| (1,1,3) | 141631 | -70809.5 | 409051.5 | -204520 | 66906.47 | -33447.2 |
| (1,1,4) | 141633.1 | -70809.6 | 409049.2 | -204518 | 66858.28 | -33422.1 |
| (2,1,0) | 144465.4 | -72228.7 | 410266.4 | -205129 | 67001.39 | -33496.7 |
| (2,1,1) | 141617 | -70803.5 | 409047.6 | -204519 | 66969.69 | -33479.8 |
| (2,1,2) | 141618.9 | -70803.5 | 409045.1 | -204517 | 66860.56 | -33424.3 |
| (2,1,3) | 141633.6 | -70809.8 | 409048.8 | -204517 | 66924.75 | -33455.4 |
| (2,1,4) | 141610.2 | -70797.1 | 409050.6 | -204517 | 66861.3 | -33422.6 |
| (3,1,0) | 142845.1 | -71417.5 | 409427.6 | -204709 | 66964.11 | -33477.1 |
| (3,1,1) | 141618.8 | -70803.4 | 409045 | -204517 | 66963.64 | -33475.8 |
| (3,1,2) | 141621 | -70803.5 | 409047.9 | -204517 | 66828.9 | -33407.5 |
| (3,1,3) | 141622.7 | -70803.4 | 409049.1 | -204517 | 66795.45 | -33389.7 |
| (3,1,4) | 141622.7 | -70802.3 | 409051.5 | -204517 | 66871.66 | -33426.8 |
| (4,1,0) | 142048.9 | -71018.4 | 409160.1 | -204574 | 66954.06 | -33471 |
| (4,1,1) | 141620.3 | -70803.1 | 409047 | -204517 | 66965.73 | -33475.9 |
| (4,1,2) | 141622.5 | -70803.2 | 409049 | -204517 | 66933.4 | -33458.7 |
| (4,1,3) | 141611.8 | -70796.9 | 409051 | -204516 | 66573.86 | -33277.9 |
| (4,1,4) | 141622.9 | -70801.5 | 409052.9 | -204516 | 66701.38 | -33340.7 |

1. Patient 9

| ARIMA Model | ICP ARIMA AIC | ICP ARIMA LL | MAP ARIMA AIC | MAP ARIMA LL | PbtO2 ARIMA AIC | PbtO2 ARIMA LL |
| --- | --- | --- | --- | --- | --- | --- |
| (0,1,0) | 149408.8 | -74702.4 | 203785.4 | -101891 | -6632.54 | 3318.272 |
| (0,1,1) | 143029.7 | -71511.9 | 203779.8 | -101887 | -13432.9 | 6719.474 |
| (0,1,2) | 137106.1 | -68549.1 | 196987.4 | -98489.7 | -16601.3 | 8304.656 |
| (0,1,3) | 137102.2 | -68546.1 | 196017.5 | -98003.8 | -17810.6 | 8910.301 |
| (0,1,4) | 136803.9 | -68395.9 | 195668.2 | -97828.1 | -18455.7 | 9233.853 |
| (1,1,0) | 148320.6 | -74157.3 | 203785.3 | -101890 | -17199.9 | 8602.952 |
| (1,1,1) | 138931.8 | -69461.9 | 203101.8 | -101547 | -19187.6 | 9597.782 |
| (1,1,2) | 137104.2 | -68547.1 | 196444.7 | -98217.3 | -19255.7 | 9632.847 |
| (1,1,3) | 137051.9 | -68520 | 195832.8 | -97910.4 | -19255.3 | 9633.66 |
| (1,1,4) | 136805.7 | -68395.8 | 195582.6 | -97784.3 | -19278.3 | 9646.126 |
| (2,1,0) | 142189.4 | -71090.7 | 198010.9 | -99001.5 | -19099.2 | 9553.6 |
| (2,1,1) | 136823.5 | -68406.8 | 195590.7 | -97790.4 | -19253.2 | 9631.598 |
| (2,1,2) | 136787 | -68387.5 | 195589.4 | -97788.7 | -19652.9 | 9832.442 |
| (2,1,3) | 136788.3 | -68387.1 | 195459.8 | -97722.9 | -19252.1 | 9633.061 |
| (2,1,4) | 136790.3 | -68387.1 | 195461.6 | -97722.8 | -19257.2 | 9636.59 |
| (3,1,0) | 140038.7 | -70014.3 | 196151.6 | -98070.8 | -19241.9 | 9625.94 |
| (3,1,1) | 136785.8 | -68386.9 | 195587.1 | -97787.6 | -19252.2 | 9632.094 |
| (3,1,2) | 136788.3 | -68387.1 | 195398.9 | -97692.4 | -19249.2 | 9631.611 |
| (3,1,3) | 136789.1 | -68386.6 | 195461.7 | -97722.9 | -19241.8 | 9628.909 |
| (3,1,4) | 136786.2 | -68384.1 | 195463.4 | -97722.7 | -19248.6 | 9633.298 |
| (4,1,0) | 138735.5 | -69361.7 | 195965.9 | -97977 | -19255.4 | 9633.709 |
| (4,1,1) | 136787.5 | -68386.8 | 195467.6 | -97726.8 | -19250.4 | 9632.221 |
| (4,1,2) | 136789.4 | -68386.7 | 195450.1 | -97717.1 | -19249.9 | 9632.948 |
| (4,1,3) | 136789.5 | -68385.8 | 195118.3 | -97550.1 | -19245.3 | 9631.663 |
| (4,1,4) | 136791.4 | -68385.7 | 195285.2 | -97632.6 | -19743.9 | 9881.941 |

1. Patient 10

| ARIMA Model | ICP ARIMA AIC | ICP ARIMA LL | MAP ARIMA AIC | MAP ARIMA LL | PbtO2 ARIMA AIC | PbtO2 ARIMA LL |
| --- | --- | --- | --- | --- | --- | --- |
| (0,1,0) | 216800.1 | -108398 | 277905.6 | -138951 | 23474.34 | -11735.2 |
| (0,1,1) | 214604.1 | -107299 | 277475.2 | -138735 | 853.679 | -423.839 |
| (0,1,2) | 212052.2 | -106022 | 277463.6 | -138728 | -6737.68 | 3372.842 |
| (0,1,3) | 211951.1 | -105971 | 277061.5 | -138526 | -7267.21 | 3638.605 |
| (0,1,4) | 211811 | -105900 | 277032.1 | -138510 | -7355.13 | 3683.564 |
| (1,1,0) | 215493.6 | -107744 | 277496.2 | -138745 | -4761.3 | 2383.649 |
| (1,1,1) | 212558 | -106275 | 277472.6 | -138732 | -6120.6 | 3064.299 |
| (1,1,2) | 211988.1 | -105989 | 277371.4 | -138681 | -7313.92 | 3661.959 |
| (1,1,3) | 211880.6 | -105934 | 277042.3 | -138515 | -7317.58 | 3664.791 |
| (1,1,4) | 211786.1 | -105886 | 277022.4 | -138504 | -7363.08 | 3688.542 |
| (2,1,0) | 212882.8 | -106437 | 277423.9 | -138708 | -6706.7 | 3357.349 |
| (2,1,1) | 211854 | -105922 | 277329 | -138660 | -7095.07 | 3552.534 |
| (2,1,2) | 211769.3 | -105879 | 277095.9 | -138542 | -7338.35 | 3675.177 |
| (2,1,3) | 211770.4 | -105878 | 277018.9 | -138502 | -7413.36 | 3713.679 |
| (2,1,4) | 211770.4 | -105877 | 277007.8 | -138496 | -7479.68 | 3747.842 |
| (3,1,0) | 211866.5 | -105928 | 277102.9 | -138546 | -7301.64 | 3655.82 |
| (3,1,1) | 211773.2 | -105881 | 277041.1 | -138515 | -7462.99 | 3737.497 |
| (3,1,2) | 211771.3 | -105879 | 277012.7 | -138499 | -7461.29 | 3737.646 |
| (3,1,3) | 211751.8 | -105868 | 277007.6 | -138496 | -7470.28 | 3743.138 |
| (3,1,4) | 211740.4 | -105861 | 276992.8 | -138487 | -7480.1 | 3749.051 |
| (4,1,0) | 211781.1 | -105885 | 277011.4 | -138500 | -7414.77 | 3713.387 |
| (4,1,1) | 211774.4 | -105880 | 277008.3 | -138497 | -7461.06 | 3737.53 |
| (4,1,2) | 211777.2 | -105881 | 277004.7 | -138494 | -7459.45 | 3737.725 |
| (4,1,3) | 211742.5 | -105862 | 276998.6 | -138490 | -7474.13 | 3746.063 |
| (4,1,4) | 211744.6 | -105862 | 277007.1 | -138494 | -7476.41 | 3748.206 |

1. Patient 11

| ARIMA Model | ICP ARIMA AIC | ICP ARIMA LL | MAP ARIMA AIC | MAP ARIMA LL | PbtO2 ARIMA AIC | PbtO2 ARIMA LL |
| --- | --- | --- | --- | --- | --- | --- |
| (0,1,0) | 31823.77 | -15909.9 | 70413.1 | -35204.6 | 37747.9 | -18871.9 |
| (0,1,1) | 29858.1 | -14926 | 66819.92 | -33407 | 36820.68 | -18407.3 |
| (0,1,2) | 29831.88 | -14911.9 | 66422.39 | -33207.2 | 36618.98 | -18305.5 |
| (0,1,3) | 29680.25 | -14835.1 | 66399.19 | -33194.6 | 36468.64 | -18229.3 |
| (0,1,4) | 29631.17 | -14809.6 | 66362.73 | -33175.4 | 36387.05 | -18187.5 |
| (1,1,0) | 29846.68 | -14920.3 | 66587.73 | -33290.9 | 36704.62 | -18349.3 |
| (1,1,1) | 29804.03 | -14898 | 66383.07 | -33187.5 | 36705.86 | -18348.9 |
| (1,1,2) | 29776.06 | -14883 | 66384.93 | -33187.5 | 36306.11 | -18148.1 |
| (1,1,3) | 29378.43 | -14683.2 | 66386.74 | -33187.4 | 36302.61 | -18145.3 |
| (1,1,4) | 29365.23 | -14675.6 | 66356.75 | -33171.4 | 36303.49 | -18144.7 |
| (2,1,0) | 29817.3 | -14904.6 | 66393.55 | -33192.8 | 36705.36 | -18348.7 |
| (2,1,1) | 29361.65 | -14675.8 | 66384.94 | -33187.5 | 36543.74 | -18266.9 |
| (2,1,2) | 29363.05 | -14675.5 | 66386.85 | -33187.4 | 36302.25 | -18145.1 |
| (2,1,3) | 29364.85 | -14675.4 | 66376.24 | -33181.1 | 36308.1 | -18147.1 |
| (2,1,4) | 29366.77 | -14675.4 | 66357.03 | -33170.5 | 36305.61 | -18144.8 |
| (3,1,0) | 29786.83 | -14888.4 | 66387.01 | -33188.5 | 36602.33 | -18296.2 |
| (3,1,1) | 29363.04 | -14675.5 | 66386.93 | -33187.5 | 36366.01 | -18177 |
| (3,1,2) | 29365.56 | -14675.8 | 66366.4 | -33176.2 | 36302.72 | -18144.4 |
| (3,1,3) | 29366.89 | -14675.4 | 66369.64 | -33176.8 | 36305.56 | -18144.8 |
| (3,1,4) | 29368.84 | -14675.4 | 66357.51 | -33169.8 | 36307.72 | -18144.9 |
| (4,1,0) | 29756.72 | -14872.4 | 66380.14 | -33184.1 | 36430.06 | -18209 |
| (4,1,1) | 29364.87 | -14675.4 | 66376.12 | -33181.1 | 36320.04 | -18153 |
| (4,1,2) | 29366.81 | -14675.4 | 66358.98 | -33171.5 | 36284.76 | -18134.4 |
| (4,1,3) | 29367.47 | -14674.7 | 66354.98 | -33168.5 | 36306.66 | -18144.3 |
| (4,1,4) | 29358.73 | -14669.4 | 66356.1 | -33168.1 | 36309.51 | -18144.8 |

1. Patient 12

| ARIMA Model | ICP ARIMA AIC | ICP ARIMA LL | MAP ARIMA AIC | MAP ARIMA LL | PbtO2 ARIMA AIC | PbtO2 ARIMA LL |
| --- | --- | --- | --- | --- | --- | --- |
| (0,1,0) | 80879.63 | -40437.8 | 85841.66 | -42918.8 | 4846.7 | -2421.35 |
| (0,1,1) | 80332.44 | -40163.2 | 85701.38 | -42847.7 | 1695.027 | -844.513 |
| (0,1,2) | 73065.46 | -36528.7 | 85370.87 | -42681.4 | 802.7674 | -397.384 |
| (0,1,3) | 72495.21 | -36242.6 | 85228.59 | -42609.3 | -50.2536 | 30.12678 |
| (0,1,4) | 72326.99 | -36157.5 | 85186.44 | -42587.2 | -344.012 | 178.0061 |
| (1,1,0) | 80746.74 | -40370.4 | 85739.66 | -42866.8 | 310.5318 | -152.266 |
| (1,1,1) | 79454.83 | -39723.4 | 85583.89 | -42787.9 | -650.074 | 329.0368 |
| (1,1,2) | 72691.9 | -36341 | 85138.18 | -42564.1 | -651.087 | 330.5437 |
| (1,1,3) | 72433.33 | -36210.7 | 85102.37 | -42545.2 | -723.131 | 367.5656 |
| (1,1,4) | 72023.32 | -36004.7 | 85048.7 | -42517.3 | -721.696 | 367.848 |
| (2,1,0) | 76357.78 | -38174.9 | 85343.84 | -42667.9 | -398.635 | 203.3177 |
| (2,1,1) | 72081.42 | -36035.7 | 85186 | -42588 | -650.321 | 330.1603 |
| (2,1,2) | 71961.87 | -35974.9 | 85126.72 | -42557.4 | -663.337 | 337.6686 |
| (2,1,3) | 71825.15 | -35905.6 | 85137.81 | -42561.9 | -722.655 | 368.3273 |
| (2,1,4) | 71823.6 | -35903.8 | 85072.87 | -42528.4 | -721.83 | 368.9149 |
| (3,1,0) | 75591.01 | -37790.5 | 85262.01 | -42626 | -719.986 | 364.9929 |
| (3,1,1) | 72014.07 | -36001 | 85070.68 | -42529.3 | -718.886 | 365.443 |
| (3,1,2) | 71891.83 | -35938.9 | 85038.99 | -42512.5 | -716.894 | 365.447 |
| (3,1,3) | 71825.12 | -35904.6 | 85040.44 | -42512.2 | -1073.96 | 544.9779 |
| (3,1,4) | 71829.04 | -35905.5 | 85041.41 | -42511.7 | -1064.28 | 541.1393 |
| (4,1,0) | 74224.49 | -37106.2 | 85222.11 | -42605.1 | -718.864 | 365.4318 |
| (4,1,1) | 71813.76 | -35899.9 | 85046.21 | -42516.1 | -716.564 | 365.2822 |
| (4,1,2) | 71815.08 | -35899.5 | 85071.64 | -42527.8 | -715.081 | 365.5403 |
| (4,1,3) | 71816.45 | -35899.2 | 85038.5 | -42510.3 | -715.807 | 366.9034 |
| (4,1,4) | 71819.88 | -35899.9 | 85040.72 | -42510.4 | -1081.31 | 550.657 |

1. Patient 13

| ARIMA Model | ICP ARIMA AIC | ICP ARIMA LL | MAP ARIMA AIC | MAP ARIMA LL | PbtO2 ARIMA AIC | PbtO2 ARIMA LL |
| --- | --- | --- | --- | --- | --- | --- |
| (0,1,0) | 16322.67 | -8159.33 | 39209.3 | -19602.7 | 30027.28 | -15011.6 |
| (0,1,1) | 14553.34 | -7273.67 | 38429.37 | -19211.7 | 25531.78 | -12762.9 |
| (0,1,2) | 14343.84 | -7167.92 | 38357.55 | -19174.8 | 25514.65 | -12753.3 |
| (0,1,3) | 14323.48 | -7156.74 | 38281.88 | -19135.9 | 25513.92 | -12752 |
| (0,1,4) | 14243.31 | -7115.65 | 38272.33 | -19130.2 | 25514.18 | -12751.1 |
| (1,1,0) | 15257.21 | -7625.61 | 38587.84 | -19290.9 | 27308.61 | -13651.3 |
| (1,1,1) | 14297.08 | -7144.54 | 38293.68 | -19142.8 | 25513.87 | -12752.9 |
| (1,1,2) | 14251.14 | -7120.57 | 38239.12 | -19114.6 | 25514.88 | -12752.4 |
| (1,1,3) | 14218.94 | -7103.47 | 38240.31 | -19114.2 | 25502.78 | -12745.4 |
| (1,1,4) | 14245.24 | -7115.62 | 38198.6 | -19092.3 | 25495.95 | -12741 |
| (2,1,0) | 14785.44 | -7388.72 | 38488.15 | -19240.1 | 26397.96 | -13195 |
| (2,1,1) | 14275 | -7132.5 | 38241.31 | -19115.7 | 25514.29 | -12752.1 |
| (2,1,2) | 14218.07 | -7103.03 | 38240.85 | -19114.4 | 25492.11 | -12740.1 |
| (2,1,3) | 14217.6 | -7101.8 | 38242.14 | -19114.1 | 25515.39 | -12750.7 |
| (2,1,4) | 14242.84 | -7113.42 | 38202.12 | -19093.1 | 25495.65 | -12739.8 |
| (3,1,0) | 14693.59 | -7341.79 | 38386.7 | -19188.3 | 25995.56 | -12992.8 |
| (3,1,1) | 14233.31 | -7110.66 | 38242.93 | -19115.5 | 25514.15 | -12751.1 |
| (3,1,2) | 14231.79 | -7108.89 | 38245.23 | -19115.6 | 25493.69 | -12739.8 |
| (3,1,3) | 14226.18 | -7105.09 | 38205.67 | -19094.8 | 25496.04 | -12740 |
| (3,1,4) | 14193.41 | -7087.71 | 38236.43 | -19109.2 | 25497.56 | -12739.8 |
| (4,1,0) | 14509.13 | -7248.56 | 38339.16 | -19163.6 | 25795.16 | -12891.6 |
| (4,1,1) | 14234.37 | -7110.18 | 38232.18 | -19109.1 | 25513.67 | -12749.8 |
| (4,1,2) | 14233.47 | -7108.74 | 38236.62 | -19110.3 | 25495.69 | -12739.8 |
| (4,1,3) | 14203.52 | -7092.76 | 38234.32 | -19108.2 | 25497.64 | -12739.8 |
| (4,1,4) | 14163.05 | -7071.53 | 38209.44 | -19094.7 | 25499.9 | -12739.9 |

1. Patient 14

| ARIMA Model | ICP ARIMA AIC | ICP ARIMA LL | MAP ARIMA AIC | MAP ARIMA LL | PbtO2 ARIMA AIC | PbtO2 ARIMA LL |
| --- | --- | --- | --- | --- | --- | --- |
| (0,1,0) | 119513.4 | -59754.7 | 145824.8 | -72910.4 | 17972.94 | -8984.47 |
| (0,1,1) | 118836.6 | -59415.3 | 142912.1 | -71453.1 | 14099.04 | -7046.52 |
| (0,1,2) | 116594.5 | -58293.3 | 142752.2 | -71372.1 | 12844.79 | -6418.39 |
| (0,1,3) | 116444.6 | -58217.3 | 142662.4 | -71326.2 | 12249.52 | -6119.76 |
| (0,1,4) | 116438.9 | -58213.4 | 142659.5 | -71323.7 | 11968.2 | -5978.1 |
| (1,1,0) | 119199 | -59596.5 | 143541.7 | -71767.8 | 12456.79 | -6225.4 |
| (1,1,1) | 116819.8 | -58405.9 | 142688.6 | -71340.3 | 11724.16 | -5858.08 |
| (1,1,2) | 116419.1 | -58204.6 | 142544.5 | -71267.2 | 11726.16 | -5858.08 |
| (1,1,3) | 116120.5 | -58054.2 | 142507.8 | -71247.9 | 11726.43 | -5857.21 |
| (1,1,4) | 116077.3 | -58031.6 | 142483.5 | -71234.8 | 11728.38 | -5857.19 |
| (2,1,0) | 117327.7 | -58659.8 | 143089.5 | -71540.7 | 11831.32 | -5911.66 |
| (2,1,1) | 116525 | -58257.5 | 142594 | -71292 | 11726.15 | -5858.07 |
| (2,1,2) | 116405.5 | -58196.7 | 142508 | -71248 | 11727.95 | -5857.97 |
| (2,1,3) | 116082.3 | -58034.2 | 142544.1 | -71265.1 | 11729.32 | -5857.66 |
| (2,1,4) | 116072.6 | -58028.3 | 142509.3 | -71246.7 | 11730.33 | -5857.17 |
| (3,1,0) | 116971.6 | -58480.8 | 142809.2 | -71399.6 | 11731.66 | -5860.83 |
| (3,1,1) | 116215.6 | -58101.8 | 142569.1 | -71278.5 | 11726.34 | -5857.17 |
| (3,1,2) | 116102.2 | -58044.1 | 142595.8 | -71290.9 | 11729.13 | -5857.56 |
| (3,1,3) | 116080.9 | -58032.4 | 142491.6 | -71237.8 | 11730.24 | -5857.12 |
| (3,1,4) | 116076.6 | -58029.3 | 142494.8 | -71238.4 | 11731.61 | -5856.81 |
| (4,1,0) | 116833.5 | -58410.8 | 142762.5 | -71375.2 | 11726.69 | -5857.35 |
| (4,1,1) | 116135.4 | -58060.7 | 142490.6 | -71238.3 | 11729.13 | -5857.57 |
| (4,1,2) | 116059.1 | -58021.6 | 142486 | -71235 | 11730.24 | -5857.12 |
| (4,1,3) | 116104.5 | -58043.2 | 142469.1 | -71225.6 | 11731.81 | -5856.9 |
| (4,1,4) | 116081.8 | -58030.9 | 142455.8 | -71217.9 | 11734.36 | -5857.18 |

1. Patient 15

| ARIMA Model | ICP ARIMA AIC | ICP ARIMA LL | MAP ARIMA AIC | MAP ARIMA LL | PbtO2 ARIMA AIC | PbtO2 ARIMA LL |
| --- | --- | --- | --- | --- | --- | --- |
| (0,1,0) | 141887.8 | -70941.9 | 165133.3 | -82564.7 | 33534.99 | -16765.5 |
| (0,1,1) | 139755.6 | -69874.8 | 160783.4 | -80388.7 | 19900.09 | -9947.05 |
| (0,1,2) | 135087.8 | -67539.9 | 160044.9 | -80018.4 | 18826.53 | -9409.26 |
| (0,1,3) | 135031.4 | -67510.7 | 159966.2 | -79978.1 | 18785.14 | -9387.57 |
| (0,1,4) | 134957.9 | -67473 | 159928.6 | -79958.3 | 18681.63 | -9334.82 |
| (1,1,0) | 141249.3 | -70621.6 | 162315 | -81154.5 | 21182.32 | -10588.2 |
| (1,1,1) | 136231.1 | -68111.5 | 159923.5 | -79957.7 | 18860.06 | -9426.03 |
| (1,1,2) | 135045 | -67517.5 | 159917.8 | -79953.9 | 18802.45 | -9396.23 |
| (1,1,3) | 135003 | -67495.5 | 159856 | -79922 | 18728.17 | -9358.09 |
| (1,1,4) | 134936.8 | -67461.4 | 159883.8 | -79934.9 | 18478.67 | -9232.33 |
| (2,1,0) | 137600.4 | -68796.2 | 161049.2 | -80520.6 | 18784.23 | -9388.12 |
| (2,1,1) | 134932.8 | -67461.4 | 159919.4 | -79954.7 | 18731.17 | -9360.59 |
| (2,1,2) | 134927.8 | -67457.9 | 159927.3 | -79957.6 | 18536.8 | -9262.4 |
| (2,1,3) | 134923.8 | -67454.9 | 159902 | -79944 | 18708.73 | -9347.37 |
| (2,1,4) | 134923.1 | -67453.5 | 159797.3 | -79890.7 | 18436.86 | -9210.43 |
| (3,1,0) | 136366.1 | -68178 | 160569.8 | -80279.9 | 18744.48 | -9367.24 |
| (3,1,1) | 134929.1 | -67458.5 | 159898.7 | -79943.4 | 18670.39 | -9329.2 |
| (3,1,2) | 134926 | -67456 | 159907.3 | -79946.7 | 18672.01 | -9329 |
| (3,1,3) | 134927.4 | -67455.7 | 159829.4 | -79906.7 | 18535.89 | -9259.94 |
| (3,1,4) | 134927.8 | -67454.9 | 159807.6 | -79894.8 | 18432.77 | -9207.39 |
| (4,1,0) | 135591 | -67789.5 | 160297.1 | -80142.5 | 18708.87 | -9348.44 |
| (4,1,1) | 134921.7 | -67453.9 | 159894.3 | -79940.2 | 18672.07 | -9329.04 |
| (4,1,2) | 134924.2 | -67454.1 | 159902.3 | -79943.1 | 18463.01 | -9223.51 |
| (4,1,3) | 134920.3 | -67451.2 | 159744.8 | -79863.4 | 18461.7 | -9221.85 |
| (4,1,4) | 134921.7 | -67450.9 | 159736.3 | -79858.1 | 18432.02 | -9206.01 |

1. Patient 16

| ARIMA Model | ICP ARIMA AIC | ICP ARIMA LL | MAP ARIMA AIC | MAP ARIMA LL | PbtO2 ARIMA AIC | PbtO2 ARIMA LL |
| --- | --- | --- | --- | --- | --- | --- |
| (0,1,0) | 35078.03 | -17537 | 75130.65 | -37563.3 | -7878.81 | 3941.403 |
| (0,1,1) | 33430.9 | -16712.4 | 70067.27 | -35030.6 | -8676.95 | 4341.476 |
| (0,1,2) | 31813.56 | -15902.8 | 69949.44 | -34970.7 | -9196.66 | 4602.332 |
| (0,1,3) | 31383.59 | -15686.8 | 69879.31 | -34934.7 | -9409.23 | 4709.616 |
| (0,1,4) | 31375.91 | -15682 | 69878.66 | -34933.3 | -9526.57 | 4769.286 |
| (1,1,0) | 34330.03 | -17162 | 71912.91 | -35953.5 | -8975.04 | 4490.518 |
| (1,1,1) | 31681.46 | -15836.7 | 69925.31 | -34958.7 | -9898.82 | 4953.41 |
| (1,1,2) | 31604.22 | -15797.1 | 69808.97 | -34899.5 | -9897.07 | 4953.535 |
| (1,1,3) | 31382.78 | -15685.4 | 69879.06 | -34933.5 | -9913.95 | 4962.974 |
| (1,1,4) | 31377.67 | -15681.8 | 69880.05 | -34933 | -9912.23 | 4963.115 |
| (2,1,0) | 33987.5 | -16989.7 | 71095.16 | -35543.6 | -9539.61 | 4773.805 |
| (2,1,1) | 31528.03 | -15759 | 69876.45 | -34933.2 | -9897.1 | 4953.55 |
| (2,1,2) | 31211.16 | -15599.6 | 69878.51 | -34933.3 | -9897.94 | 4954.969 |
| (2,1,3) | 30437.47 | -15211.7 | 69811.98 | -34899 | -9902.79 | 4958.393 |
| (2,1,4) | 30324.16 | -15154.1 | 69878.73 | -34931.4 | -9909.96 | 4962.982 |
| (3,1,0) | 32695.17 | -16342.6 | 70590.65 | -35290.3 | -9689.53 | 4849.764 |
| (3,1,1) | 30903.54 | -15445.8 | 69878.44 | -34933.2 | -9913.81 | 4962.904 |
| (3,1,2) | 30809.29 | -15397.6 | 69880.42 | -34933.2 | -9902.78 | 4958.389 |
| (3,1,3) | 30278.09 | -15131 | 69877.76 | -34930.9 | -9910.05 | 4963.023 |
| (3,1,4) | 30408.72 | -15195.4 | 69882.26 | -34932.1 | -9908.22 | 4963.109 |
| (4,1,0) | 31271.92 | -15630 | 70211.85 | -35099.9 | -9767.27 | 4889.636 |
| (4,1,1) | 30721.33 | -15353.7 | 69870.43 | -34928.2 | -9911.82 | 4962.909 |
| (4,1,2) | 30720.83 | -15352.4 | 69882.39 | -34933.2 | -9910.1 | 4963.05 |
| (4,1,3) | 30270.64 | -15126.3 | 69881.46 | -34931.7 | -9907.71 | 4962.857 |
| (4,1,4) | 30280.1 | -15130.1 | 69880.68 | -34930.3 | -9929.36 | 4974.681 |

1. Patient 17

| ARIMA Model | ICP ARIMA AIC | ICP ARIMA LL | MAP ARIMA AIC | MAP ARIMA LL | PbtO2 ARIMA AIC | PbtO2 ARIMA LL |
| --- | --- | --- | --- | --- | --- | --- |
| (0,1,0) | 150428.1 | -75212.1 | 156449.6 | -78222.8 | -5830.22 | 2917.108 |
| (0,1,1) | 149930.6 | -74962.3 | 151315.7 | -75654.8 | -21538.1 | 10772.05 |
| (0,1,2) | 147696.8 | -73844.4 | 151168.3 | -75580.2 | -29692 | 14849.98 |
| (0,1,3) | 146388.6 | -73189.3 | 151165 | -75577.5 | -33765.6 | 16887.8 |
| (0,1,4) | 146219.9 | -73104 | 151128 | -75558 | -35721.3 | 17866.67 |
| (1,1,0) | 150158.4 | -75076.2 | 152703.6 | -76348.8 | -35753.2 | 17879.58 |
| (1,1,1) | 149476.8 | -74734.4 | 151166.3 | -75579.2 | -36553.8 | 18280.9 |
| (1,1,2) | 146362.9 | -73176.5 | 151167.6 | -75578.8 | -37450.5 | 18730.25 |
| (1,1,3) | 146250.3 | -73119.2 | 151141.8 | -75564.9 | -37510.9 | 18761.45 |
| (1,1,4) | 146221.5 | -73103.8 | 151126.8 | -75556.4 | -37517 | 18765.48 |
| (2,1,0) | 147853.1 | -73922.5 | 151710.7 | -75851.4 | -36845.5 | 18426.76 |
| (2,1,1) | 146218 | -73104 | 151167.1 | -75578.6 | -37046.7 | 18528.33 |
| (2,1,2) | 146213.9 | -73101 | 151160.1 | -75574.1 | -37522.8 | 18767.38 |
| (2,1,3) | 146215.5 | -73100.7 | 151108.2 | -75547.1 | -37492.7 | 18753.35 |
| (2,1,4) | 146194.4 | -73089.2 | 151106.1 | -75545 | -37511.4 | 18763.69 |
| (3,1,0) | 146998.9 | -73494.4 | 151184.4 | -75587.2 | -37254.8 | 18632.38 |
| (3,1,1) | 146213.1 | -73100.5 | 151135.9 | -75562 | -37573.8 | 18792.88 |
| (3,1,2) | 146214.2 | -73100.1 | 151130.9 | -75558.4 | -37573.6 | 18793.82 |
| (3,1,3) | 146216.1 | -73100 | 151105.5 | -75544.7 | -37520.2 | 18768.1 |
| (3,1,4) | 145627.2 | -72804.6 | 151096.3 | -75539.1 | -37649.7 | 18833.83 |
| (4,1,0) | 146455.3 | -73221.7 | 151155.8 | -75571.9 | -37534.5 | 18773.26 |
| (4,1,1) | 145938.3 | -72962.2 | 151127 | -75556.5 | -37575.4 | 18794.69 |
| (4,1,2) | 146216.1 | -73100 | 151128 | -75556 | -37572.3 | 18794.13 |
| (4,1,3) | 146217.7 | -73099.9 | 151092.6 | -75537.3 | -37573.9 | 18795.96 |
| (4,1,4) | 145641.1 | -72810.5 | 151081.7 | -75530.9 | -37515.9 | 18767.96 |

1. Patient 18

| ARIMA Model | ICP ARIMA AIC | ICP ARIMA LL | MAP ARIMA AIC | MAP ARIMA LL | PbtO2 ARIMA AIC | PbtO2 ARIMA LL |
| --- | --- | --- | --- | --- | --- | --- |
| (0,1,0) | 123961 | -61978.5 | 124285.2 | -62140.6 | -16658 | 8330.989 |
| (0,1,1) | 123730.9 | -61862.4 | 116177.1 | -58085.6 | -31089.2 | 15547.59 |
| (0,1,2) | 120027.8 | -60009.9 | 115957.5 | -57974.8 | -36501.1 | 18254.54 |
| (0,1,3) | 119768 | -59879 | 115941.7 | -57965.8 | -38486.1 | 19248.05 |
| (0,1,4) | 119769.5 | -59878.8 | 115936.6 | -57962.3 | -39374.3 | 19693.17 |
| (1,1,0) | 123881 | -61937.5 | 119998.9 | -59996.5 | -39676.3 | 19841.15 |
| (1,1,1) | 120971 | -60481.5 | 115978.9 | -57985.4 | -39721.1 | 19864.54 |
| (1,1,2) | 119785.6 | -59887.8 | 115947 | -57968.5 | -39858.2 | 19934.08 |
| (1,1,3) | 119769.5 | -59878.7 | 115942.6 | -57965.3 | -39892.3 | 19952.17 |
| (1,1,4) | 119771.4 | -59878.7 | 115936.7 | -57961.4 | -39930.7 | 19972.36 |
| (2,1,0) | 121207.9 | -60600 | 117767.8 | -58879.9 | -39730.8 | 19869.4 |
| (2,1,1) | 119891.7 | -59940.8 | 115936.5 | -57963.3 | -40087.1 | 20048.55 |
| (2,1,2) | 119775.5 | -59881.8 | 115938 | -57963 | -40086.2 | 20049.12 |
| (2,1,3) | 119770.8 | -59878.4 | 115936.2 | -57961.1 | -40145.5 | 20079.75 |
| (2,1,4) | 119772.2 | -59878.1 | 115936.2 | -57960.1 | -40152.9 | 20084.46 |
| (3,1,0) | 120681 | -60335.5 | 116737.6 | -58363.8 | -39898.2 | 19954.12 |
| (3,1,1) | 119753.4 | -59870.7 | 115937.3 | -57962.7 | -40086.4 | 20049.2 |
| (3,1,2) | 119689.9 | -59837.9 | 115938.1 | -57962 | -40086.6 | 20050.29 |
| (3,1,3) | 119771 | -59877.5 | 115936.2 | -57960.1 | -40083.2 | 20049.58 |
| (3,1,4) | 119534.2 | -59758.1 | 115937.8 | -57959.9 | -40142.1 | 20080.03 |
| (4,1,0) | 120300.9 | -60144.5 | 116361.7 | -58174.9 | -39940.9 | 19976.43 |
| (4,1,1) | 119751.6 | -59868.8 | 115937.9 | -57962 | -40152.7 | 20083.36 |
| (4,1,2) | 119757.3 | -59870.7 | 115938.7 | -57961.4 | -40083.5 | 20049.76 |
| (4,1,3) | 119584.9 | -59783.5 | 115940.4 | -57961.2 | -40093.1 | 20055.57 |
| (4,1,4) | 119526.6 | -59753.3 | 115929.5 | -57954.8 | -40094 | 20056.99 |

1. Patient 19

| ARIMA Model | ICP ARIMA AIC | ICP ARIMA LL | MAP ARIMA AIC | MAP ARIMA LL | PbtO2 ARIMA AIC | PbtO2 ARIMA LL |
| --- | --- | --- | --- | --- | --- | --- |
| (0,1,0) | 292014.5 | -146005 | 357509 | -178753 | 167413.1 | -83704.5 |
| (0,1,1) | 290086 | -145040 | 347012.6 | -173503 | 156803.2 | -78398.6 |
| (0,1,2) | 285968.3 | -142980 | 346441 | -173216 | 152203.6 | -76097.8 |
| (0,1,3) | 285441.3 | -142716 | 346433.3 | -173212 | 150713.9 | -75352 |
| (0,1,4) | 284986.5 | -142487 | 346433.2 | -173211 | 150125.1 | -75056.5 |
| (1,1,0) | 291001.2 | -145498 | 349829.2 | -174912 | 152235.8 | -76114.9 |
| (1,1,1) | 288361.6 | -144177 | 346432.2 | -173212 | 150008.5 | -75000.3 |
| (1,1,2) | 285664.3 | -142827 | 346432.9 | -173211 | 149835.3 | -74912.7 |
| (1,1,3) | 285074.1 | -142531 | 346425 | -173207 | 149835.8 | -74911.9 |
| (1,1,4) | 284977.9 | -142482 | 346421 | -173204 | 149833.2 | -74909.6 |
| (2,1,0) | 285168.6 | -142580 | 347341.4 | -173667 | 149884.7 | -74938.3 |
| (2,1,1) | 285169.9 | -142580 | 346433.3 | -173212 | 149844.4 | -74917.2 |
| (2,1,2) | 285169.3 | -142579 | 346435.4 | -173212 | 149834.9 | -74911.5 |
| (2,1,3) | 284994 | -142490 | 346335.4 | -173161 | 149839.3 | -74912.6 |
| (2,1,4) | 284969.2 | -142477 | 346305.4 | -173145 | 149836.9 | -74910.4 |
| (3,1,0) | 285169.9 | -142580 | 346538.1 | -173264 | 149840.9 | -74915.4 |
| (3,1,1) | 285172.1 | -142580 | 345942.8 | -172965 | 149842.6 | -74915.3 |
| (3,1,2) | 285083.1 | -142535 | 346437.1 | -173212 | 149844.7 | -74915.3 |
| (3,1,3) | 284992.1 | -142488 | 346284.8 | -173134 | 149833.1 | -74908.5 |
| (3,1,4) | 284971 | -142476 | 345402.2 | -172692 | 149840 | -74911 |
| (4,1,0) | 285169.2 | -142579 | 346520.7 | -173254 | 149841.4 | -74914.7 |
| (4,1,1) | 285100.3 | -142543 | 345504.6 | -172745 | 149844.3 | -74915.2 |
| (4,1,2) | 284998.1 | -142491 | 345504.2 | -172744 | 149843.6 | -74913.8 |
| (4,1,3) | 284920.8 | -142451 | 345502.7 | -172742 | 149842.4 | -74912.2 |
| (4,1,4) | 284919.7 | -142450 | 345425.5 | -172703 | 149322.7 | -74651.3 |

1. Patient 20

| ARIMA Model | ICP ARIMA AIC | ICP ARIMA LL | MAP ARIMA AIC | MAP ARIMA LL | PbtO2 ARIMA AIC | PbtO2 ARIMA LL |
| --- | --- | --- | --- | --- | --- | --- |
| (0,1,0) | 48513.76 | -24254.9 | 85705.55 | -42850.8 | -1552.81 | 778.4062 |
| (0,1,1) | 46902.03 | -23448 | 77725.92 | -38860 | -6009.39 | 3007.694 |
| (0,1,2) | 46350.25 | -23171.1 | 77703.97 | -38848 | -7698.84 | 3853.419 |
| (0,1,3) | 46322.95 | -23156.5 | 77702.96 | -38846.5 | -8614.94 | 4312.468 |
| (0,1,4) | 46310.84 | -23149.4 | 77691.37 | -38839.7 | -8844.84 | 4428.422 |
| (1,1,0) | 47546.08 | -23770 | 80670.4 | -40332.2 | -8579.83 | 4292.916 |
| (1,1,1) | 46491.71 | -23241.9 | 77705.01 | -38848.5 | -10048.3 | 5028.14 |
| (1,1,2) | 46319.62 | -23154.8 | 77704.87 | -38847.4 | -10049.2 | 5029.593 |
| (1,1,3) | 46321.35 | -23154.7 | 77701.2 | -38844.6 | -10055 | 5033.477 |
| (1,1,4) | 46066.33 | -23026.2 | 77693.37 | -38839.7 | -10090.2 | 5052.117 |
| (2,1,0) | 46425.48 | -23208.7 | 78760.92 | -39376.5 | -9644.85 | 4826.425 |
| (2,1,1) | 46367.42 | -23178.7 | 77701.04 | -38845.5 | -10049 | 5029.482 |
| (2,1,2) | 46320.87 | -23154.4 | 77699.1 | -38843.5 | -10057.9 | 5034.945 |
| (2,1,3) | 46264.88 | -23125.4 | 77676.79 | -38831.4 | -10056.4 | 5035.205 |
| (2,1,4) | 46243.37 | -23113.7 | 77678.77 | -38831.4 | -10055.3 | 5035.647 |
| (3,1,0) | 46396.6 | -23193.3 | 78195.39 | -39092.7 | -9929.27 | 4969.635 |
| (3,1,1) | 46067.47 | -23027.7 | 77690.49 | -38839.2 | -10052.6 | 5032.307 |
| (3,1,2) | 46066.57 | -23026.3 | 77692.27 | -38839.1 | -10056.4 | 5035.204 |
| (3,1,3) | 46230.49 | -23107.2 | 77678.77 | -38831.4 | -10054.4 | 5035.213 |
| (3,1,4) | 45996.55 | -22989.3 | 77680.81 | -38831.4 | -10087.6 | 5052.807 |
| (4,1,0) | 46346.89 | -23167.4 | 78001.29 | -38994.6 | -9999.35 | 5005.677 |
| (4,1,1) | 46067.35 | -23026.7 | 77691.8 | -38838.9 | -10061.6 | 5037.812 |
| (4,1,2) | 46065.68 | -23024.8 | 77685.63 | -38834.8 | -10054.7 | 5035.326 |
| (4,1,3) | 46067.68 | -23024.8 | 77677.5 | -38829.8 | -10091.1 | 5054.53 |
| (4,1,4) | 45995.33 | -22987.7 | 77672.06 | -38826 | -10107.2 | 5063.612 |

1. Patient 21

| ARIMA Model | ICP ARIMA AIC | ICP ARIMA LL | MAP ARIMA AIC | MAP ARIMA LL | PbtO2 ARIMA AIC | PbtO2 ARIMA LL |
| --- | --- | --- | --- | --- | --- | --- |
| (0,1,0) | 73030.74 | -36513.4 | 111073.2 | -55534.6 | 1696.795 | -846.398 |
| (0,1,1) | 72795.3 | -36394.7 | 111042.5 | -55518.2 | -11834.6 | 5920.299 |
| (0,1,2) | 67265.57 | -33628.8 | 109841.4 | -54916.7 | -18281.1 | 9144.557 |
| (0,1,3) | 67245.37 | -33617.7 | 109353.2 | -54671.6 | -20673.1 | 10341.54 |
| (0,1,4) | 67109.78 | -33548.9 | 109323.4 | -54655.7 | -22108.4 | 11060.22 |
| (1,1,0) | 72989.5 | -36491.7 | 111055.4 | -55524.7 | -22747.2 | 11376.62 |
| (1,1,1) | 69971.78 | -34981.9 | 110811.3 | -55401.6 | -23062.3 | 11535.17 |
| (1,1,2) | 67251.19 | -33620.6 | 109211.7 | -54600.8 | -23295.8 | 11652.9 |
| (1,1,3) | 67192.25 | -33590.1 | 109204.8 | -54596.4 | -23294.3 | 11653.17 |
| (1,1,4) | 67111.62 | -33548.8 | 109128.1 | -54557 | -23311.9 | 11662.94 |
| (2,1,0) | 68038.76 | -34015.4 | 109993 | -54992.5 | -23133.8 | 11570.9 |
| (2,1,1) | 67480.89 | -33735.4 | 109299 | -54644.5 | -23251.5 | 11630.75 |
| (2,1,2) | 67113.58 | -33550.8 | 109209 | -54598.5 | -23295.1 | 11653.57 |
| (2,1,3) | 67106.29 | -33546.1 | 109215.4 | -54600.7 | -23295.1 | 11654.53 |
| (2,1,4) | 67100.75 | -33542.4 | 109131.6 | -54557.8 | -23309.8 | 11662.91 |
| (3,1,0) | 67834.79 | -33912.4 | 109815.3 | -54902.7 | -23296 | 11652.99 |
| (3,1,1) | 67182.64 | -33585.3 | 109178.8 | -54583.4 | -23330.9 | 11671.45 |
| (3,1,2) | 67109.49 | -33547.7 | 109166 | -54576 | -23363 | 11688.5 |
| (3,1,3) | 67108.12 | -33546.1 | 109025.9 | -54505 | -23343.5 | 11679.74 |
| (3,1,4) | 67099.55 | -33540.8 | 108854.5 | -54418.2 | -23308.6 | 11663.32 |
| (4,1,0) | 67361.77 | -33674.9 | 109662.7 | -54825.3 | -23304.2 | 11658.1 |
| (4,1,1) | 67095.25 | -33540.6 | 109180.4 | -54583.2 | -23293.7 | 11653.84 |
| (4,1,2) | 67096.38 | -33540.2 | 109180.6 | -54582.3 | -23358.4 | 11687.2 |
| (4,1,3) | 67090.6 | -33536.3 | 108852.4 | -54417.2 | -23359.2 | 11688.62 |
| (4,1,4) | 67086.45 | -33533.2 | 108848.4 | -54414.2 | -23308.1 | 11664.04 |

1. Patient 22

| ARIMA Model | ICP ARIMA AIC | ICP ARIMA LL | MAP ARIMA AIC | MAP ARIMA LL | PbtO2 ARIMA AIC | PbtO2 ARIMA LL |
| --- | --- | --- | --- | --- | --- | --- |
| (0,1,0) | 138557.3 | -69276.6 | 208388.5 | -104192 | 41410 | -20703 |
| (0,1,1) | 130175.1 | -65084.6 | 206299.2 | -103147 | 36686.21 | -18340.1 |
| (0,1,2) | 127518.1 | -63755 | 194623.4 | -97307.7 | 35351.05 | -17671.5 |
| (0,1,3) | 127077.6 | -63533.8 | 194604.2 | -97297.1 | 35165.56 | -17577.8 |
| (0,1,4) | 127061.1 | -63524.6 | 193910.4 | -96949.2 | 35119.29 | -17553.6 |
| (1,1,0) | 135863.6 | -67928.8 | 208261.6 | -104128 | 39023.44 | -19508.7 |
| (1,1,1) | 128551.2 | -64271.6 | 199343.9 | -99667.9 | 35790.46 | -17891.2 |
| (1,1,2) | 127178.2 | -63584.1 | 194610.1 | -97300.1 | 35216.74 | -17603.4 |
| (1,1,3) | 127071.3 | -63529.6 | 194309.2 | -97148.6 | 34672.73 | -17330.4 |
| (1,1,4) | 126957.7 | -63471.9 | 193837.6 | -96911.8 | 34667.86 | -17326.9 |
| (2,1,0) | 129084.2 | -64538.1 | 200689.1 | -100341 | 35865.22 | -17928.6 |
| (2,1,1) | 127348.8 | -63669.4 | 195342.4 | -97666.2 | 35160.12 | -17575.1 |
| (2,1,2) | 126997.1 | -63492.6 | 194308 | -97148 | 35154.95 | -17571.5 |
| (2,1,3) | 126653.1 | -63319.5 | 193952.9 | -96969.4 | 34665.4 | -17325.7 |
| (2,1,4) | 126109.6 | -63046.8 | 193693.8 | -96838.9 | 34585.42 | -17284.7 |
| (3,1,0) | 128555.5 | -64272.8 | 200619.3 | -100305 | 35406.12 | -17698.1 |
| (3,1,1) | 126933.2 | -63460.6 | 195055.1 | -97521.5 | 35158.95 | -17573.5 |
| (3,1,2) | 126887.5 | -63436.7 | 194309.9 | -97148 | 35147.5 | -17566.7 |
| (3,1,3) | 126380 | -63182 | 193821.7 | -96902.9 | 34714.64 | -17349.3 |
| (3,1,4) | 125998.6 | -62990.3 | 193210.8 | -96596.4 | 34587.14 | -17284.6 |
| (4,1,0) | 128163.2 | -64075.6 | 197478.7 | -98733.3 | 35161.17 | -17574.6 |
| (4,1,1) | 126799.9 | -63393 | 191793.5 | -95889.8 | 35124.78 | -17555.4 |
| (4,1,2) | 126509.1 | -63246.6 | 190657.7 | -95320.8 | 34686.93 | -17335.5 |
| (4,1,3) | 126109.3 | -63045.7 | 189896.5 | -94939.3 | 34655.33 | -17318.7 |
| (4,1,4) | 126256.3 | -63118.1 | 189847.1 | -94913.6 | 34630.24 | -17305.1 |

1. Patient 23

| ARIMA Model | ICP ARIMA AIC | ICP ARIMA LL | MAP ARIMA AIC | MAP ARIMA LL | PbtO2 ARIMA AIC | PbtO2 ARIMA LL |
| --- | --- | --- | --- | --- | --- | --- |
| (0,1,0) | 453494.7 | -226745 | 507010.6 | -253503 | 9941.65 | -4968.82 |
| (0,1,1) | 442411.9 | -221203 | 504958 | -252476 | 8897.972 | -4445.99 |
| (0,1,2) | 431313 | -215652 | 490881.9 | -245437 | 8547.768 | -4269.88 |
| (0,1,3) | 430547.5 | -215269 | 489489.6 | -244740 | 8539.004 | -4264.5 |
| (0,1,4) | 430384.9 | -215186 | 488774.3 | -244381 | 8373.895 | -4180.95 |
| (1,1,0) | 450143.1 | -225069 | 506019.6 | -253007 | 9031.007 | -4512.5 |
| (1,1,1) | 436361.7 | -218177 | 491596.9 | -245794 | 8611.009 | -4301.5 |
| (1,1,2) | 430723.1 | -215357 | 489054.8 | -244522 | 8545.035 | -4267.52 |
| (1,1,3) | 430460.5 | -215224 | 489016.7 | -244502 | 8135.248 | -4061.62 |
| (1,1,4) | 430026.7 | -215006 | 488719.3 | -244353 | 8004.273 | -3995.14 |
| (2,1,0) | 433553.5 | -216773 | 499420.5 | -249706 | 8566.452 | -4279.23 |
| (2,1,1) | 431505 | -215748 | 489348.7 | -244669 | 8529.01 | -4259.5 |
| (2,1,2) | 430317.6 | -215153 | 488965.6 | -244477 | 8499.993 | -4244 |
| (2,1,3) | 429853.4 | -214920 | 488961.7 | -244474 | 7987.921 | -3986.96 |
| (2,1,4) | 428257.9 | -214121 | 488158.1 | -244071 | 7995.419 | -3989.71 |
| (3,1,0) | 432870 | -216430 | 498580.9 | -249285 | 8492.442 | -4241.22 |
| (3,1,1) | 430624.2 | -215306 | 489304.9 | -244646 | 8078.8 | -4033.4 |
| (3,1,2) | 430249.4 | -215118 | 488946.1 | -244466 | 8007.532 | -3996.77 |
| (3,1,3) | 429811 | -214897 | 488569.3 | -244277 | 7989.773 | -3986.89 |
| (3,1,4) | 429856.9 | -214919 | 487930.7 | -243956 | 7985.664 | -3983.83 |
| (4,1,0) | 430772.9 | -215380 | 494192.2 | -247090 | 8364.903 | -4176.45 |
| (4,1,1) | 430088.3 | -215037 | 487655.6 | -243821 | 8033.52 | -4009.76 |
| (4,1,2) | 430089.5 | -215037 | 487643.3 | -243814 | 8060.582 | -4022.29 |
| (4,1,3) | 428027.5 | -214005 | 485246.5 | -242614 | 8011.284 | -3996.64 |
| (4,1,4) | 427697.8 | -213839 | 485044 | -242512 | 7985.882 | -3982.94 |

1. Patient 24

| ARIMA Model | ICP ARIMA AIC | ICP ARIMA LL | MAP ARIMA AIC | MAP ARIMA LL | PbtO2 ARIMA AIC | PbtO2 ARIMA LL |
| --- | --- | --- | --- | --- | --- | --- |
| (0,1,0) | 82732.53 | -41364.3 | 200184.2 | -100090 | 45765.99 | -22881 |
| (0,1,1) | 79995.99 | -39995 | 192732.3 | -96363.2 | 44541.9 | -22268 |
| (0,1,2) | 77829.55 | -38910.8 | 192382.2 | -96187.1 | 44543.49 | -22267.7 |
| (0,1,3) | 77801.25 | -38895.6 | 192099.5 | -96044.7 | 44545.05 | -22267.5 |
| (0,1,4) | 77776.44 | -38882.2 | 191989.3 | -95988.6 | 44546.05 | -22267 |
| (1,1,0) | 81202.08 | -40598 | 195481.2 | -97737.6 | 44576.97 | -22285.5 |
| (1,1,1) | 78060.25 | -39026.1 | 192480.4 | -96236.2 | 44543.52 | -22267.8 |
| (1,1,2) | 77794.05 | -38892 | 192041.8 | -96015.9 | 44545.6 | -22267.8 |
| (1,1,3) | 77492.75 | -38740.4 | 192007.8 | -95997.9 | 44546.9 | -22267.5 |
| (1,1,4) | 77767.8 | -38876.9 | 191985.5 | -95985.8 | 44546.81 | -22266.4 |
| (2,1,0) | 78933.83 | -39462.9 | 192327.5 | -96159.7 | 44542.44 | -22267.2 |
| (2,1,1) | 77829.84 | -38909.9 | 192175.9 | -96083 | 44544.94 | -22267.5 |
| (2,1,2) | 77772.31 | -38880.2 | 191998.1 | -95993 | 44546.87 | -22267.4 |
| (2,1,3) | 77770.99 | -38878.5 | 191936.3 | -95961.1 | 44548.9 | -22267.4 |
| (2,1,4) | 77767.71 | -38875.9 | 191273.4 | -95628.7 | 43786.84 | -21885.4 |
| (3,1,0) | 78411.33 | -39200.7 | 192265.1 | -96127.5 | 44544.28 | -22267.1 |
| (3,1,1) | 77791.02 | -38889.5 | 192051.4 | -96019.7 | 44546.42 | -22267.2 |
| (3,1,2) | 77770.85 | -38878.4 | 191969.7 | -95977.8 | 44547.96 | -22267 |
| (3,1,3) | 77773.27 | -38878.6 | 191359.2 | -95671.6 | 43859.61 | -21921.8 |
| (3,1,4) | 77770.21 | -38876.1 | 191337.5 | -95659.8 | 43787.89 | -21884.9 |
| (4,1,0) | 77950.71 | -38969.4 | 191986.7 | -95987.4 | 44541.29 | -22264.6 |
| (4,1,1) | 77692.03 | -38839 | 191945.7 | -95965.9 | 44541.74 | -22263.9 |
| (4,1,2) | 77772.79 | -38878.4 | 191947.4 | -95965.7 | 43706.96 | -21845.5 |
| (4,1,3) | 77774.64 | -38878.3 | 191949.3 | -95965.7 | 43699.24 | -21840.6 |
| (4,1,4) | 77767.46 | -38873.7 | 191103.1 | -95541.6 | 42971.89 | -21475.9 |

1. Patient 25

| ARIMA Model | ICP ARIMA AIC | ICP ARIMA LL | MAP ARIMA AIC | MAP ARIMA LL | PbtO2 ARIMA AIC | PbtO2 ARIMA LL |
| --- | --- | --- | --- | --- | --- | --- |
| (0,1,0) | 177713.4 | -88854.7 | 199424.5 | -99710.2 | 6499.237 | -3247.62 |
| (0,1,1) | 177436.2 | -88715.1 | 197205.6 | -98599.8 | 5127.799 | -2560.9 |
| (0,1,2) | 176858.2 | -88425.1 | 194407.9 | -97199.9 | 2250.511 | -1121.26 |
| (0,1,3) | 176662.6 | -88326.3 | 194397.1 | -97193.5 | 740.9777 | -365.489 |
| (0,1,4) | 176635.9 | -88312 | 193834.4 | -96911.2 | 193.734 | -90.867 |
| (1,1,0) | 177492.3 | -88743.1 | 198142.5 | -99068.2 | 4409.782 | -2201.89 |
| (1,1,1) | 177224 | -88608 | 194914.6 | -97453.3 | 608.2727 | -300.136 |
| (1,1,2) | 176624.6 | -88307.3 | 194384.6 | -97187.3 | -385.727 | 197.8635 |
| (1,1,3) | 176623.9 | -88306 | 194373.3 | -97180.6 | -384.95 | 198.4748 |
| (1,1,4) | 176623.8 | -88304.9 | 193512.3 | -96749.1 | -425.672 | 219.8359 |
| (2,1,0) | 176818.7 | -88405.3 | 194943.7 | -97467.8 | 713.0574 | -352.529 |
| (2,1,1) | 176622.1 | -88306.1 | 194786.7 | -97388.4 | -331.122 | 170.5612 |
| (2,1,2) | 176622.5 | -88305.2 | 194204 | -97096 | -384.51 | 198.255 |
| (2,1,3) | 176585.4 | -88285.7 | 193694.6 | -96840.3 | -383.878 | 198.9392 |
| (2,1,4) | 176581.9 | -88282.9 | 193359 | -96671.5 | -426.581 | 221.2906 |
| (3,1,0) | 176708.3 | -88349.2 | 194870.4 | -97430.2 | -352.088 | 181.0438 |
| (3,1,1) | 176623.3 | -88305.7 | 194785.4 | -97386.7 | -403.501 | 207.7506 |
| (3,1,2) | 176622.8 | -88304.4 | 193367.9 | -96676.9 | -401.947 | 207.9734 |
| (3,1,3) | 175878.6 | -87931.3 | 190115.4 | -95049.7 | -416.396 | 216.1981 |
| (3,1,4) | 176579.8 | -88280.9 | 189984.9 | -94983.5 | -388.428 | 203.2139 |
| (4,1,0) | 176612.1 | -88300.1 | 194098.3 | -97043.2 | -405.351 | 208.6756 |
| (4,1,1) | 176596.9 | -88291.4 | 193057 | -96521.5 | -403.252 | 208.6261 |
| (4,1,2) | 176562.7 | -88273.3 | 193053.2 | -96518.6 | -401.576 | 208.788 |
| (4,1,3) | 175649 | -87815.5 | 190007.6 | -94994.8 | -401.712 | 209.8561 |
| (4,1,4) | 175613.8 | -87796.9 | 190064.7 | -95022.3 | -430.791 | 225.3956 |

1. Patient 26

| ARIMA Model | ICP ARIMA AIC | ICP ARIMA LL | MAP ARIMA AIC | MAP ARIMA LL | PbtO2 ARIMA AIC | PbtO2 ARIMA LL |
| --- | --- | --- | --- | --- | --- | --- |
| (0,1,0) | 66209.49 | -33102.7 | 201272.7 | -100634 | 38913.82 | -19454.9 |
| (0,1,1) | 62807.73 | -31400.9 | 201262.6 | -100628 | 35204.86 | -17599.4 |
| (0,1,2) | 62288.22 | -31140.1 | 197662.3 | -98827.2 | 34449.46 | -17220.7 |
| (0,1,3) | 62286.25 | -31138.1 | 196861.1 | -98425.6 | 34331.22 | -17160.6 |
| (0,1,4) | 62288.23 | -31138.1 | 196827.8 | -98407.9 | 34262.04 | -17125 |
| (1,1,0) | 63781.81 | -31887.9 | 201268.6 | -100631 | 34471.82 | -17232.9 |
| (1,1,1) | 62310.46 | -31151.2 | 198855.8 | -99423.9 | 34409.54 | -17200.8 |
| (1,1,2) | 62286.23 | -31138.1 | 197168.6 | -98579.3 | 34370.68 | -17180.3 |
| (1,1,3) | 62288.34 | -31138.2 | 196852.1 | -98420 | 34259.84 | -17123.9 |
| (1,1,4) | 62290.25 | -31138.1 | 196337.3 | -98161.6 | 34237.78 | -17111.9 |
| (2,1,0) | 62670.94 | -31331.5 | 198830.1 | -99411.1 | 34400.24 | -17196.1 |
| (2,1,1) | 62286.64 | -31138.3 | 196406.3 | -98198.2 | 34395.53 | -17192.8 |
| (2,1,2) | 62288.62 | -31138.3 | 195260.7 | -97624.3 | 34364.75 | -17176.4 |
| (2,1,3) | 62290.21 | -31138.1 | 195247.1 | -97616.6 | 34183.02 | -17084.5 |
| (2,1,4) | 62292.39 | -31138.2 | 193835.1 | -96909.6 | 33906.91 | -16945.5 |
| (3,1,0) | 62405.56 | -31197.8 | 196695 | -98342.5 | 34385.8 | -17187.9 |
| (3,1,1) | 62288.33 | -31138.2 | 195905.5 | -97946.8 | 34386.59 | -17187.3 |
| (3,1,2) | 62290.34 | -31138.2 | 195251.6 | -97618.8 | 34169.26 | -17077.6 |
| (3,1,3) | 62292.05 | -31138 | 195260.6 | -97622.3 | 34026.85 | -17005.4 |
| (3,1,4) | 62294.04 | -31138 | 193727.7 | -96854.9 | 34081.59 | -17031.8 |
| (4,1,0) | 62324.11 | -31156.1 | 195721.5 | -97854.8 | 34377.13 | -17182.6 |
| (4,1,1) | 62289.44 | -31137.7 | 195668.9 | -97827.4 | 34312.77 | -17149.4 |
| (4,1,2) | 62292.32 | -31138.2 | 194387.4 | -97185.7 | 34026.05 | -17005 |
| (4,1,3) | 62293.97 | -31138 | 193587.8 | -96784.9 | 34033.15 | -17007.6 |
| (4,1,4) | 62294.33 | -31137.2 | 193955.8 | -96967.9 | 34025.87 | -17002.9 |

1. Patient 27

| ARIMA Model | ICP ARIMA AIC | ICP ARIMA LL | MAP ARIMA AIC | MAP ARIMA LL | PbtO2 ARIMA AIC | PbtO2 ARIMA LL |
| --- | --- | --- | --- | --- | --- | --- |
| (0,1,0) | 183048.3 | -91522.2 | 244718.2 | -122357 | 59083.74 | -29539.9 |
| (0,1,1) | 178987.2 | -89490.6 | 229235.3 | -114615 | 29419.37 | -14706.7 |
| (0,1,2) | 168723.2 | -84357.6 | 223650.1 | -111821 | 19114.31 | -9553.15 |
| (0,1,3) | 167550.5 | -83770.3 | 220487.6 | -110239 | 15875.22 | -7932.61 |
| (0,1,4) | 166967.9 | -83477.9 | 218870.1 | -109429 | 14675.8 | -7331.9 |
| (1,1,0) | 181553.7 | -90773.8 | 243262.6 | -121628 | 15507.36 | -7750.68 |
| (1,1,1) | 171858.5 | -85925.3 | 227810.8 | -113901 | 14480.59 | -7236.29 |
| (1,1,2) | 168148.6 | -84069.3 | 222632.7 | -111311 | 14142.87 | -7066.43 |
| (1,1,3) | 167201.7 | -83594.8 | 219857.5 | -109923 | 14082.63 | -7035.32 |
| (1,1,4) | 166968.1 | -83477.1 | 216373.9 | -108180 | 13951.7 | -6968.85 |
| (2,1,0) | 169186.5 | -84589.2 | 221136.2 | -110564 | 14250.09 | -7121.05 |
| (2,1,1) | 166178.7 | -83084.4 | 215669 | -107829 | 13752.45 | -6871.22 |
| (2,1,2) | 166175.2 | -83081.6 | 215646.4 | -107817 | 13748.25 | -6868.13 |
| (2,1,3) | 162875.3 | -81430.6 | 209772.8 | -104879 | 13749.96 | -6867.98 |
| (2,1,4) | 160040.2 | -80012.1 | 207045.8 | -103515 | 13729.32 | -6856.66 |
| (3,1,0) | 166262.8 | -83126.4 | 215232.8 | -107611 | 13946.07 | -6968.04 |
| (3,1,1) | 166141.8 | -83064.9 | 215227.1 | -107608 | 13748.2 | -6868.1 |
| (3,1,2) | 166132.4 | -83059.2 | 214637 | -107311 | 13756.25 | -6871.13 |
| (3,1,3) | 160773.5 | -80378.8 | 207732.7 | -103858 | 13751.35 | -6867.68 |
| (3,1,4) | 159910.3 | -79946.1 | 206955.4 | -103469 | 13752.62 | -6867.31 |
| (4,1,0) | 166119.4 | -83053.7 | 215225.8 | -107607 | 13860.84 | -6924.42 |
| (4,1,1) | 165874.5 | -82930.3 | 214478.6 | -107232 | 13750.18 | -6868.09 |
| (4,1,2) | 165876 | -82930 | 214458.2 | -107221 | 13751.46 | -6867.73 |
| (4,1,3) | 160017.5 | -79999.8 | 206902.8 | -103442 | 12481.58 | -6231.79 |
| (4,1,4) | 160585.2 | -80282.6 | 206903.9 | -103442 | 12944.25 | -6462.13 |

1. Patient 28

| ARIMA Model | ICP ARIMA AIC | ICP ARIMA LL | MAP ARIMA AIC | MAP ARIMA LL | PbtO2 ARIMA AIC | PbtO2 ARIMA LL |
| --- | --- | --- | --- | --- | --- | --- |
| (0,1,0) | 195635 | -97815.5 | 201806 | -100901 | 24245.7 | -12120.8 |
| (0,1,1) | 191671.7 | -95832.8 | 195033.7 | -97513.8 | 11605.8 | -5799.9 |
| (0,1,2) | 187955.4 | -93973.7 | 194806.6 | -97399.3 | 7505.787 | -3748.89 |
| (0,1,3) | 187685.6 | -93837.8 | 194790.6 | -97390.3 | 6612.837 | -3301.42 |
| (0,1,4) | 186947.4 | -93467.7 | 194782.6 | -97385.3 | 6530.999 | -3259.5 |
| (1,1,0) | 194354.7 | -97174.4 | 196811.2 | -98402.6 | 7133.493 | -3563.75 |
| (1,1,1) | 189580.2 | -94786.1 | 194787.5 | -97389.7 | 7133.354 | -3562.68 |
| (1,1,2) | 187819 | -93904.5 | 194738 | -97364 | 6644.506 | -3317.25 |
| (1,1,3) | 186741 | -93364.5 | 194685.2 | -97336.6 | 6551.454 | -3269.73 |
| (1,1,4) | 186638.9 | -93312.5 | 194699.7 | -97342.9 | 6532.742 | -3259.37 |
| (2,1,0) | 188793.7 | -94392.9 | 195443.3 | -97717.6 | 7132.443 | -3562.22 |
| (2,1,1) | 187176 | -93583 | 194777.5 | -97383.8 | 7079.657 | -3534.83 |
| (2,1,2) | 187103.7 | -93545.8 | 194787.4 | -97387.7 | 6285.188 | -3136.59 |
| (2,1,3) | 186658.7 | -93322.3 | 194580.3 | -97283.1 | 6287.179 | -3136.59 |
| (2,1,4) | 186638.7 | -93311.4 | 194530.3 | -97257.2 | 6239.278 | -3111.64 |
| (3,1,0) | 187560.5 | -93775.2 | 195022.3 | -97506.1 | 6478.023 | -3234.01 |
| (3,1,1) | 187150.2 | -93569.1 | 194735.4 | -97361.7 | 6308.46 | -3148.23 |
| (3,1,2) | 186635.9 | -93310.9 | 194695.6 | -97340.8 | 6287.321 | -3136.66 |
| (3,1,3) | 186608 | -93296 | 194543.6 | -97263.8 | 6288.487 | -3136.24 |
| (3,1,4) | 186638.9 | -93310.4 | 194586.5 | -97284.2 | 6290.078 | -3136.04 |
| (4,1,0) | 187101.2 | -93544.6 | 194888.5 | -97438.3 | 6313.365 | -3150.68 |
| (4,1,1) | 187069.3 | -93527.7 | 194696 | -97341 | 6297.507 | -3141.75 |
| (4,1,2) | 186613.2 | -93298.6 | 194689.4 | -97336.7 | 6303.12 | -3143.56 |
| (4,1,3) | 186607 | -93294.5 | 194529 | -97255.5 | 6290.246 | -3136.12 |
| (4,1,4) | 186606.9 | -93293.4 | 194531.1 | -97255.6 | 6286.222 | -3133.11 |

1. Patient 29

| ARIMA Model | ICP ARIMA AIC | ICP ARIMA LL | MAP ARIMA AIC | MAP ARIMA LL | PbtO2 ARIMA AIC | PbtO2 ARIMA LL |
| --- | --- | --- | --- | --- | --- | --- |
| (0,1,0) | 165143.9 | -82569.9 | 220655.8 | -110326 | 6972.627 | -3484.31 |
| (0,1,1) | 157637.4 | -78815.7 | 208013.6 | -104004 | -8989.81 | 4497.905 |
| (0,1,2) | 153594.8 | -76793.4 | 207995.6 | -103994 | -12048.5 | 6028.231 |
| (0,1,3) | 153527.3 | -76758.6 | 207898.4 | -103944 | -12957.5 | 6483.742 |
| (0,1,4) | 153489.8 | -76738.9 | 207820.6 | -103904 | -13285.1 | 6648.544 |
| (1,1,0) | 162231.7 | -81112.9 | 211319.9 | -105657 | -13328.5 | 6667.245 |
| (1,1,1) | 153800.6 | -76896.3 | 207992.4 | -103992 | -13396.9 | 6702.439 |
| (1,1,2) | 153510 | -76750 | 207971.5 | -103981 | -13414.5 | 6712.266 |
| (1,1,3) | 153366.1 | -76677 | 207853.4 | -103921 | -13419.9 | 6715.961 |
| (1,1,4) | 153368.4 | -76677.2 | 207822.6 | -103904 | -13428.5 | 6721.236 |
| (2,1,0) | 158039.4 | -79015.7 | 209696.3 | -104844 | -13391.6 | 6699.811 |
| (2,1,1) | 153591.7 | -76790.8 | 207922.9 | -103956 | -13416.5 | 6713.269 |
| (2,1,2) | 153474 | -76731 | 207871 | -103929 | -13415.9 | 6713.941 |
| (2,1,3) | 153366.9 | -76676.5 | 207784.6 | -103885 | -13413.6 | 6713.781 |
| (2,1,4) | 153369.7 | -76676.8 | 207771.1 | -103878 | -13420.4 | 6718.183 |
| (3,1,0) | 156555.4 | -78272.7 | 208347.5 | -104169 | -13413.3 | 6711.662 |
| (3,1,1) | 153401.6 | -76694.8 | 207825.1 | -103907 | -13416.2 | 6714.095 |
| (3,1,2) | 153354.6 | -76670.3 | 207827 | -103906 | -13415 | 6714.479 |
| (3,1,3) | 153458.2 | -76721.1 | 207769.4 | -103877 | -13413.2 | 6714.619 |
| (3,1,4) | 153370.5 | -76676.3 | 207766.5 | -103874 | -13649.7 | 6833.868 |
| (4,1,0) | 155527.2 | -77757.6 | 207871.7 | -103930 | -13422.4 | 6717.19 |
| (4,1,1) | 153381 | -76683.5 | 207825.8 | -103906 | -13414.2 | 6714.107 |
| (4,1,2) | 153404.2 | -76694.1 | 207817.7 | -103901 | -13413.4 | 6714.704 |
| (4,1,3) | 153349 | -76665.5 | 207761.1 | -103872 | -13411 | 6714.478 |
| (4,1,4) | 153342.9 | -76661.5 | 207767.4 | -103874 | -13647.7 | 6833.838 |

1. Patient 30

| ARIMA Model | ICP ARIMA AIC | ICP ARIMA LL | MAP ARIMA AIC | MAP ARIMA LL | PbtO2 ARIMA AIC | PbtO2 ARIMA LL |
| --- | --- | --- | --- | --- | --- | --- |
| (0,1,0) | 410209.3 | -205103 | 526031.7 | -263014 | 32610.26 | -16303.1 |
| (0,1,1) | 401788.7 | -200891 | 495722.1 | -247858 | 23754.96 | -11874.5 |
| (0,1,2) | 389925.7 | -194959 | 494906.8 | -247449 | 17768.61 | -8880.3 |
| (0,1,3) | 389793.9 | -194892 | 491396.1 | -245693 | 14184.59 | -7087.3 |
| (0,1,4) | 389779 | -194883 | 491202.4 | -245595 | 12382.78 | -6185.39 |
| (1,1,0) | 407171.4 | -203583 | 512685.3 | -256340 | 19064.23 | -9529.12 |
| (1,1,1) | 392070.6 | -196031 | 495335.6 | -247664 | 12760.94 | -6376.47 |
| (1,1,2) | 389806.2 | -194898 | 493181.2 | -246586 | 11581.12 | -5785.56 |
| (1,1,3) | 389788.6 | -194888 | 491331.1 | -245660 | 11331.34 | -5659.67 |
| (1,1,4) | 389724.1 | -194855 | 490535.8 | -245261 | 11275.88 | -5630.94 |
| (2,1,0) | 397904.3 | -198948 | 492131 | -246061 | 12719.55 | -6355.77 |
| (2,1,1) | 389793.4 | -194892 | 489907.6 | -244949 | 11822.87 | -5906.44 |
| (2,1,2) | 389751 | -194870 | 489810.6 | -244899 | 11226.05 | -5607.03 |
| (2,1,3) | 389677.7 | -194832 | 489233.2 | -244610 | 11172.84 | -5579.42 |
| (2,1,4) | 389504.6 | -194744 | 488992 | -244488 | 11191.88 | -5587.94 |
| (3,1,0) | 394990.7 | -197490 | 489624.8 | -244807 | 11606.51 | -5798.25 |
| (3,1,1) | 389772.5 | -194880 | 489600.9 | -244794 | 11608.25 | -5798.13 |
| (3,1,2) | 389712.1 | -194849 | 489602.9 | -244794 | 11123.57 | -5554.79 |
| (3,1,3) | 389637.7 | -194811 | 488928.6 | -244456 | 11229.78 | -5606.89 |
| (3,1,4) | 389657.7 | -194820 | 488923.4 | -244453 | 10936.4 | -5459.2 |
| (4,1,0) | 392456.8 | -196222 | 489603 | -244795 | 11608.06 | -5798.03 |
| (4,1,1) | 389610.1 | -194798 | 489628.8 | -244807 | 11604.88 | -5795.44 |
| (4,1,2) | 389566.4 | -194775 | 488762.5 | -244373 | 10921.1 | -5452.55 |
| (4,1,3) | 389526.8 | -194754 | 488711.5 | -244347 | 11048.42 | -5515.21 |
| (4,1,4) | 389525.2 | -194753 | 488927.5 | -244454 | 11227.89 | -5603.94 |

1. Patient 31

| ARIMA Model | ICP ARIMA AIC | ICP ARIMA LL | MAP ARIMA AIC | MAP ARIMA LL | PbtO2 ARIMA AIC | PbtO2 ARIMA LL |
| --- | --- | --- | --- | --- | --- | --- |
| (0,1,0) | 199089.9 | -99542.9 | 248190.8 | -124093 | -53810.5 | 26907.25 |
| (0,1,1) | 198652.9 | -99323.4 | 243128 | -121561 | -61164.6 | 30585.32 |
| (0,1,2) | 196454.5 | -98223.2 | 242993.2 | -121493 | -63613.8 | 31810.88 |
| (0,1,3) | 196350.7 | -98170.4 | 242948.9 | -121469 | -64679.3 | 32344.67 |
| (0,1,4) | 196233.6 | -98110.8 | 242941.8 | -121465 | -65111 | 32561.52 |
| (1,1,0) | 198824.5 | -99409.2 | 243968.4 | -121981 | -64160.9 | 32083.46 |
| (1,1,1) | 197238.3 | -98615.2 | 242972.6 | -121482 | -65538 | 32772.98 |
| (1,1,2) | 196388 | -98189 | 242962.7 | -121476 | -65536.4 | 32773.2 |
| (1,1,3) | 196281 | -98134.5 | 242891.3 | -121440 | -65534.5 | 32773.24 |
| (1,1,4) | 196215.8 | -98100.9 | 242889.1 | -121438 | -65533.2 | 32773.59 |
| (2,1,0) | 196671.7 | -98331.9 | 243310.6 | -121651 | -65368.1 | 32688.03 |
| (2,1,1) | 196243.3 | -98116.7 | 242960.4 | -121475 | -65536.4 | 32773.2 |
| (2,1,2) | 196178.1 | -98083 | 242879.1 | -121434 | -65534 | 32773.01 |
| (2,1,3) | 196172.4 | -98079.2 | 242840.3 | -121413 | -65532.8 | 32773.38 |
| (2,1,4) | 196161.7 | -98072.9 | 242031.9 | -121008 | -65530.9 | 32773.47 |
| (3,1,0) | 196212.8 | -98101.4 | 242969.3 | -121480 | -65520.4 | 32765.2 |
| (3,1,1) | 196202.8 | -98095.4 | 242942.6 | -121465 | -65534.4 | 32773.19 |
| (3,1,2) | 196176.2 | -98081.1 | 242875.2 | -121431 | -65532.8 | 32773.38 |
| (3,1,3) | 196178.7 | -98081.3 | 242034.3 | -121009 | -65531.2 | 32773.62 |
| (3,1,4) | 196156.5 | -98069.3 | 242805.8 | -121394 | -65529.4 | 32773.71 |
| (4,1,0) | 196201.3 | -98094.6 | 242953.6 | -121471 | -65530.2 | 32771.11 |
| (4,1,1) | 196203.4 | -98094.7 | 242150.6 | -121068 | -65525.4 | 32769.72 |
| (4,1,2) | 196161.2 | -98072.6 | 242157.3 | -121071 | -65530.9 | 32773.44 |
| (4,1,3) | 196162.1 | -98072 | 242073 | -121027 | -65529.4 | 32773.71 |
| (4,1,4) | 196143.2 | -98061.6 | 242798.5 | -121389 | -65527.4 | 32773.71 |

1. Patient 32

| ARIMA Model | ICP ARIMA AIC | ICP ARIMA LL | MAP ARIMA AIC | MAP ARIMA LL | PbtO2 ARIMA AIC | PbtO2 ARIMA LL |
| --- | --- | --- | --- | --- | --- | --- |
| (0,1,0) | 27301.75 | -13648.9 | 38335.87 | -19165.9 | 6725.385 | -3360.69 |
| (0,1,1) | 24821.7 | -12407.8 | 35958.49 | -17976.2 | 6669.664 | -3331.83 |
| (0,1,2) | 24808.11 | -12400.1 | 35867.21 | -17929.6 | 6589.117 | -3290.56 |
| (0,1,3) | 24808.43 | -12399.2 | 35855.85 | -17922.9 | 6545.521 | -3267.76 |
| (0,1,4) | 24805.15 | -12396.6 | 35845.52 | -17916.8 | 6487.11 | -3237.56 |
| (1,1,0) | 25840.58 | -12917.3 | 36232.4 | -18113.2 | 6639.861 | -3316.93 |
| (1,1,1) | 24808.93 | -12400.5 | 35855.8 | -17923.9 | 6452.602 | -3222.3 |
| (1,1,2) | 24809.36 | -12399.7 | 35855.41 | -17922.7 | 6426.106 | -3208.05 |
| (1,1,3) | 24809.42 | -12398.7 | 35855.11 | -17921.6 | 6427.04 | -3207.52 |
| (1,1,4) | 24806.85 | -12396.4 | 35827.46 | -17906.7 | 6427.885 | -3206.94 |
| (2,1,0) | 25280.42 | -12636.2 | 35948.66 | -17970.3 | 6515.879 | -3253.94 |
| (2,1,1) | 24807.54 | -12398.8 | 35854.62 | -17922.3 | 6428.621 | -3209.31 |
| (2,1,2) | 24808.33 | -12398.2 | 35856.66 | -17922.3 | 6426.804 | -3207.4 |
| (2,1,3) | 24791.38 | -12388.7 | 35744.98 | -17865.5 | 6429.84 | -3207.92 |
| (2,1,4) | 24792.13 | -12388.1 | 35827.1 | -17905.6 | 6427.432 | -3205.72 |
| (3,1,0) | 24960 | -12475 | 35908.88 | -17949.4 | 6465.672 | -3227.84 |
| (3,1,1) | 24803.91 | -12396 | 35856.62 | -17922.3 | 6428.65 | -3208.32 |
| (3,1,2) | 24805.6 | -12395.8 | 35789.62 | -17887.8 | 6432.621 | -3209.31 |
| (3,1,3) | 24792.21 | -12388.1 | 35746.99 | -17865.5 | 6428.833 | -3206.42 |
| (3,1,4) | 24794.06 | -12388 | 35738.49 | -17860.2 | 6426.799 | -3204.4 |
| (4,1,0) | 24834.86 | -12411.4 | 35797.83 | -17892.9 | 6426.859 | -3207.43 |
| (4,1,1) | 24803.24 | -12394.6 | 35756.22 | -17871.1 | 6425.431 | -3205.72 |
| (4,1,2) | 24790.63 | -12387.3 | 35750.28 | -17867.1 | 6426.708 | -3205.35 |
| (4,1,3) | 24742.28 | -12362.1 | 35753.29 | -17867.6 | 6428.647 | -3205.32 |
| (4,1,4) | 24747.16 | -12363.6 | 35728.08 | -17854 | 6428.085 | -3204.04 |

1. Patient 33

| ARIMA Model | ICP ARIMA AIC | ICP ARIMA LL | MAP ARIMA AIC | MAP ARIMA LL | PbtO2 ARIMA AIC | PbtO2 ARIMA LL |
| --- | --- | --- | --- | --- | --- | --- |
| (0,1,0) | 94096.7 | -47046.3 | 160686.2 | -80341.1 | -5549.46 | 2776.732 |
| (0,1,1) | 90514.17 | -45254.1 | 160609.8 | -80301.9 | -21597.7 | 10801.87 |
| (0,1,2) | 88859.15 | -44425.6 | 158637.5 | -79314.8 | -26348.5 | 13178.23 |
| (0,1,3) | 88823 | -44406.5 | 157813.6 | -78901.8 | -29120.9 | 14565.45 |
| (0,1,4) | 88819.1 | -44403.5 | 157763.3 | -78875.7 | -29511.1 | 14761.54 |
| (1,1,0) | 92095.96 | -46045 | 160638.7 | -80316.3 | -30030 | 15017.98 |
| (1,1,1) | 88942.35 | -44467.2 | 158386.2 | -79189.1 | -30122.9 | 15065.45 |
| (1,1,2) | 88820.62 | -44405.3 | 157785.9 | -78888 | -30146.6 | 15078.3 |
| (1,1,3) | 88822.62 | -44405.3 | 157748.4 | -78868.2 | -30172.7 | 15092.37 |
| (1,1,4) | 88818.03 | -44402 | 157750.3 | -78868.1 | -30218.5 | 15116.23 |
| (2,1,0) | 90192.01 | -45092 | 159386.9 | -79689.4 | -30128.9 | 15068.45 |
| (2,1,1) | 88820.25 | -44405.1 | 157739.2 | -78864.6 | -30131.1 | 15070.54 |
| (2,1,2) | 88822.84 | -44405.4 | 157413.1 | -78700.5 | -30118.9 | 15065.46 |
| (2,1,3) | 88823.79 | -44404.9 | 157750.7 | -78868.3 | -30336.6 | 15175.3 |
| (2,1,4) | 88735.08 | -44359.5 | 157752.4 | -78868.2 | -30337.2 | 15176.6 |
| (3,1,0) | 89488.91 | -44739.5 | 158638.8 | -79314.4 | -30138.1 | 15074.03 |
| (3,1,1) | 88822.22 | -44405.1 | 157728.6 | -78858.3 | -30256.6 | 15134.32 |
| (3,1,2) | 88820.82 | -44403.4 | 157736.9 | -78861.4 | -30258.7 | 15136.34 |
| (3,1,3) | 88818.94 | -44401.5 | 157419.9 | -78702 | -30336.5 | 15176.23 |
| (3,1,4) | 88725.04 | -44353.5 | 157171 | -78576.5 | -30333.9 | 15175.97 |
| (4,1,0) | 88872.69 | -44430.3 | 158148 | -79068 | -30189.5 | 15100.74 |
| (4,1,1) | 88762.68 | -44374.3 | 157682.1 | -78834 | -30335.4 | 15174.69 |
| (4,1,2) | 88757.69 | -44370.8 | 157724.4 | -78854.2 | -30334.3 | 15175.14 |
| (4,1,3) | 88734.34 | -44358.2 | 157078.8 | -78530.4 | -30337 | 15177.5 |
| (4,1,4) | 88726.52 | -44353.3 | 156469.7 | -78224.9 | -30400.3 | 15210.15 |

1. Patient 34

| ARIMA Model | ICP ARIMA AIC | ICP ARIMA LL | MAP ARIMA AIC | MAP ARIMA LL | PbtO2 ARIMA AIC | PbtO2 ARIMA LL |
| --- | --- | --- | --- | --- | --- | --- |
| (0,1,0) | 96908.95 | -48452.5 | 88284.44 | -44140.2 | -11137.2 | 5570.612 |
| (0,1,1) | 92842.16 | -46418.1 | 85822.64 | -42908.3 | -12514.1 | 6260.059 |
| (0,1,2) | 90597.24 | -45294.6 | 84810.95 | -42401.5 | -13418.8 | 6713.391 |
| (0,1,3) | 90552.53 | -45271.3 | 84670.32 | -42330.2 | -13576.6 | 6793.299 |
| (0,1,4) | 90526.54 | -45257.3 | 84664.82 | -42326.4 | -13590.3 | 6801.139 |
| (1,1,0) | 95804.01 | -47899 | 86893.72 | -43443.9 | -11856.5 | 5931.255 |
| (1,1,1) | 91222.05 | -45607 | 84732.12 | -42362.1 | -13494.9 | 6751.457 |
| (1,1,2) | 90560.7 | -45275.4 | 84702.9 | -42346.5 | -13536.2 | 6773.106 |
| (1,1,3) | 90516.98 | -45252.5 | 84666.68 | -42327.3 | -13597.9 | 6804.933 |
| (1,1,4) | 90514.23 | -45250.1 | 84663.41 | -42324.7 | -13598.5 | 6806.237 |
| (2,1,0) | 92914.28 | -46453.1 | 86000.69 | -42996.3 | -12484.9 | 6246.442 |
| (2,1,1) | 90565.39 | -45277.7 | 84695.3 | -42342.6 | -13546.4 | 6778.203 |
| (2,1,2) | 90535.75 | -45261.9 | 84678.81 | -42333.4 | -13553.4 | 6782.679 |
| (2,1,3) | 90507.43 | -45246.7 | 84664.39 | -42325.2 | -13598.6 | 6806.303 |
| (2,1,4) | 90517.34 | -45250.7 | 84662.24 | -42323.1 | -13596.1 | 6806.032 |
| (3,1,0) | 92147.02 | -46068.5 | 85251.04 | -42620.5 | -13116.7 | 6563.361 |
| (3,1,1) | 90553.91 | -45271 | 84667.33 | -42327.7 | -13562.6 | 6787.323 |
| (3,1,2) | 90520.51 | -45253.3 | 84666.24 | -42326.1 | -13592 | 6803.001 |
| (3,1,3) | 90496.2 | -45240.1 | 84662.11 | -42323.1 | -13596.5 | 6806.239 |
| (3,1,4) | 90463.31 | -45222.7 | 84661.13 | -42321.6 | -13594.7 | 6806.334 |
| (4,1,0) | 91301.89 | -45644.9 | 85012.02 | -42500 | -13233.2 | 6622.579 |
| (4,1,1) | 90484.32 | -45235.2 | 84667.41 | -42326.7 | -13578.9 | 6796.458 |
| (4,1,2) | 90482.06 | -45233 | 84668.55 | -42326.3 | -13594.5 | 6805.233 |
| (4,1,3) | 90483.9 | -45232.9 | 84659.04 | -42320.5 | -13593.8 | 6805.889 |
| (4,1,4) | 90489.83 | -45234.9 | 84634.18 | -42307.1 | -13594.1 | 6807.071 |

1. Patient 35

| ARIMA Model | ICP ARIMA AIC | ICP ARIMA LL | MAP ARIMA AIC | MAP ARIMA LL | PbtO2 ARIMA AIC | PbtO2 ARIMA LL |
| --- | --- | --- | --- | --- | --- | --- |
| (0,1,0) | 105061.2 | -52528.6 | 149967.9 | -74981.9 | -229.062 | 116.531 |
| (0,1,1) | 101789.3 | -50891.7 | 149572 | -74783 | -2059.39 | 1032.693 |
| (0,1,2) | 100553.7 | -50272.8 | 148191.5 | -74091.8 | -3443.54 | 1725.771 |
| (0,1,3) | 100489.1 | -50239.6 | 147655.8 | -73822.9 | -3998.9 | 2004.451 |
| (0,1,4) | 100363.9 | -50175.9 | 147259.2 | -73623.6 | -4266.66 | 2139.328 |
| (1,1,0) | 103277.7 | -51635.8 | 149714.5 | -74854.3 | -2840.86 | 1423.429 |
| (1,1,1) | 100830.8 | -50411.4 | 147052.9 | -73522.4 | -4258.73 | 2133.364 |
| (1,1,2) | 100447.2 | -50218.6 | 146894.8 | -73442.4 | -4433.71 | 2221.854 |
| (1,1,3) | 100439.2 | -50213.6 | 146848.9 | -73418.5 | -4438.38 | 2225.189 |
| (1,1,4) | 99798.85 | -49892.4 | 146819.7 | -73402.8 | -4445.26 | 2229.631 |
| (2,1,0) | 100836.6 | -50414.3 | 148676.5 | -74334.3 | -4227.75 | 2117.877 |
| (2,1,1) | 100666 | -50328 | 146916.8 | -73453.4 | -4420.71 | 2215.356 |
| (2,1,2) | 100429 | -50208.5 | 146845.5 | -73416.8 | -4444.34 | 2228.171 |
| (2,1,3) | 99810.07 | -49898 | 146840.1 | -73413 | -4432.48 | 2223.24 |
| (2,1,4) | 99792.49 | -49888.2 | 146396.1 | -73190.1 | -4436.48 | 2226.239 |
| (3,1,0) | 100763.8 | -50376.9 | 148517.5 | -74253.8 | -4424.02 | 2217.01 |
| (3,1,1) | 99823.63 | -49905.8 | 146856.3 | -73422.2 | -4429.31 | 2220.653 |
| (3,1,2) | 99825.5 | -49905.8 | 146837 | -73411.5 | -4424.68 | 2219.338 |
| (3,1,3) | 99792.82 | -49888.4 | 146815.9 | -73400 | -4440.95 | 2228.475 |
| (3,1,4) | 99794.62 | -49888.3 | 146722 | -73352 | -4440.87 | 2229.433 |
| (4,1,0) | 100596.8 | -50292.4 | 148381.5 | -74184.8 | -4430.82 | 2221.41 |
| (4,1,1) | 99825.53 | -49905.8 | 146792.6 | -73389.3 | -4428.7 | 2221.348 |
| (4,1,2) | 99804.87 | -49894.4 | 146566.2 | -73275.1 | -4427.11 | 2221.555 |
| (4,1,3) | 99790.63 | -49886.3 | 146708.5 | -73345.3 | -4440.11 | 2229.053 |
| (4,1,4) | 99792.09 | -49886 | 146676.4 | -73328.2 | -4442.71 | 2231.355 |

1. Patient 36

| ARIMA Model | ICP ARIMA AIC | ICP ARIMA LL | MAP ARIMA AIC | MAP ARIMA LL | PbtO2 ARIMA AIC | PbtO2 ARIMA LL |
| --- | --- | --- | --- | --- | --- | --- |
| (0,1,0) | 34326.22 | -17161.1 | 68927.27 | -34461.6 | -3759.36 | 1881.682 |
| (0,1,1) | 32445.72 | -16219.9 | 66051.24 | -33022.6 | -3989.52 | 1997.762 |
| (0,1,2) | 30407.24 | -15199.6 | 65856.16 | -32924.1 | -4174.73 | 2091.367 |
| (0,1,3) | 30406.29 | -15198.1 | 65850.3 | -32920.2 | -4270.99 | 2140.493 |
| (0,1,4) | 30332.79 | -15160.4 | 65831.36 | -32909.7 | -4328.13 | 2170.064 |
| (1,1,0) | 33882.9 | -16938.5 | 67287.44 | -33640.7 | -4031.32 | 2018.658 |
| (1,1,1) | 30909.65 | -15450.8 | 65881.89 | -32936.9 | -4467.65 | 2237.824 |
| (1,1,2) | 30407.21 | -15198.6 | 65853.42 | -32921.7 | -4473.39 | 2241.696 |
| (1,1,3) | 30394.12 | -15191.1 | 65834.01 | -32911 | -4479.88 | 2245.942 |
| (1,1,4) | 30328.49 | -15157.2 | 65829.17 | -32907.6 | -4484.45 | 2249.226 |
| (2,1,0) | 32084.15 | -16038.1 | 66393.29 | -33192.6 | -4281.86 | 2144.929 |
| (2,1,1) | 30320.37 | -15155.2 | 65839.88 | -32914.9 | -4474.02 | 2242.011 |
| (2,1,2) | 30320.31 | -15154.2 | 65825.88 | -32906.9 | -4473.99 | 2242.996 |
| (2,1,3) | 30319.64 | -15152.8 | 65829.69 | -32907.8 | -4470.07 | 2242.037 |
| (2,1,4) | 30320.38 | -15152.2 | 65829.52 | -32906.8 | -4478.25 | 2247.125 |
| (3,1,0) | 31281.03 | -15635.5 | 66005.81 | -32997.9 | -4380.24 | 2195.122 |
| (3,1,1) | 30319.99 | -15154 | 65832.38 | -32910.2 | -4479.69 | 2245.843 |
| (3,1,2) | 30323.37 | -15154.7 | 65836.21 | -32911.1 | -4470.46 | 2242.23 |
| (3,1,3) | 30321.4 | -15152.7 | 65829.52 | -32906.8 | -4472.16 | 2244.081 |
| (3,1,4) | 30300.99 | -15141.5 | 65829.58 | -32905.8 | -4471.99 | 2244.995 |
| (4,1,0) | 30908.78 | -15448.4 | 65884.29 | -32936.1 | -4413.77 | 2212.883 |
| (4,1,1) | 30319.42 | -15152.7 | 65832.58 | -32909.3 | -4487.01 | 2250.504 |
| (4,1,2) | 30321.46 | -15152.7 | 65830.04 | -32907 | -4478.6 | 2247.298 |
| (4,1,3) | 30322.93 | -15152.5 | 65829.77 | -32905.9 | -4471.96 | 2244.981 |
| (4,1,4) | 30305.03 | -15142.5 | 65830.2 | -32905.1 | -4470.11 | 2245.053 |

1. Patient 37

| ARIMA Model | ICP ARIMA AIC | ICP ARIMA LL | MAP ARIMA AIC | MAP ARIMA LL | PbtO2 ARIMA AIC | PbtO2 ARIMA LL |
| --- | --- | --- | --- | --- | --- | --- |
| (0,1,0) | 9223.29 | -4609.65 | 28897.68 | -14446.8 | 6995.44 | -3495.72 |
| (0,1,1) | 8223.142 | -4108.57 | 28240.73 | -14117.4 | 6964.226 | -3479.11 |
| (0,1,2) | 8083.353 | -4037.68 | 27771.62 | -13881.8 | 6943.184 | -3467.59 |
| (0,1,3) | 8083.208 | -4036.6 | 27756.86 | -13873.4 | 6922.333 | -3456.17 |
| (0,1,4) | 8045.861 | -4016.93 | 27572.6 | -13780.3 | 6913.808 | -3450.9 |
| (1,1,0) | 8760.805 | -4377.4 | 28747.79 | -14370.9 | 6955.55 | -3474.77 |
| (1,1,1) | 8109.077 | -4050.54 | 27984.26 | -13988.1 | 6872.949 | -3432.47 |
| (1,1,2) | 8084.448 | -4037.22 | 27768.3 | -13879.2 | 6871.315 | -3430.66 |
| (1,1,3) | 8075.569 | -4031.78 | 27719.78 | -13853.9 | 6870.485 | -3429.24 |
| (1,1,4) | 8032.407 | -4009.2 | 27564.98 | -13775.5 | 6872.035 | -3429.02 |
| (2,1,0) | 8385.448 | -4188.72 | 27820.96 | -13906.5 | 6925.714 | -3458.86 |
| (2,1,1) | 8061.272 | -4025.64 | 27564.12 | -13777.1 | 6871.682 | -3430.84 |
| (2,1,2) | 8045.328 | -4016.66 | 27540.09 | -13764 | 6876.675 | -3432.34 |
| (2,1,3) | 8045.39 | -4015.7 | 27405.29 | -13695.6 | 6873.468 | -3429.73 |
| (2,1,4) | 8034.026 | -4009.01 | 27406.16 | -13695.1 | 6874.436 | -3429.22 |
| (3,1,0) | 8101.915 | -4045.96 | 27473.25 | -13731.6 | 6898.77 | -3444.38 |
| (3,1,1) | 8039.133 | -4013.57 | 27460.91 | -13724.5 | 6871.134 | -3429.57 |
| (3,1,2) | 8035.204 | -4010.6 | 27462.23 | -13724.1 | 6873.729 | -3429.86 |
| (3,1,3) | 8036.623 | -4010.31 | 27406.36 | -13695.2 | 6875.917 | -3429.96 |
| (3,1,4) | 8035.727 | -4008.86 | 27407.34 | -13694.7 | 6876.818 | -3429.41 |
| (4,1,0) | 8069.946 | -4028.97 | 27461.82 | -13724.9 | 6890.027 | -3439.01 |
| (4,1,1) | 8038.763 | -4012.38 | 27462.74 | -13724.4 | 6872.888 | -3429.44 |
| (4,1,2) | 8037.024 | -4010.51 | 27418.91 | -13701.5 | 6875.073 | -3429.54 |
| (4,1,3) | 8036.97 | -4009.48 | 27402.56 | -13692.3 | 6877.464 | -3429.73 |
| (4,1,4) | 8037.678 | -4008.84 | 27403.81 | -13691.9 | 6866.59 | -3423.3 |

1. Patient 38

| ARIMA Model | ICP ARIMA AIC | ICP ARIMA LL | MAP ARIMA AIC | MAP ARIMA LL | PbtO2 ARIMA AIC | PbtO2 ARIMA LL |
| --- | --- | --- | --- | --- | --- | --- |
| (0,1,0) | 180161.7 | -90078.9 | 264434.1 | -132215 | -32085.5 | 16044.75 |
| (0,1,1) | 171253.3 | -85623.7 | 247657.7 | -123826 | -34069.2 | 17037.59 |
| (0,1,2) | 170269.6 | -85130.8 | 247414.1 | -123703 | -34167.2 | 17087.6 |
| (0,1,3) | 168862.7 | -84426.4 | 242701.7 | -121346 | -34416.9 | 17213.46 |
| (0,1,4) | 168823.1 | -84405.5 | 242066.7 | -121027 | -34620.1 | 17316.05 |
| (1,1,0) | 175998.8 | -87996.4 | 258751.1 | -129373 | -34079 | 17042.49 |
| (1,1,1) | 170756.8 | -85374.4 | 247594.2 | -123793 | -34128.3 | 17068.17 |
| (1,1,2) | 169198.7 | -84594.4 | 244549.7 | -122270 | -35114.6 | 17562.32 |
| (1,1,3) | 168829.2 | -84408.6 | 242371.1 | -121180 | -35197.7 | 17604.85 |
| (1,1,4) | 168815.2 | -84400.6 | 241754.3 | -120870 | -35200.1 | 17607.03 |
| (2,1,0) | 169316.4 | -84654.2 | 242845.7 | -121419 | -34155 | 17081.49 |
| (2,1,1) | 169071.5 | -84530.7 | 242074.8 | -121032 | -34172.8 | 17091.39 |
| (2,1,2) | 168816.4 | -84402.2 | 241724.2 | -120856 | -35202.7 | 17607.33 |
| (2,1,3) | 168814 | -84400 | 241673.4 | -120830 | -35183.4 | 17598.68 |
| (2,1,4) | 168795.2 | -84389.6 | 241553.2 | -120769 | -35195.2 | 17605.61 |
| (3,1,0) | 169176.6 | -84583.3 | 242340.9 | -121165 | -34228.3 | 17119.14 |
| (3,1,1) | 168949.4 | -84468.7 | 241870.7 | -120929 | -35032.3 | 17522.13 |
| (3,1,2) | 168815.9 | -84400.9 | 241660.9 | -120823 | -35201.5 | 17607.74 |
| (3,1,3) | 168814.9 | -84399.4 | 241659.7 | -120822 | -35198.8 | 17607.38 |
| (3,1,4) | 168805.5 | -84393.7 | 241391.1 | -120687 | -35197.7 | 17607.86 |
| (4,1,0) | 168812.2 | -84400.1 | 241703.5 | -120846 | -34469.5 | 17240.74 |
| (4,1,1) | 168814.2 | -84400.1 | 241660.4 | -120823 | -35143.2 | 17578.62 |
| (4,1,2) | 168808.6 | -84396.3 | 241647.7 | -120816 | -35200.6 | 17608.28 |
| (4,1,3) | 168811.1 | -84396.6 | 241404.6 | -120693 | -35197.6 | 17607.79 |
| (4,1,4) | 168812.8 | -84396.4 | 241382.6 | -120681 | -35195.2 | 17607.61 |

1. Patient 39

| ARIMA Model | ICP ARIMA AIC | ICP ARIMA LL | MAP ARIMA AIC | MAP ARIMA LL | PbtO2 ARIMA AIC | PbtO2 ARIMA LL |
| --- | --- | --- | --- | --- | --- | --- |
| (0,1,0) | 191266.1 | -95631.1 | 468423.9 | -234210 | 96476.48 | -48236.2 |
| (0,1,1) | 189402.8 | -94698.4 | 457912.7 | -228953 | 86251.16 | -43122.6 |
| (0,1,2) | 185947.3 | -92969.7 | 450261 | -225127 | 83134.07 | -41563 |
| (0,1,3) | 185932.3 | -92961.1 | 450226.9 | -225108 | 81896.22 | -40943.1 |
| (0,1,4) | 185548.7 | -92768.4 | 449127.2 | -224558 | 81312.29 | -40650.1 |
| (1,1,0) | 190149.2 | -95071.6 | 464686.5 | -232340 | 82779.69 | -41386.8 |
| (1,1,1) | 187450.2 | -93721.1 | 452764.8 | -226378 | 81235.17 | -40613.6 |
| (1,1,2) | 185939.6 | -92964.8 | 450243.3 | -225117 | 81218.32 | -40604.2 |
| (1,1,3) | 185864.3 | -92926.1 | 450058.3 | -225023 | 81191.95 | -40590 |
| (1,1,4) | 185481.7 | -92733.9 | 448859.9 | -224423 | 81176.32 | -40581.2 |
| (2,1,0) | 185744.9 | -92868.4 | 452940.8 | -226466 | 81338.86 | -40665.4 |
| (2,1,1) | 185610.2 | -92800.1 | 449429.2 | -224710 | 81220.93 | -40605.5 |
| (2,1,2) | 185564.5 | -92776.3 | 449426.1 | -224707 | 81233.01 | -40610.5 |
| (2,1,3) | 185455.1 | -92720.5 | 448786.2 | -224386 | 81211.77 | -40598.9 |
| (2,1,4) | 185450.2 | -92717.1 | 448751.7 | -224368 | 81179.76 | -40581.9 |
| (3,1,0) | 185576 | -92783 | 449896 | -224943 | 81205.88 | -40597.9 |
| (3,1,1) | 185459.4 | -92723.7 | 449418.8 | -224703 | 81207.66 | -40597.8 |
| (3,1,2) | 185454.8 | -92720.4 | 449198.3 | -224592 | 81215.29 | -40600.6 |
| (3,1,3) | 185459.6 | -92721.8 | 448739.3 | -224362 | 81050.28 | -40517.1 |
| (3,1,4) | 185450 | -92716 | 448736.2 | -224359 | 81173.42 | -40577.7 |
| (4,1,0) | 185502.7 | -92745.4 | 449859.3 | -224924 | 81207.46 | -40597.7 |
| (4,1,1) | 185455.9 | -92721 | 449064.8 | -224525 | 81207.5 | -40596.8 |
| (4,1,2) | 185463.4 | -92723.7 | 448775.8 | -224380 | 81165.29 | -40574.6 |
| (4,1,3) | 185105 | -92543.5 | 448699.6 | -224341 | 81171.83 | -40576.9 |
| (4,1,4) | 185124.5 | -92552.2 | 448638 | -224309 | 81172.67 | -40576.3 |

1. Patient 40

| ARIMA Model | ICP ARIMA AIC | ICP ARIMA LL | MAP ARIMA AIC | MAP ARIMA LL | PbtO2 ARIMA AIC | PbtO2 ARIMA LL |
| --- | --- | --- | --- | --- | --- | --- |
| (0,1,0) | 93423.73 | -46709.9 | 109846.4 | -54921.2 | -4622.32 | 2313.158 |
| (0,1,1) | 87715.96 | -43855 | 105002.8 | -52498.4 | -18991.8 | 9498.924 |
| (0,1,2) | 85855.2 | -42923.6 | 104358 | -52175 | -25465.6 | 12736.8 |
| (0,1,3) | 85837.52 | -42913.8 | 104319.3 | -52154.6 | -28434.3 | 14222.17 |
| (0,1,4) | 85671.4 | -42829.7 | 104318.2 | -52153.1 | -29537.7 | 14774.84 |
| (1,1,0) | 92026.43 | -46010.2 | 107676.4 | -53835.2 | -30199.7 | 15102.86 |
| (1,1,1) | 86242.92 | -43117.5 | 104478.5 | -52235.2 | -30319.6 | 15163.78 |
| (1,1,2) | 85844.7 | -42917.3 | 104319.5 | -52154.8 | -30568.1 | 15289.05 |
| (1,1,3) | 85495.04 | -42741.5 | 104320.1 | -52154 | -30566.7 | 15289.37 |
| (1,1,4) | 85398.95 | -42692.5 | 104322.9 | -52154.4 | -30598.5 | 15306.23 |
| (2,1,0) | 90018.35 | -45005.2 | 105498.9 | -52745.4 | -30348 | 15178.01 |
| (2,1,1) | 85676.92 | -42833.5 | 104350.4 | -52170.2 | -30426.4 | 15218.18 |
| (2,1,2) | 85351.11 | -42669.6 | 104319 | -52153.5 | -30446.2 | 15229.09 |
| (2,1,3) | 85388.66 | -42687.3 | 104308.3 | -52147.1 | -30571.9 | 15292.94 |
| (2,1,4) | 85324.61 | -42654.3 | 104304 | -52144 | -30597.1 | 15306.54 |
| (3,1,0) | 88998.12 | -44494.1 | 104946.2 | -52468.1 | -30525.4 | 15267.69 |
| (3,1,1) | 85595.71 | -42791.9 | 104295.4 | -52141.7 | -30535.5 | 15273.73 |
| (3,1,2) | 85346.26 | -42666.1 | 104247.7 | -52116.9 | -30613.9 | 15313.97 |
| (3,1,3) | 85377.01 | -42680.5 | 104304.6 | -52144.3 | -30619.7 | 15317.86 |
| (3,1,4) | 85314.01 | -42648 | 104233.6 | -52107.8 | -30631 | 15324.52 |
| (4,1,0) | 87866.18 | -43927.1 | 104662.7 | -52325.3 | -30548.4 | 15280.2 |
| (4,1,1) | 85389.6 | -42687.8 | 104288.7 | -52137.4 | -30560 | 15287 |
| (4,1,2) | 85301 | -42642.5 | 104293.8 | -52138.9 | -30620.8 | 15318.42 |
| (4,1,3) | 85306.61 | -42644.3 | 104242.7 | -52112.4 | -30611.6 | 15314.82 |
| (4,1,4) | 85310.61 | -42645.3 | 104225.8 | -52102.9 | -30615.8 | 15317.88 |

1. Patient 41

| ARIMA Model | ICP ARIMA AIC | ICP ARIMA LL | MAP ARIMA AIC | MAP ARIMA LL | PbtO2 ARIMA AIC | PbtO2 ARIMA LL |
| --- | --- | --- | --- | --- | --- | --- |
| (0,1,0) | 145077 | -72536.5 | 365129.5 | -182563 | 23161.26 | -11578.6 |
| (0,1,1) | 137928.8 | -68961.4 | 361771.8 | -180883 | 22616.55 | -11305.3 |
| (0,1,2) | 137218.6 | -68605.3 | 354207.6 | -177100 | 21495.18 | -10743.6 |
| (0,1,3) | 137147.3 | -68568.7 | 354071.5 | -177031 | 21052.8 | -10521.4 |
| (0,1,4) | 137050.8 | -68519.4 | 353982 | -176985 | 21028.44 | -10508.2 |
| (1,1,0) | 140063.7 | -70028.9 | 363873.3 | -181934 | 22485.36 | -11239.7 |
| (1,1,1) | 137343 | -68667.5 | 356066.9 | -178029 | 22206.51 | -11099.3 |
| (1,1,2) | 137135.7 | -68562.9 | 354091.6 | -177041 | 21125.21 | -10557.6 |
| (1,1,3) | 137132.7 | -68560.4 | 353768.8 | -176878 | 21038.81 | -10513.4 |
| (1,1,4) | 136292.1 | -68139 | 353738.3 | -176862 | 21019.93 | -10503 |
| (2,1,0) | 137248.9 | -68620.5 | 356953.3 | -178473 | 21736.58 | -10864.3 |
| (2,1,1) | 137163.6 | -68576.8 | 354319.5 | -177155 | 21360.77 | -10675.4 |
| (2,1,2) | 137122.2 | -68555.1 | 354063.7 | -177026 | 20986.01 | -10487 |
| (2,1,3) | 136664.6 | -68325.3 | 353719 | -176853 | 20969.24 | -10477.6 |
| (2,1,4) | 136293 | -68138.5 | 353369.9 | -176677 | 20986.87 | -10485.4 |
| (3,1,0) | 137183.5 | -68586.8 | 355853.2 | -177922 | 21185.81 | -10587.9 |
| (3,1,1) | 136213.6 | -68100.8 | 353979.6 | -176984 | 21174.72 | -10581.4 |
| (3,1,2) | 136215.4 | -68100.7 | 353980.8 | -176983 | 20974.29 | -10480.1 |
| (3,1,3) | 136203.8 | -68093.9 | 353215.7 | -176600 | 20979.76 | -10481.9 |
| (3,1,4) | 135515.5 | -67748.8 | 353102.1 | -176542 | 20970.39 | -10476.2 |
| (4,1,0) | 137177.7 | -68582.9 | 355166.8 | -177577 | 21162.33 | -10575.2 |
| (4,1,1) | 136215.3 | -68100.7 | 353980.6 | -176983 | 21143.25 | -10564.6 |
| (4,1,2) | 136139.7 | -68061.9 | 353859.2 | -176922 | 20968.36 | -10476.2 |
| (4,1,3) | 135993.5 | -67987.7 | 351993.7 | -175988 | 20977.73 | -10479.9 |
| (4,1,4) | 135763.4 | -67871.7 | 352024.8 | -176002 | 20982.24 | -10481.1 |

1. Patient 42

| ARIMA Model | ICP ARIMA AIC | ICP ARIMA LL | MAP ARIMA AIC | MAP ARIMA LL | PbtO2 ARIMA AIC | PbtO2 ARIMA LL |
| --- | --- | --- | --- | --- | --- | --- |
| (0,1,0) | 155389.8 | -77692.9 | 140609 | -70302.5 | 28029.74 | -14012.9 |
| (0,1,1) | 153959.3 | -76976.7 | 136118.7 | -68056.3 | 23559.55 | -11776.8 |
| (0,1,2) | 152326.7 | -76159.4 | 136089.7 | -68040.9 | 20597.68 | -10294.8 |
| (0,1,3) | 152328.7 | -76159.3 | 136089.4 | -68039.7 | 19064.4 | -9527.2 |
| (0,1,4) | 152329.6 | -76158.8 | 136021.5 | -68004.8 | 18888.45 | -9438.23 |
| (1,1,0) | 154670.7 | -77332.3 | 137067.8 | -68530.9 | 20624.27 | -10309.1 |
| (1,1,1) | 152722.5 | -76357.3 | 136087.7 | -68039.9 | 18314.16 | -9153.08 |
| (1,1,2) | 152328.7 | -76159.3 | 136093.3 | -68041.7 | 18058.66 | -9024.33 |
| (1,1,3) | 152330.6 | -76159.3 | 136093.7 | -68040.9 | 18046.18 | -9017.09 |
| (1,1,4) | 152317.8 | -76151.9 | 136012.6 | -67999.3 | 18017.25 | -9001.62 |
| (2,1,0) | 152774.8 | -76383.4 | 136263.7 | -68127.8 | 18218.02 | -9105.01 |
| (2,1,1) | 152379.7 | -76184.8 | 136079.5 | -68034.7 | 18062.85 | -9026.43 |
| (2,1,2) | 152330 | -76159 | 136091.5 | -68039.8 | 18053.65 | -9020.82 |
| (2,1,3) | 152315.2 | -76150.6 | 135883.4 | -67934.7 | 18032.4 | -9009.2 |
| (2,1,4) | 152298.7 | -76141.3 | 136012.2 | -67998.1 | 17958.97 | -8971.49 |
| (3,1,0) | 152565.1 | -76277.6 | 136193.9 | -68091.9 | 18027.2 | -9008.6 |
| (3,1,1) | 151983.9 | -75985.9 | 135930.8 | -67959.4 | 17970.42 | -8979.21 |
| (3,1,2) | 151900.8 | -75943.4 | 135886 | -67936 | 17969.99 | -8978 |
| (3,1,3) | 152290.9 | -76137.4 | 135782.9 | -67883.4 | 17886.73 | -8935.37 |
| (3,1,4) | 151879.2 | -75930.6 | 135510.7 | -67746.3 | 17887.8 | -8934.9 |
| (4,1,0) | 152412 | -76200 | 136149.5 | -68068.8 | 17992.82 | -8990.41 |
| (4,1,1) | 151929.8 | -75957.9 | 135908.9 | -67947.4 | 17971.28 | -8978.64 |
| (4,1,2) | 151941.9 | -75963 | 135913 | -67948.5 | 17951.85 | -8967.93 |
| (4,1,3) | 151903.9 | -75943 | 135660.3 | -67821.1 | 17888.04 | -8935.02 |
| (4,1,4) | 151880.8 | -75930.4 | 135483 | -67731.5 | 17884.52 | -8932.26 |

1. Patient 43

| ARIMA Model | ICP ARIMA AIC | ICP ARIMA LL | MAP ARIMA AIC | MAP ARIMA LL | PbtO2 ARIMA AIC | PbtO2 ARIMA LL |
| --- | --- | --- | --- | --- | --- | --- |
| (0,1,0) | 49296.62 | -24646.3 | 63704.01 | -31850 | -6030.46 | 3017.232 |
| (0,1,1) | 48966.51 | -24480.3 | 61388.4 | -30691.2 | -6327.86 | 3166.928 |
| (0,1,2) | 48966.48 | -24479.2 | 61350.26 | -30671.1 | -6416.46 | 3212.23 |
| (0,1,3) | 48959.86 | -24474.9 | 61341.36 | -30665.7 | -6486.23 | 3248.116 |
| (0,1,4) | 48956.43 | -24472.2 | 61343.23 | -30665.6 | -6544.73 | 3278.365 |
| (1,1,0) | 48978.31 | -24486.2 | 61932.76 | -30963.4 | -6369.39 | 3187.695 |
| (1,1,1) | 48965.44 | -24478.7 | 61345.6 | -30668.8 | -6374.3 | 3191.148 |
| (1,1,2) | 48862.31 | -24426.2 | 61342.95 | -30666.5 | -6692.77 | 3351.385 |
| (1,1,3) | 48863.14 | -24425.6 | 61342.89 | -30665.4 | -6696.38 | 3354.189 |
| (1,1,4) | 48871.21 | -24428.6 | 61344.89 | -30665.4 | -6696.97 | 3355.484 |
| (2,1,0) | 48970.47 | -24481.2 | 61565.29 | -30778.6 | -6380.53 | 3194.266 |
| (2,1,1) | 48861.23 | -24425.6 | 61342.12 | -30666.1 | -6620.32 | 3315.162 |
| (2,1,2) | 48862.93 | -24425.5 | 61341.18 | -30664.6 | -6697.24 | 3354.622 |
| (2,1,3) | 48865.56 | -24425.8 | 61342.72 | -30664.4 | -6689.47 | 3351.735 |
| (2,1,4) | 48866.87 | -24425.4 | 61336.59 | -30660.3 | -6693.47 | 3354.735 |
| (3,1,0) | 48964.45 | -24477.2 | 61404.39 | -30697.2 | -6461.37 | 3235.683 |
| (3,1,1) | 48862.9 | -24425.5 | 61343.23 | -30665.6 | -6677.53 | 3344.767 |
| (3,1,2) | 48864.9 | -24425.4 | 61342.65 | -30664.3 | -6697.54 | 3355.77 |
| (3,1,3) | 48866.87 | -24425.4 | 61344.54 | -30664.3 | -6694.3 | 3355.15 |
| (3,1,4) | 48869.27 | -24425.6 | 61325.81 | -30653.9 | -6688.11 | 3353.056 |
| (4,1,0) | 48959.31 | -24473.7 | 61372.71 | -30680.4 | -6550.82 | 3281.412 |
| (4,1,1) | 48864.75 | -24425.4 | 61343.05 | -30664.5 | -6688.68 | 3351.342 |
| (4,1,2) | 48866.82 | -24425.4 | 61343.39 | -30663.7 | -6687.42 | 3351.711 |
| (4,1,3) | 48868.85 | -24425.4 | 61344.69 | -30663.3 | -6693.6 | 3355.802 |
| (4,1,4) | 48870.83 | -24425.4 | 61318.18 | -30649.1 | -6689.96 | 3354.981 |

1. Patient 44

| ARIMA Model | ICP ARIMA AIC | ICP ARIMA LL | MAP ARIMA AIC | MAP ARIMA LL | PbtO2 ARIMA AIC | PbtO2 ARIMA LL |
| --- | --- | --- | --- | --- | --- | --- |
| (0,1,0) | 364828.1 | -182412 | 353802.6 | -176899 | 119705.4 | -59850.7 |
| (0,1,1) | 359821.3 | -179908 | 351375.4 | -175685 | 87606.56 | -43800.3 |
| (0,1,2) | 350038.5 | -175015 | 344162.5 | -172077 | 76257.16 | -38124.6 |
| (0,1,3) | 349781.6 | -174886 | 344164.5 | -172077 | 74198.79 | -37094.4 |
| (0,1,4) | 349601.3 | -174795 | 342436.5 | -171212 | 74193 | -37090.5 |
| (1,1,0) | 363442.9 | -181718 | 352564.4 | -176279 | 81021.67 | -40507.8 |
| (1,1,1) | 354558.5 | -177275 | 345835.8 | -172914 | 78075.79 | -39033.9 |
| (1,1,2) | 349840 | -174915 | 344164.4 | -172077 | 74876.84 | -37433.4 |
| (1,1,3) | 349678.5 | -174833 | 344122.9 | -172055 | 74197.74 | -37092.9 |
| (1,1,4) | 349546.2 | -174766 | 342349.9 | -171168 | 71831.12 | -35908.6 |
| (2,1,0) | 351788.6 | -175890 | 347111.3 | -173552 | 75757.24 | -37874.6 |
| (2,1,1) | 350390.7 | -175190 | 346280.1 | -173135 | 71599.06 | -35794.5 |
| (2,1,2) | 349609.4 | -174799 | 343384 | -171686 | 71194.5 | -35591.3 |
| (2,1,3) | 349552.6 | -174769 | 343009.2 | -171498 | 70811.88 | -35398.9 |
| (2,1,4) | 349548.1 | -174766 | 336392.2 | -168188 | 70805.57 | -35394.8 |
| (3,1,0) | 351277 | -175633 | 346981 | -173485 | 71262.04 | -35626 |
| (3,1,1) | 349872.2 | -174930 | 346255.7 | -173122 | 71010.09 | -35499 |
| (3,1,2) | 349606.2 | -174796 | 342569.6 | -171278 | 70950.98 | -35468.5 |
| (3,1,3) | 349553.6 | -174769 | 335805.3 | -167895 | 70807.7 | -35395.8 |
| (3,1,4) | 349540.6 | -174761 | 334396.2 | -167189 | 70811.44 | -35396.7 |
| (4,1,0) | 349840.1 | -174914 | 342343.8 | -171166 | 71072.79 | -35530.4 |
| (4,1,1) | 349512.7 | -174749 | 341295.3 | -170641 | 70658.86 | -35322.4 |
| (4,1,2) | 349514.6 | -174749 | 341179.8 | -170582 | 70693.83 | -35338.9 |
| (4,1,3) | 348996.2 | -174489 | 334449.5 | -167216 | 70798.43 | -35390.2 |
| (4,1,4) | 348666.2 | -174323 | 334391.4 | -167186 | 70810.26 | -35395.1 |

1. Patient 45

| ARIMA Model | ICP ARIMA AIC | ICP ARIMA LL | MAP ARIMA AIC | MAP ARIMA LL | PbtO2 ARIMA AIC | PbtO2 ARIMA LL |
| --- | --- | --- | --- | --- | --- | --- |
| (0,1,0) | 31682.39 | -15839.2 | 63007.16 | -31501.6 | -16955 | 8479.522 |
| (0,1,1) | 31381.59 | -15687.8 | 59892.6 | -29943.3 | -17545.2 | 8775.604 |
| (0,1,2) | 29798.41 | -14895.2 | 59537.07 | -29764.5 | -17746.2 | 8877.114 |
| (0,1,3) | 29194.07 | -14592 | 59469.11 | -29729.6 | -17755.5 | 8882.735 |
| (0,1,4) | 29186.02 | -14587 | 59456.97 | -29722.5 | -17755.6 | 8883.806 |
| (1,1,0) | 31515.27 | -15754.6 | 61239.19 | -30616.6 | -17676.6 | 8841.295 |
| (1,1,1) | 29499.62 | -14745.8 | 59464.8 | -29728.4 | -17806.6 | 8907.315 |
| (1,1,2) | 29327.57 | -14658.8 | 59447.28 | -29718.6 | -17814.3 | 8912.135 |
| (1,1,3) | 29189.33 | -14588.7 | 59447.9 | -29717.9 | -17820.8 | 8916.391 |
| (1,1,4) | 29180.6 | -14583.3 | 59446.95 | -29716.5 | -17843.2 | 8928.606 |
| (2,1,0) | 31113.52 | -15552.8 | 60564.36 | -30278.2 | -17780.4 | 8894.189 |
| (2,1,1) | 29258.17 | -14624.1 | 59448.99 | -29719.5 | -17815.5 | 8912.728 |
| (2,1,2) | 29198.3 | -14593.1 | 59447.14 | -29717.6 | -17803.7 | 8907.874 |
| (2,1,3) | 29132.91 | -14559.5 | 59450.7 | -29718.4 | -17822.7 | 8918.373 |
| (2,1,4) | 29133.34 | -14558.7 | 59451.88 | -29717.9 | -17816.9 | 8916.436 |
| (3,1,0) | 30275.81 | -15132.9 | 60184.52 | -30087.3 | -17781.3 | 8895.669 |
| (3,1,1) | 29145.78 | -14566.9 | 59449.25 | -29718.6 | -17815.3 | 8913.655 |
| (3,1,2) | 29143.48 | -14564.7 | 59451.89 | -29718.9 | -17817.7 | 8915.836 |
| (3,1,3) | 29126.56 | -14555.3 | 59450.65 | -29717.3 | -17978.9 | 8997.463 |
| (3,1,4) | 29136.89 | -14559.4 | 59454.83 | -29718.4 | -17886.5 | 8952.246 |
| (4,1,0) | 29811.58 | -14899.8 | 59944.87 | -29966.4 | -17804.5 | 8908.246 |
| (4,1,1) | 29140.29 | -14563.1 | 59449.84 | -29717.9 | -17849.1 | 8931.566 |
| (4,1,2) | 29111.85 | -14547.9 | 59453.17 | -29718.6 | -17994.3 | 9005.172 |
| (4,1,3) | 29144.29 | -14563.1 | 59456.06 | -29719 | -17837.4 | 8927.684 |
| (4,1,4) | 29125.98 | -14553 | 59416.99 | -29698.5 | -17975.2 | 8997.591 |

1. Patient 46

| ARIMA Model | ICP ARIMA AIC | ICP ARIMA LL | MAP ARIMA AIC | MAP ARIMA LL | PbtO2 ARIMA AIC | PbtO2 ARIMA LL |
| --- | --- | --- | --- | --- | --- | --- |
| (0,1,0) | 94880.52 | -47438.3 | 105056.2 | -52526.1 | 37360.48 | -18678.2 |
| (0,1,1) | 93829.03 | -46911.5 | 104812.5 | -52403.2 | 35745.4 | -17869.7 |
| (0,1,2) | 90040.86 | -45016.4 | 103640.2 | -51816.1 | 35133.66 | -17562.8 |
| (0,1,3) | 90042.17 | -45016.1 | 103110.4 | -51550.2 | 34983.9 | -17487 |
| (0,1,4) | 90044.08 | -45016 | 102658.8 | -51323.4 | 34927.96 | -17458 |
| (1,1,0) | 94635.49 | -47314.7 | 104895.6 | -52444.8 | 35235.43 | -17614.7 |
| (1,1,1) | 91628.58 | -45810.3 | 102806.1 | -51399 | 34957.1 | -17474.6 |
| (1,1,2) | 90042.17 | -45016.1 | 102638.3 | -51314.2 | 34928.9 | -17459.5 |
| (1,1,3) | 90044.28 | -45016.1 | 102634.6 | -51311.3 | 34928.42 | -17458.2 |
| (1,1,4) | 90014.97 | -45000.5 | 102471.3 | -51228.6 | 34923.06 | -17454.5 |
| (2,1,0) | 90757.58 | -45374.8 | 104288.9 | -52140.4 | 34932.13 | -17462.1 |
| (2,1,1) | 90681.81 | -45335.9 | 102645.2 | -51317.6 | 34931.82 | -17460.9 |
| (2,1,2) | 90044.16 | -45016.1 | 102607 | -51297.5 | 34920.36 | -17454.2 |
| (2,1,3) | 90010.66 | -44998.3 | 102607.2 | -51296.6 | 34932.35 | -17459.2 |
| (2,1,4) | 89933.42 | -44958.7 | 102170.8 | -51077.4 | 34910.09 | -17447 |
| (3,1,0) | 90731.69 | -45360.8 | 104240.9 | -52115.5 | 34931.44 | -17460.7 |
| (3,1,1) | 89832.97 | -44910.5 | 102647.1 | -51317.5 | 34933.11 | -17460.6 |
| (3,1,2) | 89742.68 | -44864.3 | 102605.7 | -51295.9 | 34934.66 | -17460.3 |
| (3,1,3) | 89744.6 | -44864.3 | 102609.1 | -51296.5 | 34923.14 | -17453.6 |
| (3,1,4) | 89746.43 | -44864.2 | 102170.7 | -51076.4 | 34916.55 | -17449.3 |
| (4,1,0) | 90450.3 | -45219.1 | 103850.5 | -51919.3 | 34932.07 | -17460 |
| (4,1,1) | 89748 | -44867 | 102319.2 | -51152.6 | 34934.84 | -17460.4 |
| (4,1,2) | 89744.61 | -44864.3 | 102229.4 | -51106.7 | 34901.15 | -17442.6 |
| (4,1,3) | 89746.69 | -44864.3 | 102186 | -51084 | 34915.62 | -17448.8 |
| (4,1,4) | 89748.4 | -44864.2 | 102170.7 | -51075.4 | 34919.06 | -17449.5 |

1. Patient 47

| ARIMA Model | ICP ARIMA AIC | ICP ARIMA LL | MAP ARIMA AIC | MAP ARIMA LL | PbtO2 ARIMA AIC | PbtO2 ARIMA LL |
| --- | --- | --- | --- | --- | --- | --- |
| (0,1,0) | 58985.26 | -29490.6 | 56434.84 | -28215.4 | 6778.167 | -3387.08 |
| (0,1,1) | 58494.34 | -29244.2 | 52845.55 | -26419.8 | 4493.39 | -2243.69 |
| (0,1,2) | 57652.45 | -28822.2 | 52721.59 | -26356.8 | 3706.548 | -1849.27 |
| (0,1,3) | 57573.11 | -28781.6 | 52700.28 | -26345.1 | 3410.957 | -1700.48 |
| (0,1,4) | 57574.84 | -28781.4 | 52700.59 | -26344.3 | 3240.368 | -1614.18 |
| (1,1,0) | 58732.12 | -29363.1 | 54549.57 | -27271.8 | 3053.908 | -1523.95 |
| (1,1,1) | 57684.15 | -28838.1 | 52740.14 | -26366.1 | 3028.306 | -1510.15 |
| (1,1,2) | 57575.87 | -28782.9 | 52706.87 | -26348.4 | 3012.877 | -1501.44 |
| (1,1,3) | 57574.82 | -28781.4 | 52701.11 | -26344.6 | 3011.822 | -1499.91 |
| (1,1,4) | 57576.87 | -28781.4 | 52692.48 | -26339.2 | 3007.425 | -1496.71 |
| (2,1,0) | 58092.83 | -29042.4 | 53403.16 | -26697.6 | 3022.21 | -1507.1 |
| (2,1,1) | 57576.07 | -28783 | 52698.78 | -26344.4 | 3018.665 | -1504.33 |
| (2,1,2) | 57576.28 | -28782.1 | 52700.56 | -26344.3 | 3016.343 | -1502.17 |
| (2,1,3) | 57576.65 | -28781.3 | 52688.78 | -26337.4 | 3016.597 | -1501.3 |
| (2,1,4) | 57578.71 | -28781.4 | 52692.6 | -26338.3 | 3006.211 | -1495.11 |
| (3,1,0) | 57814.11 | -28902.1 | 52999.73 | -26494.9 | 3015.34 | -1502.67 |
| (3,1,1) | 57574.98 | -28781.5 | 52700.53 | -26344.3 | 3016.605 | -1502.3 |
| (3,1,2) | 57579.61 | -28782.8 | 52702.77 | -26344.4 | 3013.13 | -1499.56 |
| (3,1,3) | 57576.4 | -28780.2 | 52576.6 | -26280.3 | 2976.505 | -1480.25 |
| (3,1,4) | 57579.22 | -28780.6 | 52643.39 | -26312.7 | 3000.722 | -1491.36 |
| (4,1,0) | 57701.13 | -28844.6 | 52854.31 | -26421.2 | 3011.357 | -1499.68 |
| (4,1,1) | 57510.85 | -28748.4 | 52702.52 | -26344.3 | 3000.064 | -1493.03 |
| (4,1,2) | 57448.74 | -28716.4 | 52695.7 | -26339.9 | 2966.035 | -1475.02 |
| (4,1,3) | 57452.48 | -28717.2 | 52694.08 | -26338 | 2971.265 | -1476.63 |
| (4,1,4) | 57446.73 | -28713.4 | 52542.84 | -26261.4 | 2959.692 | -1469.85 |
