# Supplementary material for: Evaluation of the relationship between slow-waves of intracranial pressure, mean arterial pressure and brain tissue oxygen in TBI: a CENTER-TBI exploratory analysis
Source: J Clin Monit Comput. 2020 May 16;35(4):711–22. doi: 10.1007/s10877-020-00527-6 (PMC8286934; doi:10.1007/s10877-020-00527-6)
Supplement: Supplementary file 2 — Supplementary file2 (DOCX 23 kb) [file 10877_2020_527_MOESM2_ESM.docx]

*Appendix B: Granger Causality Testing – ICP vs. MAP, PbtO_2_ vs. MAP, and PbtO_2_ vs. ICP – Individual Patient Analysis*

| **Patient** | ***F*-Statistic MAP on ICP** | ***p*-value** | ***F*-Statistic ICP on MAP** | ***p*-value** | ***F*-Statistic MAP on PbtO2** | ***p*-value** | ***F*-Statistic PbtO2 on MAP** | ***p*-value** | ***F*-Statistic ICP on PbtO2** | ***p*-value** | ***F*-Statistic PbtO2 on ICP** | ***p*-value** |
| --- | --- | --- | --- | --- | --- | --- | --- | --- | --- | --- | --- | --- |
| ***1*** | 453.03 | <0.0001 | 160.04 | <0.0001 | 904.87 | <0.0001 | 3.63 | 0.0058 | 531.24 | <0.0001 | 19.35 | <0.0001 |
| ***2*** | 165.75 | <0.0001 | 2.22 | <0.0001 | 15.17 | <0.0001 | 16.00 | <0.0001 | 45.26 | <0.0001 | 4.70 | 0.0009 |
| ***3*** | 468.50 | <0.0001 | 81.60 | <0.0001 | 3.64 | 0.0057 | 9.49 | <0.0001 | 22.60 | <0.0001 | 2.10 | 0.0784 |
| ***4*** | 580.30 | <0.0001 | 60.62 | <0.0001 | 5.32 | 0.0003 | 9.13 | <0.0001 | 1.76 | 0.1331 | 1.56 | 0.1816 |
| ***5*** | 58.33 | <0.0001 | 44.70 | <0.0001 | 5.34 | 0.0003 | 11.29 | <0.0001 | 19.15 | <0.0001 | 0.90 | 0.4647 |
| ***6*** | 137.84 | <0.0001 | 18.95 | <0.0001 | 0.11 | 0.9786 | 2.33 | 0.0538 | 11.94 | <0.0001 | 7.13 | <0.0001 |
| ***7*** | 796.36 | <0.0001 | 30.85 | <0.0001 | 139.54 | <0.0001 | 25.08 | <0.0001 | 197.81 | <0.0001 | 8.04 | <0.0001 |
| ***8*** | 120.16 | <0.0001 | 573.28 | <0.0001 | 655.43 | <0.0001 | 2.09 | 0.0795 | 18.55 | <0.0001 | 3.61 | 0.0061 |
| ***9*** | 295.73 | <0.0001 | 112.46 | <0.0001 | 865.65 | <0.0001 | 0.34 | 0.8496 | 230.78 | <0.0001 | 24.31 | <0.0001 |
| ***10*** | 588.31 | <0.0001 | 75.55 | <0.0001 | 59.59 | <0.0001 | 6.10 | <0.0001 | 149.49 | <0.0001 | 22.90 | <0.0001 |
| ***11*** | 309.27 | <0.0001 | 79.67 | <0.0001 | 27.46 | <0.0001 | 2.36 | 0.0511 | 9.53 | <0.0001 | 5.54 | 0.0002 |
| ***12*** | 1.95 | 0.1 | 64.06 | <0.0001 | 158.35 | <0.0001 | 2.29 | 0.0576 | 305.95 | <0.0001 | 20.58 | <0.0001 |
| ***13*** | 47.345 | <0.0001 | 16.63 | <0.0001 | 20.44 | <0.0001 | 4.58 | 0.0011 | 0.07 | 0.9904 | 0.25 | 0.9113 |
| ***14*** | 242.55 | <0.0001 | 366.89 | <0.0001 | 67.81 | <0.0001 | 1.94 | 0.10042 | 29.09 | <0.0001 | 3.88 | 0.0037 |
| ***15*** | 909.43 | <0.0001 | 19.40 | <0.0001 | 385.40 | <0.0001 | 21.29 | <0.0001 | 222.23 | <0.0001 | 9.57 | <0.0001 |
| ***16*** | 693.02 | <0.0001 | 165.66 | <0.0001 | 3.99 | 0.0031 | 7.843 | <0.0001 | 54.67 | <0.0001 | 9.85 | <0.0001 |
| ***17*** | 1243.22 | <0.0001 | 370.32 | <0.0001 | 62.99 | <0.0001 | 6.05 | <0.0001 | 383.13 | <0.0001 | 7.84 | <0.0001 |
| ***18*** | 419.69 | <0.0001 | 7.66 | <0.0001 | 109.30 | <0.0001 | 11.95 | <0.0001 | 1396.84 | <0.0001 | 30.60 | <0.0001 |
| ***19*** | 1114.69 | <0.0001 | 38.67 | <0.0001 | 12.86 | <0.0001 | 18.54 | <0.0001 | 140.74 | <0.0001 | 23.88 | <0.0001 |
| ***20*** | 550.38 | <0.0001 | 16.56 | <0.0001 | 36.55 | <0.0001 | 5.90 | <0.0001 | 84.06 | <0.0001 | 4.54 | 0.0012 |
| ***21*** | 248.96 | <0.0001 | 53.54 | <0.0001 | 236.63 | <0.0001 | 12.46 | <0.0001 | 271.99 | <0.0001 | 5.51 | 0.0002 |
| ***22*** | 1732.78 | <0.0001 | 703.16 | <0.0001 | 3.62 | <0.0001 | 1.43 | 0.2219 | 4.89 | 0.0006 | 0.67 | 0.6144 |
| ***23*** | 8884.88 | <0.0001 | 300.46 | <0.0001 | 206.09 | <0.0001 | 17.50 | <0.0001 | 286.86 | <0.0001 | 17.72 | <0.0001 |
| ***24*** | 462.078 | <0.0001 | 87.02 | <0.0001 | 5.19 | <0.0001 | 2.78 | 0.0252 | 11.76 | <0.0001 | 1.22 | 0.3018 |
| ***25*** | 764.87 | <0.0001 | 50.53 | <0.0001 | 137.82 | <0.0001 | 12.76 | <0.0001 | 52.73 | <0.0001 | 16.66 | <0.0001 |
| ***26*** | 354.81 | <0.0001 | 52.36 | <0.0001 | 55.17 | <0.0001 | 31.93 | <0.0001 | 86.14 | <0.0001 | 11.71 | <0.0001 |
| ***27*** | 3782.96 | <0.0001 | 263.69 | <0.0001 | 627.25 | <0.0001 | 97.25 | <0.0001 | 569.94 | <0.0001 | 105.95 | <0.0001 |
| ***28*** | 641.91 | <0.0001 | 98.84 | <0.0001 | 120.96 | <0.0001 | 3.60 | 0.0061 | 249.94 | <0.0001 | 17.87 | <0.0001 |
| ***29*** | 104.54 | <0.0001 | 165.87 | <0.0001 | 104.76 | <0.0001 | 21.46 | <0.0001 | 9.08 | <0.0001 | 26.53 | <0.0001 |
| ***30*** | 6041.45 | <0.0001 | 128.95 | <0.0001 | 269.95 | <0.0001 | 11.84 | <0.0001 | 172.51 | <0.0001 | 3.24 | 0.0114 |
| ***31*** | 2501.76 | <0.0001 | 136.27 | <0.0001 | 653.70 | <0.0001 | 20.71 | <0.0001 | 265.58 | <0.0001 | 158.29 | <0.0001 |
| ***32*** | 207.55 | <0.0001 | 84.52 | <0.0001 | 38.66 | <0.0001 | 7.20 | <0.0001 | 42.08 | <0.0001 | 7.65 | <0.0001 |
| ***33*** | 19.76 | <0.0001 | 47.73 | <0.0001 | 496.26 | <0.0001 | 5.27 | 0.0003 | 104.12 | <0.0001 | 5.07 | 0.0004 |
| ***34*** | 33.69 | <0.0001 | 25.10 | <0.0001 | 18.09 | <0.0001 | 0.75 | 0.5573 | 163.83 | <0.0001 | 9.91 | <0.0001 |
| ***35*** | 183.10 | <0.0001 | 30.42 | <0.0001 | 536.55 | <0.0001 | 51.64 | <0.0001 | 135.08 | <0.0001 | 15.69 | <0.0001 |
| ***36*** | 374.04 | <0.0001 | 203.82 | <0.0001 | 19.99 | <0.0001 | 4.68 | 0.0009 | 9.56 | <0.0001 | 2.70 | 0.0290 |
| ***37*** | 85.66 | <0.0001 | 10.69 | <0.0001 | 9.22 | <0.0001 | 3.77 | 0.0046 | 5.63 | 0.0002 | 8.90 | <0.0001 |
| ***38*** | 3619.41 | <0.0001 | 187.32 | <0.0001 | 2.58 | 0.0357 | 0.91 | 0.4573 | 6.649 | <0.0001 | 1.80 | 0.1249 |
| ***39*** | 5663.73 | <0.0001 | 181.21 | <0.0001 | 473.70 | <0.0001 | 4.12 | 0.0024 | 222.96 | <0.0001 | 18.35 | <0.0001 |
| ***40*** | 1148.29 | <0.0001 | 63.09 | <0.0001 | 519.82 | <0.0001 | 16.18 | <0.0001 | 1416.27 | <0.0001 | 122.26 | <0.0001 |
| ***41*** | 2338.75 | <0.0001 | 628.55 | <0.0001 | 129.10 | <0.0001 | 11.57 | <0.0001 | 75.08 | <0.0001 | 11.38 | <0.0001 |
| ***42*** | 249.89 | <0.0001 | 39.95 | <0.0001 | 48.95 | <0.0001 | 4.21 | 0.0021 | 247.97 | <0.0001 | 6.15 | <0.0001 |
| ***43*** | 830.86 | <0.0001 | 49.23 | <0.0001 | 4.01 | 0.003 | 9.02 | <0.0001 | 4.36 | 0.0016 | 3.14 | 0.0136 |
| ***44*** | 923.84 | <0.0001 | 157.20 | <0.0001 | 302.50 | <0.0001 | 102.97 | <0.0001 | 398.93 | <0.0001 | 10.2 | <0.0001 |
| ***45*** | 397.10 | <0.0001 | 67.31 | <0.0001 | 19.06 | <0.0001 | 23.33 | <0.0001 | 14.86 | <0.0001 | 29.44 | <0.0001 |
| ***46*** | 1138.65 | <0.0001 | 37.14 | <0.0001 | 159.07 | <0.0001 | 17.43 | <0.0001 | 138.44 | <0.0001 | 11.99 | <0.0001 |
| ***47*** | 202.30 | <0.0001 | 5.50 | 0.0002 | 148.60 | <0.0001 | 3.40 | 0.0086 | 44.56 | <0.0001 | 5.37 | 0.0003 |

*ICP = intracranial pressure, MAP = mean arterial pressure, PbtO_2_ = brain tissue oxygen. Table reports individual patient Granger causality testing with F-statistics and p-value for each directional test. Note: shaded cells are those relationships with the largest F-statistics and hence directional causality.*
